# Supplementary material for: Computer-aided X-ray screening for tuberculosis and HIV testing among adults with cough in Malawi (the PROSPECT study): A randomised trial and cost-effectiveness analysis
Source: PLoS Med. 2021 Sep 9;18(9):e1003752. doi: 10.1371/journal.pmed.1003752 (PMC8459969; doi:10.1371/journal.pmed.1003752)
Supplement: S1 Table — (DOCX) [file pmed.1003752.s001.docx]

**S1 Table: Characteristics of participants seen at day 56 visit compared to those lost to follow-up**

| **Characteristic** | **Seen at day 56 (n=1320)** | **Not seen at day 56 (n=142)** | **p-value^*^** |
| --- | --- | --- | --- |
| Age in years (mean, SD) | 33.8 (13.4) | 31.1 (13.4) | 0.02 |
| Sex |  |  |  |
| Male (n, %) | 578 (44%) | 54 (38%) | 0.19 |
| Female (n, %) | 742 (56%) | 88 (62%) |  |
| Body mass index (mean kg/m^2^, SD) | 22.8 (4.3) | 22.9 (4.2) | 0.86 |
| Marital status |  |  |  |
| Married/cohabiting (n, %) | 904 (68%) | 95 (67%) | 0.45 |
| Never married (n, %) | 205 (16%) | 27 (19%) |  |
| Widowed/separated/divorced (n, %) | 211 (16%) | 19 (13%) |  |
| Highest level of education |  |  |  |
| No schooling (n, %) | 157 (12%) | 22 (15%) | 0.18 |
| Primary (n, %) | 610 (46%) | 75 (53%) |  |
| Secondary no MSCE^†^ (n, %) | 357 (27%) | 29 (20%) |  |
| Secondary with MSCE^†^ (n, %) | 171 (13%) | 15 (11%) |  |
| Higher (n, %) | 25 (2%) | 1 (1%) |  |
| Ever lost a spouse to death | 119 (9%) | 11 (8%) | 0.61 |
| Literate | 1142 (87%) | 114 (80%) | 0.04 |
| Poverty quintile^ø^ |  |  |  |
| Quintile 1 (least poor) | 260 (20%) | 32 (23%) | 0.62 |
| Quintile 2 | 268 (20%) | 24 (17%) |  |
| Quintile 3 | 263 (20%) | 30 (21%) |  |
| Quintile 4 | 260 (20%) | 32 (23%) |  |
| Quintile 5 (poorest) | 269 (20%) | 24 (17%) |  |
| Tuberculosis symptoms |  |  |  |
| Cough (n %) | 1320 (100%) | 142 (100%) |  |
| Cough duration (median weeks, IQR) | 1 (0.6, 3) | 1 (0.4, 3) | 0.23 |
| Night sweats (n, %) | 543 (41%) | 57 (40%) | 0.82 |
| Weight loss (n, %) | 522 (40%) | 53 (37%) | 0.61 |
| Fever (n, %) | 665 (50%) | 80 (56%) | 0.18 |
| Previously treated for tuberculosis (n %) | 59 (4%) | 5 (4%) | 0.60 |
| HIV status |  |  |  |
| HIV-positive (n, %) | 262 (20%) | 18 (13%) | 0.01 |
| Taking ART (n, %) | 253 (97%) | 18 (100%) | 0.55 |
| HIV-negative (n, %) | 968 (73%) | 106 (75%) |  |
| Unknown (n, %) | 90 (7%) | 18 (13%) |  |
| EQ5D^§^ utility score (mean, SD) | 0.77 (0.14) | 0.79 (0.14) | 0.33 |
| Self-rated health |  |  |  |
| Fair/good/very good | 1167 (88%) | 129 (91%) | 0.39 |
| Poor/very poor | 153 (12%) | 13 (9%) |  |

^*^p-value from chi-squared test for categorical data, t-test for continuous data

^†^Malawi Secondary Certificate of Education
^§^EuroQOL EQ5D utility score (Zimbabwe tarrif)
^ø^Based on urban proxy means test using assets derived from 2014-15 Malawi Integrated Household Survey
IQR: interquartile range, SD: standard deviation
