# Supplementary material for: Computer-aided X-ray screening for tuberculosis and HIV testing among adults with cough in Malawi (the PROSPECT study): A randomised trial and cost-effectiveness analysis
Source: PLoS Med. 2021 Sep 9;18(9):e1003752. doi: 10.1371/journal.pmed.1003752 (PMC8459969; doi:10.1371/journal.pmed.1003752)
Supplement: S2 Table — (DOCX) [file pmed.1003752.s002.docx]

**S2 Table: Multiple imputation analysis of secondary outcomes with missing data**

|  | **Standard of care arm** | **HIV screening arm** | **HIV-TB screening arm** | **HIV screening vs. standard of care arm** | **HIV-TB screening vs. HIV screening arm** | **HIV-TB screening vs. standard of care arm** |
| --- | --- | --- | --- | --- | --- | --- |
| **Complete case analysis** |  |  |  | **RR, 95% CI** | **RR, 95% CI** | **RR, 95% CI** |
| Undiagnosed/untreated microbiologically-confirmed pulmonary TB (n, %) | 2/382 (0.5%) | 4/414 (1.0%) | 2/410 (0.5%) | 1.85 (0.34-10.02) | 0.50 (0.09-2.74) | 0.93 (0.13-6.58) |
| Undiagnosed/untreated HIV (n, %) | 10/377 (2.7%) | 2/414 (0.5%) | 1/415 (0.2%) | 0.18 (0.04-0.83) | 0.50 (0.05-5.48) | 0.09 (0.01-0.71) |
|  |  |  |  | **AMD (95% CI)** ^†^ | **AMD (95% CI)** ^†^ | **AMD (95% CI)** ^†^ |
| EQ5D^§^ utility score (mean, SD) | 0.79 (0.18) | 0.82 (0.19) | 0.81 (0.18) | 0.03 (0.01-0.05) | -0.002 (-0.03-0.02) | 0.03 (0.004-0.06) |
| **Multiple imputation analysis**^*^ |  |  |  | **RR, 95% CI** | **RR, 95% CI** | **RR, 95% CI** |
| Undiagnosed/untreated microbiologically-confirmed pulmonary TB (n, %) | 2/382 (0.5%) | 4/414 (1.0%) | 2/410 (0.5%) | 1.44 (0.30-6.91) | 0.57 (0.11-2.92) | 0.82 (0.14-5.00) |
| Undiagnosed/untreated HIV (n, %) | 10/377 (2.7%) | 2/414 (0.5%) | 1/415 (0.2%) | 0.22 (0.05-0.87) | 0.43 (0.04-4.35) | 0.09 (0.01-0.72) |
|  |  |  |  | **AMD (95% CI)** ^†^ | **AMD (95% CI)** ^†^ | **AMD (95% CI)** ^†^ |
| EQ5D^§^ utility score (mean, SD) | 0.79 (0.18) | 0.82 (0.19) | 0.81 (0.18) | 0.03 (0.003-0.05) | -0.002 (-0.03-0.02) | 0.03 (0.001-0.05) |

^†^Adjusted for baseline EuroQoL EQ5D utility score

*Run using chained equations with 50 imputations
