# Supplementary material for: Computer-aided X-ray screening for tuberculosis and HIV testing among adults with cough in Malawi (the PROSPECT study): A randomised trial and cost-effectiveness analysis
Source: PLoS Med. 2021 Sep 9;18(9):e1003752. doi: 10.1371/journal.pmed.1003752 (PMC8459969; doi:10.1371/journal.pmed.1003752)

**The PROSPECT Study**

*A pragmatic randomised study to optimise prevention, screening and care for tuberculosis in Malawi*

**Document:** Protocol

**Prepared by:** Peter MacPherson

Liverpool School of Tropical Medicine, UK &

Malawi-Liverpool-Wellcome Trust Clinical Research Programme

**Version:** 1.0: 2018-03-27

**Approved by:** Peter MacPherson
 Chief Investigator

**Approved on:** 2018-03-27

**Signature:**

**
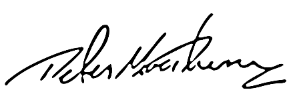
**

---------------------------------------------------------------------------------------------

# Table of Contents

[Table of Contents 2](#_Toc508720552)

[1 Regulatory 4](#_Toc508720553)

[1.1 Full title 4](#_Toc508720554)

[1.2 Short title 4](#_Toc508720555)

[1.3 Institutional review board references 4](#_Toc508720556)

[1.4 Trial registration number 4](#_Toc508720557)

[1.5 Sponsor 4](#_Toc508720558)

[1.6 Funding 4](#_Toc508720559)

[1.7 Conflicts of interest 4](#_Toc508720560)

[1.8 Compliance 4](#_Toc508720561)

[1.9 Investigators 5](#_Toc508720562)

[2 Abstract 6](#_Toc508720563)

[2.1 Background 6](#_Toc508720564)

[2.2 Study design 6](#_Toc508720565)

[2.3 Study site and participants 6](#_Toc508720566)

[2.4 Interventions 6](#_Toc508720567)

[2.5 Outcomes 7](#_Toc508720568)

[2.6 Statistical considerations 8](#_Toc508720569)

[2.7 Ethical issues 8](#_Toc508720570)

[2.8 Timelines 9](#_Toc508720571)

[2.9 Dissemination 9](#_Toc508720572)

[3 Background 10](#_Toc508720573)

[3.1 Burden of tuberculosis and HIV remain unacceptably high in sub-Saharan Africa 10](#_Toc508720574)

[3.2 Barriers to HIV/TB diagnosis and care 10](#_Toc508720575)

[3.3 Optimising TB triage testing and linkage to care 12](#_Toc508720576)

[3.4 Computer-assisted TB chest x-ray classification 13](#_Toc508720577)

[3.5 Health economics of TB/HIV screening 14](#_Toc508720578)

[3.6 Summary 14](#_Toc508720579)

[4 Research question, aims and objectives 15](#_Toc508720580)

[4.1 Aim 15](#_Toc508720581)

[4.2 Study hypothesis 15](#_Toc508720582)

[4.3 Objectives 15](#_Toc508720583)

[5 Study design 16](#_Toc508720584)

[6 Study site and population 17](#_Toc508720585)

[6.1 Study site 17](#_Toc508720586)

[6.2 Study population 17](#_Toc508720587)

[6.3 Inclusion criteria 17](#_Toc508720588)

[6.4 Exclusion criteria 17](#_Toc508720589)

[6.5 Identification of participants and recruitment 18](#_Toc508720590)

[7 Randomisation and Blinding 20](#_Toc508720591)

[7.1 Randomisation 20](#_Toc508720592)

[7.2 Blinding 20](#_Toc508720593)

[8 Interventions, methods and procedures 21](#_Toc508720594)

[8.1 All participants 21](#_Toc508720595)

[8.2 Group 1: Standard of care 21](#_Toc508720596)

[8.3 Group 2: Optimised HIV testing and treatment linkage 21](#_Toc508720597)

[8.4 Group 3: Optimised TB diagnosis, HIV screening and treatment linkage 22](#_Toc508720598)

[8.5 Piloting phase 22](#_Toc508720599)

[9 Additional nested study interventions 24](#_Toc508720600)

[9.1 Diagnostic accuracy of CAD4TB 24](#_Toc508720601)

[9.2 Validation of urinary LAM 25](#_Toc508720602)

[10 Outcome evaluation 26](#_Toc508720603)

[10.1 Outcome assessment: all participants 26](#_Toc508720604)

[10.2 Home Tracing 27](#_Toc508720605)

[10.3 Primary outcomes 27](#_Toc508720606)

[10.4 Secondary outcome 27](#_Toc508720607)

[10.5 Subgroup analysis 28](#_Toc508720608)

[10.6 Exploratory Bayesian analysis 28](#_Toc508720609)

[10.7 Definitions 29](#_Toc508720610)

[11 Adverse event data collection and management 31](#_Toc508720611)

[11.1 Institutional responsibilities 31](#_Toc508720612)

[11.2 Procedures 31](#_Toc508720613)

[11.3 Withdrawal from the trial 31](#_Toc508720614)

[12 Statistical considerations 32](#_Toc508720615)

[12.1 Sample size justification 32](#_Toc508720616)

[12.2 Statistical analysis 33](#_Toc508720617)

[12.3 Pre-specified sub-group analysis 34](#_Toc508720618)

[12.4 Bayesian analysis of primary trial outcome 34](#_Toc508720619)

[13 Economic evaluation 36](#_Toc508720620)

[13.1 Data handling and management 38](#_Toc508720621)

[14 Laboratory methods 39](#_Toc508720622)

[14.1 Sputum GeneXpert MTB/Rif testing 39](#_Toc508720623)

[14.2 Sputum smear and TB culture testing 39](#_Toc508720624)

[15 Study administration and monitoring 40](#_Toc508720625)

[15.1 Approvals 40](#_Toc508720626)

[15.2 Trial Sponsor and Insurance 40](#_Toc508720627)

[15.3 Trial Steering Committee 40](#_Toc508720628)

[15.4 Data, Safety and Monitoring Board 40](#_Toc508720629)

[16 Ethical considerations and regulations 41](#_Toc508720630)

[16.1 Informed consent procedures 41](#_Toc508720631)

[16.2 Ethical issues and potential harms 41](#_Toc508720632)

[16.3 Potential constraints 41](#_Toc508720633)

[16.4 Training provided 42](#_Toc508720634)

[17 Dissemination 43](#_Toc508720635)

[17.1 Dissemination 43](#_Toc508720636)

[17.2 Translating Research into Policy 43](#_Toc508720637)

[18 Study timeline 44](#_Toc508720638)

[19 Study budget 45](#_Toc508720639)

[20 References 47](#_Toc508720640)

[21 Appendix 1: Participant information sheets 49](#_Toc508720641)

[22 Appendix 2: Participant consent forms 50](#_Toc508720642)

[23 Appendix 3: Case record forms 52](#_Toc508720643)

[24 Appendix 3: Statistical analysis plan 53](#_Toc508720644)

[25 Appendix 4: Trial results tables 54](#_Toc508720645)

[26 Appendix 5: Letters of Support 58](#_Toc508720646)

# Regulatory

## Full title

A pragmatic randomised study to optimise screening, prevention and care for tuberculosis in Malawi (PROSPECT)

## Short title

The PROSPECT Study

## Institutional review board references

1. College of Medicine of Malawi Research Ethics Committee: P.11/17/2311
2. Liverpool School of Tropical Medicine Ethics Committee: 17-050

## Trial registration number

To be confirmed.

## Sponsor

Liverpool School of Tropical Medicine, Pembroke Place, Liverpool, UK, L3 5QA.

## Funding

The PROSPECT Study is funded by the Wellcome Trust, UK under a Career Development Clinical Research Fellowship awarded to the Chief Investigator.

## Conflicts of interest

The investigators declare no conflicts of interest.

## Compliance

The trial will be conducted in compliance with the protocol, ICH GCP Guidelines and other relevant regulatory requirements applying in Malawi.

## Investigators

| Investigator | Institute | Role |
| --- | --- | --- |
| Dr Peter MacPherson | Malawi-Liverpool-Wellcome Trust Clinical Research Programme, Malawi & Liverpool School of Tropical Medicine, UK | Chief Investigator |
| Prof Liz Corbett | Malawi-Liverpool-Wellcome Trust Clinical Research Programme, Malawi, College of Medicine of Malawi, and London School of Hygiene and Tropical Medicine, UK | Co-investigator |
| Dr Emily Webb | London School of Hygiene and Tropical Medicine, UK | Statistician |
| Dr Liz Joekes | University of Liverpool, UK | Radiologist |
| Dr Dama Phiri | Queen Elizabeth Central Hospital, Malawi | Radiologist |
| Prof Madhukar Pai | McGill University, Canada | Co-Investigator |
| Dr Marriott Nliwasa | Malawi-Liverpool-Wellcome Trust Programme, Malawi and College of Medicine of Malawi | Trial Physician |
| Dr Hendramoorthy Maheswaran | University of Liverpool, UK | Health Economist |
| Prof Bertie Squire | Liverpool School of Tropical Medicine, UK | Co-Investigator |
| Prof David Lalloo | Liverpool School of Tropical Medicine, UK | Co-Investigator |

# Abstract

## Background

Ambitious global targets have been set to eliminate tuberculosis as a public health problem by 2035. However, in Africa, where HIV has driven extremely high incidence rates, progress in reducing new infections and TB deaths remains too slow.

We have previously demonstrated that adults seeking diagnosis and treatment for TB and HIV face considerable barriers, and have long delays in starting treatment with high pre-treatment mortality.

Efforts to reduce TB mortality have been hampered by limitations in TB diagnostics, with considerable uncertainty about how available and new tests can be best implemented.

The aim of the PROSPECT Study is therefore to investigate the effectiveness and cost-effectiveness of optimised TB/HIV diagnosis and treatment linkage interventions on TB and HIV case detection, treatment initiation and mortality.

## Study design

A pragmatic open, three-arm individually-randomised controlled trial and economic evaluation will be conducted in one primary health care centre in Blantyre, Malawi, where HIV and TB are major contributors to early mortality.

## Study site and participants

The study will be conducted at one primary health clinic in Blantyre Malawi, where we have established HIV and TB research facilities and previously demonstrated high need for improved TB and HIV diagnosis. Sputum-based TB diagnostics (smear microscopy, and Xpert MTB/Rif) and treatment, and comprehensive HIV care (including provider-initiated HIV testing and antiretroviral therapy) are available free-to-cost to patients through the Malawi national TB and HIV programmes.

Participants will be adults with symptoms of pulmonary tuberculosis (cough of any duration) attending the primary clinic with an acute care episode. We will exclude adults who have taken treatment for TB within the previous 6-months, or who are taking isoniazid preventive therapy, or who do not live in Blantyre, or plan to move out of Blantyre.

## Interventions

Participants will be randomly allocated into one of three groups:

**Group 1 - Standard of care:** Participants will be seen by facility health workers and receive clinician-directed screening for HIV and TB according to Malawi national guidelines without further study input.

**Group 2 - Optimised HIV testing and treatment linkage**: Participants will be offered testing for HIV using rapid oral fluid kits by research assistants. Those with confirmed HIV infection will be linked to the HIV care clinic where facility healthworkers will screen for TB using standard sputum-based diagnostics without further study input.

**Group 3 - Optimised TB diagnosis, HIV screening and treatment linkage**: Participants will receive a high-throughput and high-sensitivity TB screening intervention, in addition to the HIV testing intervention. This will comprise of an initial digital chest x-ray classified by the CAD4TB image-recognition software as either "high probability of TB", or "low probability of TB". Participants whose x-rays are classified as having high probability of TB will receive confirmatory sputum testing with Xpert MTB/Rif cartridges, whilst participants whose x-rays have a low probability of TB will be referred to facility healthworkers for routine care.

All participants will be seen at the health facility at day 56, where they will be assessed to determine whether they are taking treatment for tuberculosis by inspection of medication, inspection of treatment cards, and inspection of facility TB registers. They will also be offered testing for HIV (if not on ART) and screened for TB, including by sputum culture, Xpert and smear microscopy.

## Outcomes

The **primary trial outcome** will be time in days – from Day 0 up to but not including Day 56 – to tuberculosis treatment initiation, evaluated at Day 56 following randomization. The trial is sufficiently powered to permit 3 pairwise comparisons between groups (i.e. Group 1 vs. 2; Group 2 vs. 3; and Group 1 vs. 3).

This three-arm pragmatic trial design allows us to efficiently answer two separate, important public health questions: firstly, by comparing Group 2 to Group 1, we should be able to determine whether HIV care should be prioritised for adults with TB symptoms. Additionally, by comparing Group 3 to Group 2, we will provide strong evidence for the effectiveness of an optimised and integrated HIV and TB diagnostic and treatment linkage approach.

The **secondary trial outcomes** will compare between pairs of groups:

- The proportion of randomised participants initiated onto tuberculosis treatment on the same day as randomisation, with the numerator being participants who were initiated on tuberculosis treatment on Day 0, and the denominator being all randomised participants.
- The proportion of randomised participants with undiagnosed/untreated microbiologically-confirmed pulmonary TB at Day 56, with the numerator being participants with microbiologically-confirmed tuberculosis (either sputum culture, or sputum Xpert, or sputum smear microscopy positive on a sample taken on Day 56) and who are confirmed not to be taking tuberculosis treatment on Day 56 (including participants who have previously initiated tuberculosis treatment, but have defaulted or stopped treatment – regardless of reason – for at least one week). The denominator will be all randomised participants.
- The proportion of randomised participants with undiagnosed/untreated HIV at Day 56, with the numerator being participants with positive confirmatory HIV test results at Day 56 and who are not taking antiretroviral therapy (regardless of previous HIV test results during or before the study period), and the denominator being all randomised participants.
- Time in days - from Day 0 up to but not including Day 56 - to initiation of antiretroviral therapy among participants with positive confirmatory HIV test results at Day 56 and who were not taking antiretroviral therapy at Day 0.
- The proportion of randomised participants reported to have died by Day 56, with the numerator being participants confirmed to have died through home tracing visits or TB treatment records, and the denominator being all randomised participants
- The proportion of TB cases with a successful TB treatment outcome. The numerator will be participants who were initiated onto tuberculosis treatment (either microbiologically-confirmed or clinically-diagnosed tuberculosis) up to, but not including Day 56, and who have a successful TB treatment outcome (either cured or completed treatment) at 6-months after starting treatment. The denominator will be all participants confirmed to have initiated tuberculosis treatment between Day 0 and up to, but not including Day 56.
- Mean difference in EuroQoL EQ5D utility score at Day 56, adjusting for participants’ EQ5D utility score measured at Day 0.
- Mean difference in EuroQoL EQ5D visual analogue scale score, adjusting for participants’ EQ5D visual analogue scale score measured at Day 0.
- Incremental cost-effectiveness per QALY gained

## Statistical considerations

Assuming 17% of participants in Group 1 initiate TB treatment by 8-weeks, and 5% loss to-follow-up, a sample size of n=1455 participants randomised in a 1:1:1 ratio across the three groups provides at least 80% power to detect a hazard ratio of 1.5 comparing Group 2 to Group 1, and a hazard ratio of 1.41 comparing Group 3 to Group 2.

## Ethical issues

This trial will be registered with an international trials registry prior to commencement of recruitment.

The Trial Sponsor is the Liverpool School of Tropical Medicine, Pembroke Place, Liverpool, UK.

Ethical approval to undertake the study has been granted by the institutional review boards of the Liverpool School of Tropical Medicine and the College of Medicine of Malawi (COMREC). All participants will be asked to provide informed written (or witnessed thumbprint if illiterate) consent.

Because of the nature of the study interventions, adverse events are anticipated to be rare. Nevertheless, we will systematically record, review and report to the trial sponsor all serious adverse events.

An independent Data and Safety Monitoring Board (DSMB) will be established and will meet regularly before and during the trial to review trial recruitment, endpoint accrual and adverse events. DSMB recommendations will be reported to the Trial Sponsor and institutional review boards.

## Timelines

Piloting and preparatory activities will commence in April 2018, and trial recruitment in June 2018. We anticipate recruiting participants over a 15-month period, with final outcome assessment of treatment outcomes and mortality conducted after 6-months. Thus, the study will be completed in July 2020.

## Dissemination

Trial results and findings from the cost-effectiveness analysis will be shared with Blantyre District Health Office, the Malawi National Tuberculosis Programme and with the Malawi National HIV Programme. We will report findings at national, regional and international conferences, and will submit a manuscript reporting trial findings to a peer-reviewed journal specialising in public health, HIV and tuberculosis.

To facilitate reproducibility of analysis, an anonymised minimal final dataset and all code required to reproduce analysis will be published in the trial GitHub repository.

# Background

## Burden of tuberculosis and HIV remain unacceptably high in sub-Saharan Africa

Tuberculosis (TB) is now the leading infectious cause of death worldwide^1^. In 2016, there were an estimated 1.4 million deaths attributed to tuberculosis global, with an additional 0.4 million deaths from TB among people living with Human Immunodeficiency Virus (HIV) infection^1,2^. The countries of sub-Saharan Africa have been disproportionately affected by the HIV-TB co-epidemics. Following extremely rapid increases in TB incidence, prevalence and deaths during the 1990s and 2000s in the region that occurred concurrently with rapid increases in population HIV prevalence^3^, TB rates have only begun to decline in the region in recent years^1^. Although the expansion of coverage of effective antiretroviral therapy (ART) for treatment of HIV in many sub-Saharan countries has likely contributed to recent reductions in mortality, the pace of decline is unacceptably slow.

New impetus has been given to efforts to improve tuberculosis control by the recent-agreed global End-TB Strategy^4^. This strategy, which was endorsed by WHO in 2015, demands global action and intensified research to address HIV-associated TB in 30-high HIV/TB burden countries that together comprise 87% of the global burden of TB^2^. Key targets for the End-TB strategy include achievement by 2035 of a 90% reduction in TB incidence and a 95% reduction in TB mortality compared to 2015^4^.

Modelling studies have shown however that the End-TB targets will not be met without a step-change in efforts to improve the early diagnosis and effective treatment of all individuals with TB^5^. Of concern remains low population TB case detection rates, and high case-fatality ratios, particularly among people living with HIV^6^.

## Barriers to HIV/TB diagnosis and care

Adults seeking care at health facilities in sub-Saharan Africa are an important group to address in TB care and prevention programmes, as they have high prevalence of undiagnosed TB^7^, a substantial burden of undiagnosed and untreated HIV^8^, and high mortality rates if not promptly diagnosed and linked to treatment^9^.

Our previous studies - similar to research from other countries in sub-Saharan Africa - has shown that the patient pathway from first health centre attendance, through diagnosis to successful treatment outcome is tortuous, with high rates of drop-out from care^8,10-12^. Importantly, as well as having high mortality rates, individuals with symptoms of pulmonary TB who are not rapidly diagnosed may continue to transmit TB to others in the community, further limiting control efforts.

**Figure 1: The TB care pathway**


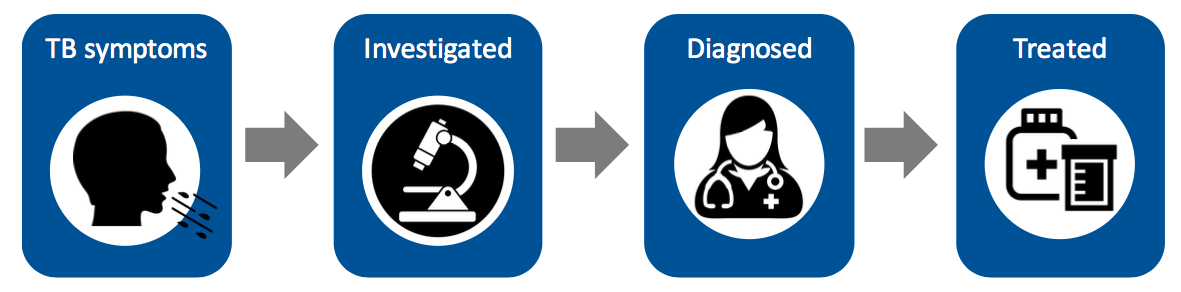


Although WHO guidelines promote intensified case-finding for TB among adults attending health facilities in high TB-prevalence settings^13^, implementation of routine screening for TB is known to be suboptimal in many settings. Exit interviews done with patients leaving health facilities has shown that clinicians rarely conduct an initial symptom screen^14,15^. Moreover, even when symptoms of presumptive TB are reported, only a small fraction receive appropriate investigations for TB^14,15^. Thus, even at the earliest step of the TB diagnostic and care pathway, there are high rates of loss from the cascade.

In addition to low rates of TB screening, we have previously shown that only a small proportion (13%) of adults attending health facilities receive HIV testing, despite WHO and Malawi guidelines recommending a strategy of universal provider-initiated HIV testing and counselling (HTC) for all individuals attending health centres, regardless of reason^8^. Uptake of HTC was highest among pregnant women attending for routine antenatal care, where considerable efforts have been undertaken to operationalise universal HTC as part of prevention of mother to child HIV transmission programmes. However, other groups, such as men and non-pregnant women have considerably lower rates of HIV testing completion^8^; as they are attending with an acute care episode and have a higher prevalence of active tuberculosis, they may have substantially worse outcomes compared to pregnant women.

Through systematic reviews, meta-analysis, and prospective cohort studies we have shown that even if patients attending health centres are diagnosed with TB or HIV, they face considerable barriers to treatment initiation. In Africa, 18% of adults with sputum smear-positive tuberculosis will not initiate tuberculosis treatment promptly^12^, whilst only a fifth of HIV-diagnosed adults will remain in care continuously to initiation of ART^10^. Across both conditions a number of common factors hindering access to treatment have been identified, including: requirements to make multiple health centre visits for registration and assessment visits; debility; competing demands, including from work and education; and high out-of-pocket costs associated with visiting health centres^16^.

When both HIV and TB are suspected or diagnosed, patients can face even greater challenges. Despite repeated calls for integration of HIV and TB care and prevention services, most clinic services remain vertically-organised. This means that patients are still often required to many health centre visits on different days of the week to receive HIV and TB assessments and treatment, multiplying their adverse case-seeking costs and potentially worsening outcomes^6,16^.

We, and other, have therefore argued that new approaches are required to improve integration of HIV and TB screening, prevention and care services in health facilities in Africa that can provide same-day, same-clinic diagnosis and treatment linkage for both conditions at minimum inconvenience to patients^16^. Such an approach, if effective, is likely to have large benefits for patients, public health, and for health systems by improving case detection and treatment access, reducing mortality, and mitigating the catastrophic costs associated with care-seeking. However, strong evidence for effectiveness obtained through robust randomised controlled trials is currently lacking.

## Optimising TB triage testing and linkage to care

Current TB screening approaches are reliant upon diagnostic tests with considerable limitations.

**Sputum smear microscopy** has been the mainstay of investigations for pulmonary tuberculosis for nearly a century. Although specificity is high, sensitivity remains unacceptably low, especially among HIV-positive adults^17,18^. Moreover, as sputum smears must be prepared, fixed and examined under light or fluorescence microscopy, infection control, quality control, throughput, and achievement of same-day diagnosis are challenging.

**TB culture** of sputum is slow (3-8 weeks, even with automated liquid culture systems), and relies upon availability of a high-quality laboratory. These requirements mean that whilst culture has importance for individual management of complex cases and for monitoring and evaluation, it is not practical as a point of care test^19^.

**GeneXpert MTB/Rif** is an integrated and automated cartridge-based nucleic acid amplification test that can provide results for the detection of *M tuberculosis* and associated rifampicin resistance within two hours^20^. There are two components to the test: the cartridge in which the biological sample is added to the assay, and a standalone unit in which cartridge is placed and where the nucleic acid amplification and detection takes place. The sensitivity of the Xpert assay is substantially higher than sputum smear microscopy^20^, and the newest version (Xpert Ultra) shows pooled sensitivity (among HIV-positive and HIV negative samples) that is 5% higher than the first generation assay, with a 12% gain among HIV-positive adults. WHO has endorsed GeneXpert MTB/Rif as the first line test among adults suspected to have multidrug resistant TB or HIV-associated TB.

Despite these advantages, there are some barriers to the widespread implementation of Xpert in low-resource, high TB prevalence settings. In particular, even at current concessional pricing for low-resource settings, Xpert is prohibitively expensive as a first line test for most national programmes.

**Chest radiography** has high sensitivity for pulmonary TB even in HIV coinfection^21,22^, and continues to play an important role in TB diagnosis in high-income settings. Although chest x-ray has been used for many years as a diagnostic tool (usually at the end of screening algorithms), widespread implementation in high prevalence settings has been limited by poor access to high quality equipment and expert radiologists, low specificity (leading to over-diagnosis of TB if chest x-ray alone is used) and high inter-reader variability^22^. Recent advances in digital chest x-ray technologies have reinvigorated interest in the use of chest x-ray as an initial triage tool in primary care in Africa.

**Potential benefits of digital chest radiography for TB^22^**

| Potential benefits |
| --- |
| Lower operating costs |
| Improved and more reproducible image quality with enlargement capability |
| Decreased radiation dose |
| Improved portable systems that can be used for mobile units |
| Potential for task-shifting to trained non-radiologists |
| The potential of objective tools for interpretation of digital images, such as computer-aided detection |
| Better (digital) archiving facilities |
| Film processing equipment and hard copies no longer required |
| Electronic transmission of images (for example for telemedicine or quality assurance) |

Chest X-ray may also be used as a triage test for TB^22^. In this triage approach, individuals with any abnormality identified on chest x-ray undergo confirmatory microbiological testing. Using a point-of-care high specificity molecular sputum testing for confirmatory testing (e.g. Xpert MTB/Rif) could allow accurate same-day TB diagnosis and treatment initiation in primary care.

In December 2016, WHO released a new evidence review and guidance^22^ for chest x-ray TB triage that used data from systematic review to model the potential effectiveness of TB screening algorithms, and showed that triage using chest x-ray, followed by GeneXpert MTB/Rif could substantially outperform other approaches.

**Figure 2: Modelled screening performance of TB screening algorithms^22^**


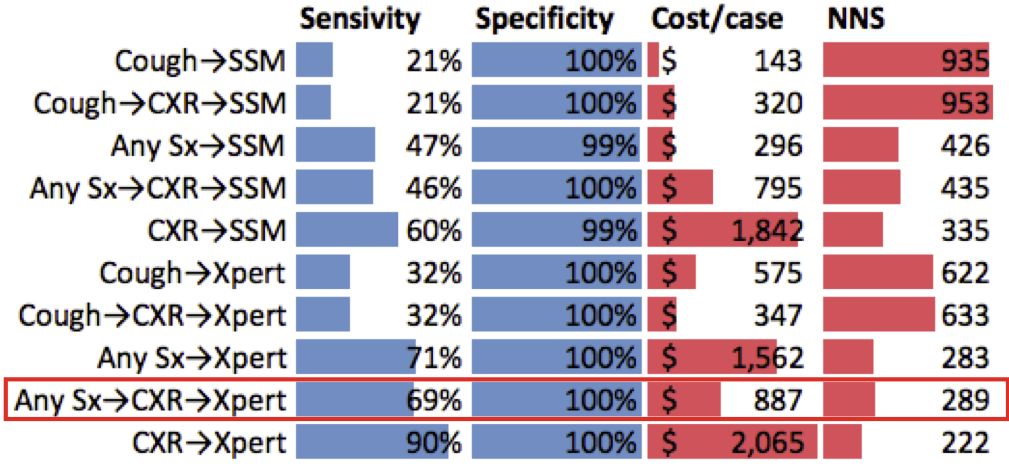


## Computer-assisted TB chest x-ray classification

Currently, countries such as Malawi have low coverage of radiology services, including trained radiologists. Computer-assisted detection (CAD) software - statistical algorithms used to classify digital images - is now available, and can be integrated within new digital x-ray units to provide immediate triage^23^. WHO recently systematically reviewed available evidence for one CAD system (CAD4TB, Delft Imaging Systems, Netherlands). Across 13 studies conducted in a variety of populations, sensitivity was as high as reading by radiologists, although specificity was lower necessitating microbiological confirmation of TB^22^. Whilst promising, WHO recommends that *"CAD can be used for TB detection for research, ideally following a protocol that contributes to the required evidence base for guideline development"^22^*.

**Diagnostic accuracy of CAD4TB in African studies^22^**

| Country | Software | N | HIV-positive | Sensitivity | Specificity |
| --- | --- | --- | --- | --- | --- |
| Zambia | CAD4TB c1.08 | 168 | 68% | 0.86 | 0.41 |
| Zambia | CAD4TB v1.08 | 350 | 54% | 1.00 | 0.23 |
| Tanzania | CAD4TB v3.07 | 894 | 41% | 0.95 | 0.33 |

*Data from systematic review (Pande et al 2016). At most sensitive abnormality threshold examined.*

In this trial, we will use CAD4TB as an initial triage test to classify digital chest x-rays as having either "low probability of tuberculosis", or "high probability of tuberculosis", prior to confirmatory bacteriological testing. In Blantyre, we have laid the groundwork for implementation of digital chest x-ray and CAD4TB screening. Prof Corbett is currently undertaking community-based TB screening in urban slums using the MinXray digital unit (MinXray Inc. USA) as part of her Wellcome Senior Fellowship cluster-randomised trial. In this research, we will install one MinXray unit in our established research facility at the study primary health care centre. Dr Joekes (expert academic radiologist) and the study team has worked closely with MinXray and with Delft Imaging Systems to install, pilot and validate CAD4TB in Blantyre.

## Health economics of TB/HIV screening

Timely detection of TB and/or HIV not only affects patient outcomes but also the costs of providing care. Delayed diagnosis of TB can result in more severe clinical presentation that may require hospitalisation. Timely initiation of ART in HIV-positive individuals will prevent HIV disease progression and development of severe HIV-associated co-morbidities. The direct health provider costs of managing acute hospital admission for severe TB, Cryptococcal meningitis and Pneumocystis Jivorecii pneumonia in Malawi are US$477, US$846 and US$325, respectively (in 2014 prices). In comparison, the cost per individual screened with CAD4TB has been estimated to be below US$2. Recent economic analysis of facility-based screening for chronic cough in high TB prevalent resource-poor settings found the use of CXR as TB triage testing would be cost-effective if the costs of the test were below US$2 per individual.

Despite the demonstrated utility of CXR and multiple WHO recommendations about its use, the programmatic and rational use of CXR for TB detection remains limited. We will undertake an economic evaluation to estimate the costs and cost-effectiveness of the proposed interventions. The findings will aide policy on the rationale use of CAD4TB, and optimisation of TB/HIV screening.

## Summary

In summary, adults with symptoms of tuberculosis in Malawi face considerable health systems delays, large out-of-pocket expenses, and have a high risk of mortality before diagnosis and treatment. To achieve the End-TB Strategy goals, a package of same-day, same-clinic diagnosis and treatment linkage interventions for both TB and HIV are urgently required. In an individually-randomised, open, three-arm controlled trial, The PROSPECT Study will investigate whether optimised TB and HIV diagnosis and treatment linkage interventions are cost-effective in reducing time to TB treatment initiation, and in improving case detection.

# Research question, aims and objectives

## Aim

The overall aim of the PROSPECT Study is to investigate the cost-effectiveness of optimised HIV and TB diagnosis and linkage to treatment on HIV and TB case detection, treatment initiation and mortality in Malawi.

## Study hypothesis

The PROSPECT Study will test the hypothesis that an optimised same-day TB/HIV screening and treatment linkage intervention for adults with presumptive tuberculosis in primary care could result in important improvements in case detection, treatment initiation and mortality.

## Objectives

1. Among adults with TB symptoms attending primary care in Malawi, to investigate the effectiveness of an optimised same-day screening algorithm consisting of rapid HIV testing, computer-assisted CAD4TB chest x-ray triage and, if abnormal, Xpert MTB/Rif rapid sputum molecular testing, and linkage to treatment.
2. In a nested diagnostic accuracy study evaluate the sensitivity and specificity of computer-assisted chest x-ray triage compared to classification by radiologists and bacteriological diagnosis.
3. Undertake a cost-utility analysis of the PROSPECT interventions to estimate the incremental cost per QALY gained from providing optimised TB and HIV diagnosis and linkage to care.

# Study design

PROSPECT is an open, three-arm individually-randomised controlled pragmatic trial. Following provision of information and receipt of informed consent, we will recruit adults with TB symptoms attending a primary health centre in Blantyre, Malawi with an acute care episode, who will be randomly allocated into one of three groups in a 1:1:1 ratio.

**Figure 3: Study design**


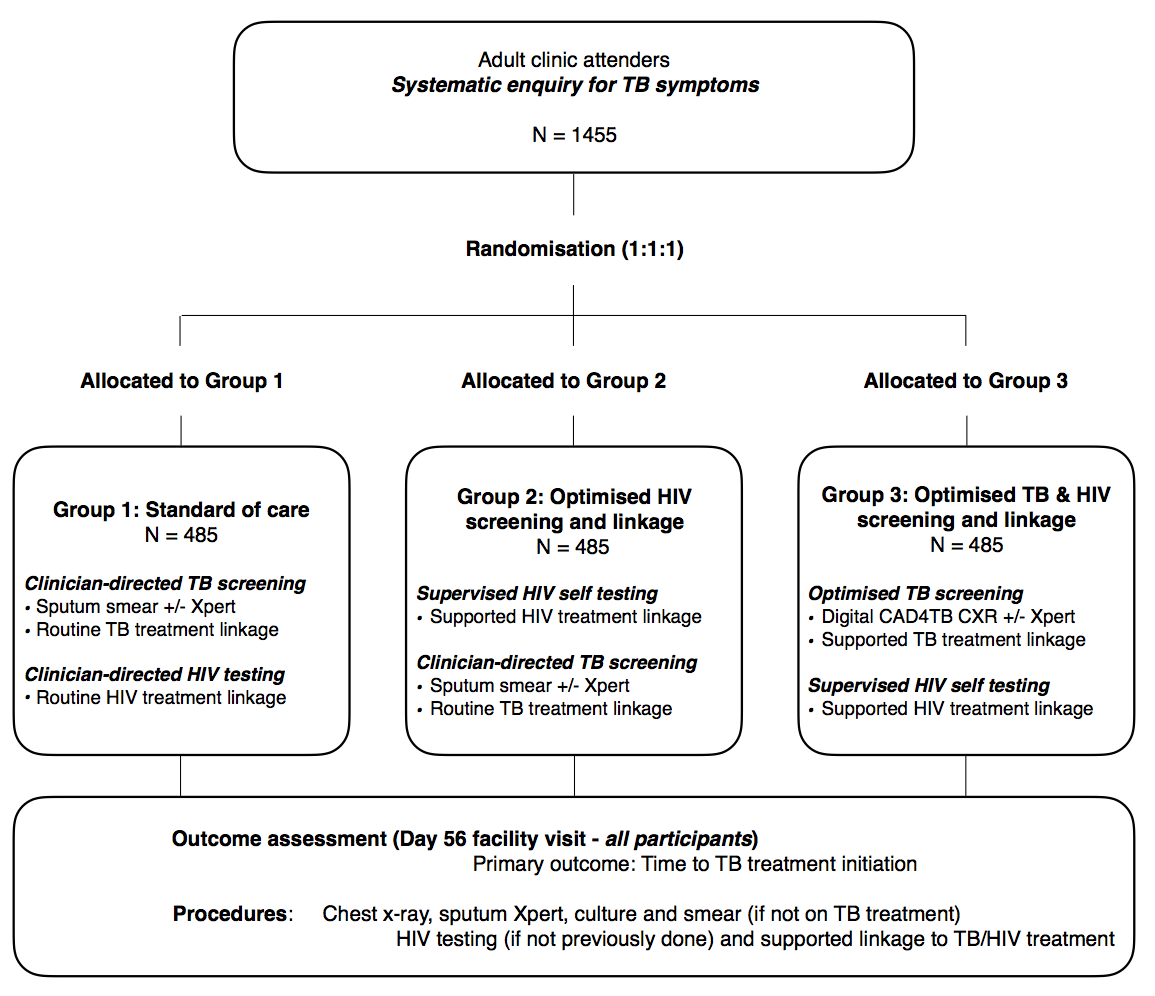


# Study site and population

## Study site

The PROSPECT Study will be conducted at one primary health care centre in Blantyre, Malawi, where we have previously undertaken research demonstrating need for improved TB and HIV diagnosis and linkage to care.

Blantyre has a number of busy urban neighbourhoods where adult HIV prevalence is estimated to be 18%^24^, and prevalence of active tuberculosis >900 per 100,000.

At the selected study primary health care centre, comprehensive HIV care will available through the national HIV programme, and will includes: routine provider initiated HIV testing and counselling, screening and treatment of opportunistic infections, provision of chemoprophylaxis, and treatment with antiretroviral therapy. Malawi National Guidelines currently recommend a "Test and Treat" approach to HIV, with all individuals diagnosed with HIV being eligible for antiretroviral therapy. Our previous research has shown however that coverage of HIV testing and rates linkage to antiretroviral therapy are suboptimal^8^, as in many other African settings.

TB diagnostics available at the selected study clinic will include sputum smear fluorescent microscopy with LED microscopes, and Xpert MTB/Rif. Although testing for tuberculosis is recommended for all individuals with symptoms of TB in Malawi and WHO guidelines (with Xpert preferred as the first line investigation for HIV-positive individuals), our previous research shows that TB diagnostics are underutilised^25^.

## Study population

Study participants will be adults with symptoms of pulmonary tuberculosis (cough of any duration) who attend the study primary health care clinic with an acute care episode. We will include pregnant women, as the diagnosis of TB is known to be suboptimal in this group.

As this is a pragmatic randomised trial that aims to provide evidence for policymakers under "real-life" conditions, eligibility criteria will be broad, and will reflect the characteristics of adults attending primary health centres with an acute care episode in Southern Africa to maximise generalisability.

## Inclusion criteria

1. Attends study health centre with an acute care episode
2. 18 years of age or older on the day of clinic attendance
3. Has symptoms of pulmonary tuberculosis (cough of any duration)
4. Is resident within urban Blantyre

## Exclusion criteria

1. Currently taking treatment for tuberculosis
2. Has taken any treatment for tuberculosis in the 6-months prior to clinic attendance
3. Is taking isoniazid preventive therapy
4. Plans to move out of Blantyre to live elsewhere in the following 6-months

## Identification of participants and recruitment

Participant and patient flow through the trial and clinic system are shown in Figure 4 below.

**Figure 4: Participant flow in PROSPECT Study**


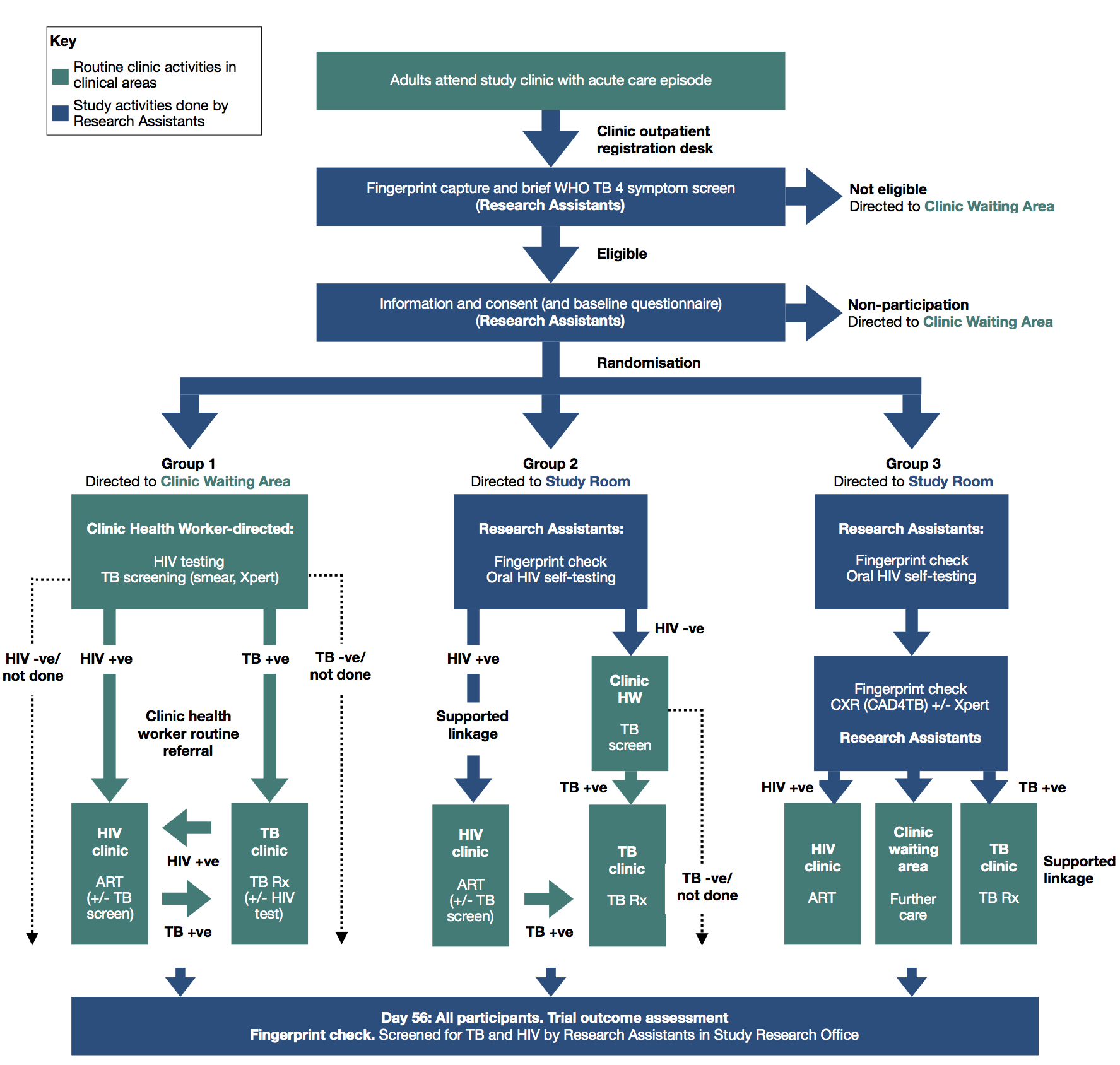


*TB: tuberculosis; HIV: Human immunodeficiency virus; HIV +ve: HIV-positive; HIV –ve: HIV-negative; TB +ve: TB positive; TB –ve: TB negative. Rx: treatment; CXR: chest x-ray; ART: antiretroviral therapy; HW: health worker*

Study Research Assistants will be based at the clinic registration desk where, using electronic data collection tablets, they will screen all daily acute clinic attenders for eligibility against inclusion and exclusion criteria (Case Record Form A: Eligibility Assessment & Consent). As adults may attend the clinic on more than one occasion during the study period, the Research Assistant will record a digital fingerprint (Simprints, Cambridge, UK) from all clinic attenders to ensure that repeat clinic attendance episodes are recorded and so removing the potential for duplication in trial recruitment.

Participants meeting inclusion criteria will be provided with oral and written information about the study by the research assistant (Appendix 1 Participant information forms) and will be invited to provide written informed consent (Appendix 2 Participant consent forms) to participate in the study. Participants who are illiterate will be asked to provide a thumbprint confirmation of consent, which will be observed and recorded by a witness independent of the study team. Completed consent forms will be securely stored as trial source documents within the trial folder.

# Randomisation and Blinding

## Randomisation

Eligible participants who provide consent to take part in the PROSPECT Study will be individually-randomly allocated in a 1:1:1 ratio into one of three groups.

Randomisation will be done by Research Assistants immediately following recruitment and using a random number computer programme running on study data-collection electronic tablets. To remove any potential for allocation bias, the participant's unique identifying number, randomisation code, time of randomisation and demographic characteristics will be automatically recorded and uploaded the trial database by cellular network, facilitating full reproducibility and audit of the randomisation and allocation process.

Using pre-written computer programmes, and without unblinding group allocation, the trial statistician and Chief Investigator will review on a weekly basis the numbers of participants allocated to each group to facilitate early identification of any issues with randomisation and allocation.

## Blinding

Because of the nature of the study and the interventions offered, it will not be possible to blind participants or research assistants to allocation groups. Nevertheless, extensive steps will be taken to ensure that Research Assistants undertaking day 56 outcome assessments are blinded to participants' group. Additionally, the investigators, including the Chief Investigator and Trial Statistician will remain blinded to allocation groups until database lock preceding final analysis. No unblinded interim analysis will be conducted.

# Interventions, methods and procedures

## All participants

All participants who provide written (or witnessed thumbprint) informed consent to participate in the study will complete a baseline questionnaire on Day 0, that will record demographic and clinical characteristics (including previous HIV and TB care), as well as geolocation information to facilitate home tracing using the ePALs geolocation system. All participants will additionally complete the EQ5D (Chichewa) tool to measure health-related quality of life.

Participants will be randomly allocated into one of three groups. Interventions provided to participants in each of the three trial groups are described below.

## Group 1: Standard of care

In Group 1 (**standard of care),** participants will be directed to wait in the clinic waiting area to be seen by facility health workers. The facility health workers will direct all further investigations and care **without any further input from the study team**. Facility health workers at the clinic will have access to the following services through the National HIV and TB programmes:

- HIV testing and counselling, provided by Facility HIV Testers in the clinic HIV and testing room, who follow a serial rapid fingerprick diagnostic kit-based algorithm recommended by WHO. Note that WHO and Malawian guidelines based on expert technical review recommend that only very brief pre-test HIV counselling is required.
- Routine TB screening, with both sputum smear microscopy and Xpert MTB/Rif testing available onsite at the clinic.
- Routine linkage to the onsite HIV clinic, where patients are registered and assessed for initiation onto antiretroviral therapy by facility HIV Care Clinic health workers. Malawi guidelines recommend universal treatment for HIV. HIV Care Clinic healthworkers will additionally have access to TB screening tests as described above; Malawi and WHO guidelines recommend that all HIV-positive individuals are assessed for TB on every healthcare encounter.
- Routine linkage to the onsite TB clinic, where patients are registered and initiated onto tuberculosis treatment. Malawi guidelines recommend universal HIV testing for all patients with confirmed TB.

## Group 2: Optimised HIV testing and treatment linkage

In Group 2, participants will be directed to the study room located in a separate building close to the main clinic building. Here, Study Research Assistants will validate the participant’s identity and trial allocation group by repeating the fingerprint scan that will validate identity and allocation group, and will offer a supervised HIV self-testing intervention, based on a model we have developed and implemented widely in Malawi and sub-Saharan Africa. Participants will be given brief pre-test instructions using previous developed materials and will be asked to self-test in a private area using the OraQuick 1/2 (OraSure Technologies) oral fluid HIV kit, before showing results to the study Research Assistant. Research assistants will provide supported self-testing should participants not want to self-test in private. WHO strongly recommends against detailed pre-test HIV counseling, except in exceptional circumstances. Participants will be supported to read their HIV test result by study Research Assistants and, if they have a reactive OraQuick result, will be provided with confirmatory fingerprick testing by the trained Research Assistants following serial algorithm recommended by WHO and the Malawi National HIV Programme. Following confirmatory testing,

- HIV-positive participants will be supported by Research Assistants to register at the onsite HIV care clinic, and all further care (including TB screening) will be directed by facility health workers **without any further study input**.
- HIV-negative participants will be referred to the clinic waiting area (with a copy of their HIV test results) to be seen by the facility health workers who will direct all further investigations (including TB screening) and care **without further study input**.

## Group 3: Optimised TB diagnosis, HIV screening and treatment linkage

Group 3 participants will be directed to the Study Room and will have their identity and trial allocation group validated by fingerprint scanning. They will be offered the HIV self-testing and linkage intervention as described above for Group 2.

Additionally, they will be offered a TB screening intervention comprising of:

- A digital chest x-ray performed by the study radiographer in the study room, and using the study MinXray unit.
- Chest x-rays will be immediately classified by the CAD4TB image-classification software running on the MinXray unit laptop as either “high probability of TB”, or “low probability of TB”.
- Participants whose chest x-rays indicate a low probability of TB will be referred to facility health workers (at either the onsite HIV care clinic if HIV-positive, or the clinic waiting area), with copies of their results for further routine care. All further investigations and care will be directed by facility health workers without further study input
- Participants whose chest x-ray are classified as having "high probability of TB" will be invited to submit a single sputum sample for testing by GeneXpert MTB/Rif (Cephaid, USA). Participants will be provided with a sputum cup and instructed in good sputum collection techniques using educational cards produced for a previous cluster randomised trial. They will be asked to expectorate sputum in a designated outdoor area with good air exchange outwith the study clinic room. Participants unable to spontaneously produce a sputum sample will be offered sputum induction using nebulised hypertonic saline. Sputum samples will be analysed using the GeneXpert MTB/Rif unit.
- Participants whose GeneXpert MTB/Rif results demonstrate the presence of *M tuberculosis* will be supported to register for tuberculosis treatment on the same day at the TB clinic within the study health facility.

## Piloting phase

Prior to trial recruitment, the study will be preceded by a 4-8-week piloting phase (see Study Timeline) to identifying any issues that require resolution, to finalise standard operating procedures, and to ensure data-collection systems are robust. No participants will be randomly allocated to interventions during this period.

Activities to be undertaken during the pilot phase will include:

1. Measurement of rates of clinic attendance and prevalence of TB symptoms stratified by sex and age group.
2. Evaluation of digital fingerprint identification system by recapture at clinic exit.
3. Estimation of rates of completion of routine TB screening by brief clinic exit interview.
4. Piloting of study questionnaire completion and data flow systems using digital tablets.
5. Piloting of oral swab HIV test kit use and confirmatory testing by Research Assistants.
6. Piloting of digital chest x-ray and CAD4TB system in adults with symptoms of TB.
7. Piloting of sputum collection procedures, and testing using GeneXpert Ultra systems.
8. Establishment and refinement of quality control procedures.

During the pilot phase, we will work closely with clinic staff to ensure that all participants diagnosed with TB or HIV are promptly linked to appropriate care, treatment and prevention services. We will ask individual patients participating in the pilot phase to provide written (or witnessed thumbprint) consent.

# Additional nested study interventions

## Diagnostic accuracy of CAD4TB

WHO has recommended that *"Computer aided diagnosis can be used for TB detection for research, ideally following a protocol that contributes to the required evidence base for guideline development"^22^*. The PROSPECT Study therefore offers opportunity to undertake a nested evaluation to contribute to the evidence base for the diagnostic accuracy (as well as effectiveness) of the CAD4TB platform.

Participants for this nested evaluation will be adults recruited to the main PROSPECT Study trial, and who complete a Day 56 outcome TB screening assessment. As part of this outcome assessment, all participants will undergo CAD4TB classification of digital chest x-ray, as well as sputum testing by GeneXpert using Ultra cartridge, and TB liquid automated culture. all digital chest x-rays taken from participants at outcome assessment will be uploaded to the password protected and secure MinXray online picture archiving and communication (PACS) radiology cloud server. All participant identifiers will be removed from x-rays prior to upload.

A panel of seven radiologists will each - independently and blinded to participant characteristics, HIV status, and results of microbiological investigations - classify chest x-rays using a standardised form for classification of chest radiology findings. Radiologists will review chest radiographs, and using an online data entry form, indicate the presence of any:

- Infiltrate or consolidation

- Cavitary lesion

- Nodule or mass with poorly defined margins

- Hilar/mediastinal adenopathy

- Pleural effusion

- Milliary findings

- Discrete linear opacity

- Discrete nodule(s) without calcification

- Other findings

Radiologists will additionally classify chest radiographs as either suggestive of active pulmonary tuberculosis, or not. We will use the kappa statistic (two outcomes, multiple readers) with 95% confidence intervals to assess inter-reader agreement among the radiologists.

For the diagnostic accuracy evaluation, the index test will be CAD4TB score (continuous variable ranging from 0 to 100, and in a secondary analysis, dichotomised for greater than or equal to the CAD4TB threshold score for high vs. low probability of tuberculosis, selected from pilot data and systematic analysis of previous CAD4TB diagnostic accuracy studies).

Reporting will follow the STARD Guidelines. For each index test definition (continuous distribution, and dichotomised to high vs. low probability of tuberculosis), we will compare diagnostic accuracy against two pre-defined reference standards:

1. Consensus radiologist classification with at least 5/7 independent readers agreeing that the radiograph was "suspicious of tuberculosis" (with sensitivity analysis limited to cases only where all 7/7 readers were in agreement), and
2. Bacteriologically-confirmed pulmonary tuberculosis, defined as either a documented positive GeneXpert result for *M. tuberculosis* on at least one sample of sputum taken for study purposes at Day 56 assessment; or documented growth of *M tuberculosis* and positive specialisation using MPT 64 antigen tests on at least one culture of sputum taken for study purposes at Day 56 assessment; or documented identification of acid fast bacilli on at least one sputum sample taken for study purposes and examined by sputum smear microscopy at Day 56 assessment.

For each comparison, sensitivity, specificity, positive predictive value, negative predictive value, and diagnostic odds ratios will be reported. Additionally, by constructing logistic regression models, we will investigate the effect of reader characteristics on diagnostic accuracy with bacteriological-confirmation as the reference standard.

## Validation of urinary LAM

Urine lipoarabanimannan testing (LAM) is a relatively new tuberculosis diagnostic, that has high accuracy among adults with advanced HIV infection, and has been shown to reduce mortality in hospitalised HIV-positive adults^9^. The test is based on a lateral flow assay, and has been constructed to be used a point of care test, with results read at the bedside.

However, sensitivity is known to be suboptimal among ambulant TB suspects. A newer version of the urine LAM test (FIND/Fujifilm) has been reported to have high sensitivity for TB, even among ambulant adults, and HIV-negative individuals. Therefore, the PROSPECT Study offers opportunity to undertake a nested evaluation of the performance of this test. We will collect a 5ml sample of urine from all participants at baseline, and transport the sample to the TB laboratory at the College of Medicine of Malawi for urine LAM testing. We will compare the diagnostic yield of urine LAM testing with that of sputum culture, smear and Xpert from day 56 participant samples.

# Outcome evaluation

## Outcome assessment: all participants

Following completion of trial interventions, all participants in each of the three groups will be given a written appointment card to attend a follow-up assessment at the study research clinic room 56 days after randomisation. They will be also issued with a voucher that they can use to reimburse the cost of transport to the clinic for this assessment.

To evaluate the primary trial outcome (time to tuberculosis treatment initiation), we will undertake a detailed questionnaire (Case Record Form G: Day 56 Questionnaire) to record the date of TB treatment initiation, and verify by inspecting participant-carried TB treatment cards, medication bottles, and facility registers.

To evaluate the prevalence of undiagnosed tuberculosis, we will collect two samples of sputum from all participants. Participants will be provided with a sputum cup and instructed in good sputum collection techniques using educational cards produced for a previous cluster randomised trial. They will be asked to expectorate sputum in a designated outdoor area with good area exchange outside the study clinic room. Participants unable to spontaneously produce a sputum sample will be offered sputum induction using nebulised hypertonic saline.

Samples will be transported to the tuberculosis laboratory at the College of Medicine of Malawi, where they will be cultured for tuberculosis using the MGIT system, undergo smear examination using fluorescence microscopy, and tested using the Xpert MTB/Rif assay. Positive TB results will be reported to participants within three days of receipt (including by home tracing), and participants will be supported to register for TB treatment at the health facility. We will additionally perform a digital chest x-ray for all participants, although this will not be used for ascertainment of TB status, but to support clinicians in providing further clinical care if required, and for the nested diagnostic accuracy study. All participants requiring additional care will be supported to access either the HIV clinic, TB clinic, or outpatient clinic at the study health clinic as required. Additionally, we will support referral and access to Queen Elizabeth Centre Hospital should further specialist care be required.

To evaluate the prevalence of undiagnosed HIV, we will offer all participants HIV testing, unless they are confirmed to be taking antiretroviral therapy. A testing algorithm as recommended by WHO for high prevalence settings will be followed. The initial HIV test will comprise OraQuick OraSure HIV 1/2, if positive followed by confirmatory fingerprick Determine 1/2. If tests are discordant, they will both be repeated. In the event of continued discordance, a third test will be done (Unigold). Where status is unable to be resolved (anticipated <1 per 10,000 participants), this HIV testing will be advised after two weeks as recommended by WHO guidance.

To evaluate health-related quality of life (HRQoL), we will use the EQ-5D-3L. The EQ-5D is a generic HRQoL measure comprised of a series of questions used to elicit a utility score and a visual analogue scale, and was translated into Chichewa following international and EuroQoL guidelines. The EQ-5D-3L tool will be administered to all trial participants at baseline, and on Day 56.

To estimate TB treatment outcomes at 6-months, we will use prospectively-collected TB treatment cohort data collected for the Blantyre Enhanced TB Surveillance Programme. On a quarterly basis TB treatment registers from all TB treatment centres in Blantyre are captured electronically and reconciled. In the PROSPECT Study, when a participant initiates TB treatment, we will record their national TB treatment number in the study database, and use this unique identifier to link participants to their TB treatment outcome data.

## Home Tracing

Participants who do not attend their scheduled day 56 outcome assessment will be contacted initially by telephone and encouraged to attend. After three phone call attempts, up to three home tracing visit will be undertaken by Research Assistants. Home tracing will be facilitated by the use of our validated high accuracy ePALs geolocation system. Mortality will be ascertained by verbal report from caregivers.

## Primary outcomes

The primary trial outcome will be time in days – from Day 0 up to but not including Day 56 – to tuberculosis treatment initiation, evaluated at Day 56 following randomization.

Analysis of the primary outcome will be done on an intention to treat basis, with all participants analysed according to the group to which they were randomised. Time to TB treatment outcome analysis will be right censored at Day 56 from randomisation if TB treatment is not initiated, or at day of loss to follow-up. We will make three pair-wise comparisons *(Group 2 vs. Group 1; Group 3 vs. Group 2; and Group 3 vs. Group 1)*.

This primary endpoint has been chosen because reducing time to initiation of treatment could have important individual and public health benefits. Assessment over eight weeks has been selected because: (i) TB culture is typically completed within 8 weeks, (ii) mortality is highest during this period, and (iii) previous trials and our pilot data show that TB treatment initiations plateau by 8- weeks.

## Secondary outcome

The secondary trial outcomes will be:

- The proportion of randomised participants initiated onto tuberculosis treatment on the same day as randomisation, with the numerator being participants who were initiated on tuberculosis treatment on Day 0, and the denominator being all randomised participants.
- The proportion of randomised participants with undiagnosed/untreated microbiologically-confirmed pulmonary TB at Day 56, with the numerator being participants with microbiologically-confirmed tuberculosis (either sputum culture, or sputum Xpert, or sputum smear microscopy positive on a sample taken on Day 56) and who are confirmed not to be taking tuberculosis treatment on Day 56 (including participants who have previously initiated tuberculosis treatment, but have defaulted or stopped treatment – regardless of reason – for at least one week). The denominator will be all randomised participants.
- The proportion of randomised participants with undiagnosed/untreated HIV at Day 56, with the numerator being participants with positive confirmatory HIV test results at Day 56 and who are not taking antiretroviral therapy (regardless of previous HIV test results during or before the study period), and the denominator being all randomised participants.
- Time in days - from Day 0 up to but not including Day 56 - to initiation of antiretroviral therapy among participants with positive confirmatory HIV test results at Day 56 and who were not taking antiretroviral therapy at Day 0.
- The proportion of randomised participants reported to have died by Day 56, with the numerator being participants confirmed to have died through home tracing visits or TB treatment records, and the denominator being all randomised participants
- Mean difference in EuroQoL EQ5D utility score at Day 56, adjusting for participants’ EQ5D utility score measured at Day 0.
- The proportion of TB cases with a successful TB treatment outcome. The numerator will be participants who were initiated onto tuberculosis treatment (either microbiologically-confirmed or clinically-diagnosed tuberculosis) up to, but not including Day 56, and who have a successful TB treatment outcome (either cured or completed treatment) at 6-months after starting treatment. The denominator will be all participants confirmed to have initiated tuberculosis treatment between Day 0 and up to, but not including Day 56.
- Mean difference in EuroQoL EQ5D visual analogue scale score, adjusting for participants’ EQ5D visual analogue scale score measured at Day 0.
- Incremental cost-effectiveness per QALY gained (see Economic Analysis Protocol for full details of measurement and analysis).

## Subgroup analysis

In pre-planned subgroup analysis, we will stratify analysis of the primary trial outcome by:

1. Sex (male vs. female). In this analysis, we will compare between pairs of groups the time to tuberculosis treatment for male and female participants.
2. Microbiological status (microbiologically-confirmed TB vs. clinically-diagnosed TB). In this analysis, we will compare between pairs of groups the time to tuberculosis treatment for participants with microbiologically-confirmed and clinically-diagnosed TB.

## Exploratory Bayesian analysis

We will additionally undertake a Bayesian analysis of the primary trial outcome. Prior distributions for the proportion of participants initiating TB treatment will be elicited from key stakeholder groups. We anticipate that key stakeholders will include:

**Key stakeholders identified for Bayesian prior elicitation exercise**

| Stakeholder group | How identified |
| --- | --- |
| Community members/trial participants | Through MLW Community Engagement Officer |
| Clinicians | Through existing links with DHO and health centre/hospital clinicians |
| Researchers | Through MLW Research Group Leads |
| Experts (TB/HIV) | Through Trial Investigators’ contacts |
| Policymakers (Malawi, Regional, International) | Through Trial Investigators’ existing links with individuals and policy groups |

Key stakeholders will be invited to attend workshop meetings, where they will be introduced to the study design and interventions through presentations and group discussions. To elicit prior beliefs for effect of interventions, we will use a “bin-and-chip” method, implemented within an interactive web application:

(<https://pmacp.shinyapps.io/bayesian_prior_elicit_prospect/>).

Before eliciting stakeholders’ prior beliefs for trial interventions, we will provide a series of “warm-up” vignettes based around familiar events such as the probability of a football team winning, or the probability of it raining tomorrow. This will allow stakeholders to become familiar with the process of assigning probability to future events, and with completing the interactive web application.

Each stakeholder will then be asked to make ten guesses for the proportion of participants initiating tuberculosis treatment using the interactive web application. Responses will be saved directly into a dedicated study database.

## Definitions

**Microbiologically-confirmed tuberculosis** will be defined by:

A participant with: a documented positive GeneXpert MTB/Rif result for *M. tuberculosis* on at least one sample of sputum taken for study or routine clinical purposes; or documented growth of *M. tuberculosis* and positive speciation using MPT 64 antigen tests on at least one culture of sputum taken for study or routine clinical purposes; or documented identification of acid fast bacilli on at least one sputum sample taken for study or routine clinical purposes and examined by sputum smear microscopy.

**Clinically-diagnosed tuberculosis** will be defined by:

A participant who does not fulfil the criteria for bacteriological confirmation but has documented evidence of having been diagnosed with active TB by a clinician or other medical practitioner who has decided to give the patient a full course of TB treatment. This definition includes cases diagnosed on the basis of X-ray abnormalities or suggestive histology and extrapulmonary cases without laboratory confirmation. Clinically-diagnosed cases that are subsequently found to be bacteriologically-positive (before or after starting treatment) will be reclassified as bacteriologically-confirmed.

**Pulmonary tuberculosis (PTB)** will be defined by:

A participant with bacteriologically-confirmed or clinically diagnosed case of TB involving the lung parenchyma or the tracheobronchial tree. Miliary TB will be classified as PTB because there are lesions in the lungs. Tuberculous intra-thoracic lymphadenopathy (mediastinal and/or hilar) or tuberculous pleural effusion, without radiographic abnormalities in the lungs, constitutes a case of extrapulmonary TB. A patient with both pulmonary and extrapulmonary TB will be classified as a case of PTB.

**Extrapulmonary tuberculosis (EPTB)** will be defined by:

A participant with bacteriologically-confirmed or clinically-diagnosed case of TB involving organs other than the lungs, e.g. pleura, lymph nodes, abdomen, genitourinary tract, skin, joints and bones, meninges.

**Initiation of tuberculosis treatment** will be defined by:

A participant in whom there is documented evidence of commencement of anti-tuberculosis treatment, either by: inspection of the participant-carried national tuberculosis treatment card; or inspection of the facility tuberculosis treatment register; or inspection of TB treatment medication bottles or pill boxes.

**Initiation of antiretroviral therapy** will be defined by:

A participant in whom there is documented evidence of commencement of combination antiretroviral therapy treatment, either by: inspection of the participant-carried national HIV programme treatment card; or inspection of the facility antiretroviral therapy treatment register; or inspection of antiretroviral therapy medication bottles or pill boxes.

**Successful tuberculosis treatment outcome** will be defined by:

A participant in whom tuberculosis treatment is initiated for bacteriologically-confirmed pulmonary tuberculosis, and who has documented evidence in their national tuberculosis treatment card of being cured of TB, being either sputum smear- or culture-negative in their last month of treatment and on at least one previous occasion, *or*

A participant with documented evidence of having completed TB treatment without evidence of failure (that is sputum smear- or culture-positive at month 5 or later during treatment) but with no record to show that sputum smear or culture results in the last month of treatment and on at least one previous occasion were negative, either because tests were not done or because results are unavailable.

# Adverse event data collection and management

As this is a pragmatic randomised trial and no new investigational products are being evaluated, we anticipate only a small number of adverse events. Nevertheless, we will ensure that case definitions, standardised operating procedures and a reporting protocol will be in place to record all adverse events.

## Institutional responsibilities

Adverse events will be reported immediately to the Chief Investigator and the trial physician (Dr Nliwasa, and will be logged and reported through regular follow-up reports (Case Record Form M: Adverse event).

A 3-monthly progress report will report on safety as well as other important process indicators and will be sent to the trial steering committee members, data and safety monitoring board members and collaborators in Ministry of Health of Malawi and Blantyre District Health Office.

## Procedures

Adverse event forms will be completed by the Field Trial Coordinator and reported to the Chief Investigator and the Trial Physician. The Chief Investigator will check the form, make changes as necessary, sign and email a scanned copy of the form to the IRBs and Sponsor.

In all cases participants experiencing adverse events will be followed up until final clinical outcome has been established (complete clinical recovery, or clinical condition has stabilised).

Adverse events will be evaluated for seriousness, and likely causality by the Chief Investigator and the Trial Physician for final classification.

The following adverse events will be systematically recorded and reported:

- Misclassification or misinterpretation of results leading to a participant starting TB therapy in error
- Misclassification or misinterpretation of results leading to a participant starting HIV treatment in error
- Breach of confidentiality following TB or HIV diagnosis
- Needlestick injuries

## Withdrawal from the trial

Participants will be informed that they are free to withdraw from the trial at any time should they desire, and without affecting their routine clinical care received. Reasons for withdrawal will be recorded and reported to the DSMB (Case Record Form N: Study termination).

# Statistical considerations

## Sample size justification

The primary trial outcome will be the time to tuberculosis treatment initiation among all randomised participants. To evaluate the relative effects of the HIV and TB screening/linkage interventions, we wish to make *k=3* pairwise comparison *(Group 2 vs. Group 1; Group 3 vs Group 2; and Group 3 vs Group 1)*.

Our pilot data from Blantyre health centres show that 17% of 190 adults with TB symptoms initiated TB treatment under standard of care conditions within 56 days.

First, we estimate the sample size required for 80% power to detect various hazard ratios at 5% significance level, comparing Group 2 vs. Group 1. In sample size calculations, we use a formula for the proportional hazards model developed by Schoenfield. We assume 17% of participants in Group 1 will initiate TB treatment, as per pilot data.

**Sample size estimates, comparing Group 2 vs. Group 1**

| **Cumulative hazard of TB treatment (Group 1)** | **Hazard ratio (Group 2 vs. 1)** | **Total number of events** | **Total N**  **(Groups 2 & 1)** |
| --- | --- | --- | --- |
| 0.17 | 1.30 | 456.10 | 2369 |
| 0.17 | 1.35 | 348.60 | 1777 |
| 0.17 | 1.40 | 277.31 | 1388 |
| 0.17 | 1.45 | 227.41 | 1119 |
| 0.17 | 1.50 | 190.97 | 923 |
| 0.17 | 1.55 | 163.46 | 777 |
| 0.17 | 1.60 | 142.12 | 665 |

*Using formula for the proportional hazards model developed by Schoenfield*

Now we estimate the sample size required for 80% power to detect various hazard ratios at 5% significance level, comparing Group 3 vs. Group 2.

**Sample size estimates, comparing Group 3 vs. Group 2**

| **Cumulative hazard of TB treatment (Group 2)** | **Hazard ratio (Group 3 vs. 2)** | **Total number of events** | **N (Groups 3 & 2)** |
| --- | --- | --- | --- |
| 0.255 | 1.39 | 289.517 | 981 |
| 0.255 | 1.40 | 277.312 | 936 |
| 0.255 | 1.41 | 265.942 | 895 |
| 0.255 | 1.42 | 255.331 | 856 |
| 0.255 | 1.43 | 245.410 | 820 |

*Using formula for the proportional hazards model developed by Schoenfield. Assuming hazard ratio of 1.5 comparing Group 2 vs. Group 1.*

Finally, assuming participants are allocated in a 1:1:1 ratio across the three groups, and inflating by 5% to account for loss to follow-up, we estimate the overall sample size required.

**Sample size estimates for pairwise comparisons**

| **Baseline hazard (Group 1)** | **Hazard ratio detectable (Group 2 v 1)** | **Sample size (Groups 1 & 2)** | **Baseline hazard (Group 2)** | **Hazard ratio detectable (Group 3 v 2)** | **Sample size (Groups 2 & 3)** | **Allocated in 1:1:1 ratio across three groups, and with 5% loss to follow-up** |
| --- | --- | --- | --- | --- | --- | --- |
| 17% | 1.40 | 1388 | 25.5% | 1.39 | 981 | 2187 |
| 17% | 1.45 | 1119 | 25.5% | 1.40 | 936 | 1763 |
| 17% | 1.50 | 923 | 25.5% | 1.41 | 895 | 1455 |
| 17% | 1.55 | 777 | 25.5% | 1.42 | 856 | 1224 |
| 17% | 1.60 | 665 | 25.5% | 1.43 | 820 | 1049 |

*With power=80% and loss to follow-up of 5%. Assuming Group 2 vs. Group 1 HR=1.50*

Therefore, adjusting for 5% loss to follow-up, a total sample size of 1455 participants (485 per group, = 923/2 x 3 x 1.05) gives at least 80% power to detect at least a cumulative hazard ratio for TB treatment initiation of 1.5 comparing Group 2 to Group 1, and a hazard ratio of 1.41 comparing Group 3 to Group 2. Additionally, under these assumptions, 485 participants per group would give 80% power to detect a hazard ratio of at least 1.50 comparing Group 3 to Group 1.

## Statistical analysis

All statistical analysis will be conducted in accordance with a pre-published statistical analysis plan. Trial reporting will follow CONSORT Guidelines.

We will report baseline characteristics of randomised participants, stratified by allocated group.

Analysis of the primary and secondary outcomes will be done on an intention to treat basis, with all participants allocated to trial groups included and analysed in the group to which they were randomized (regardless of which intervention was received). We do not anticipate imbalance between groups, so unadjusted effect estimates will be reported. Participants who have missing information for outcomes will be excluded from primary analysis. However, in sensitivity analysis, we will use multiple imputation by chained equations to replace missing outcome variables.

We will index the day of recruitment to be Day 0 and outcome assessment will take place on, or as close to possible after, Day 56.

Initiation of tuberculosis treatment will be defined by a participant in whom there is documented evidence of commencement of anti-tuberculosis treatment between Day 0 and up to, but not including Day 56, either by: inspection of the participant-carried national tuberculosis treatment card; or inspection of the facility tuberculosis treatment register; or inspection of TB treatment medication bottles or pill boxes.

Time to TB treatment outcome analysis will be right-censored at day 56 from randomisation if TB treatment is not initiated. We will estimate per-group median times to TB treatment initiation, and plot cumulative hazard function graphs.

To investigate the relative effectiveness of interventions on the cumulative hazard of TB treatment initiation, we will conduct log rank tests and construct Cox proportional hazard regression models to estimate hazard ratios and 95% confidence intervals for each pairwise comparison (e.g. Group 2 vs. Group 1, Group 3 vs. Group 2, and Group 3 vs. Group 1). Log-log plots will be examined and Schoenfeld residuals used to test the proportional hazards assumption.

To analyse binary secondary outcomes (proportion with same-day tuberculosis treatment initiation, proportion with undiagnosed/untreated pulmonary tuberculosis, proportion with undiagnosed/untreated HIV, proportion reported to have died by Day 56, proportion with successful TB treatment outcome), we will construct log-binomial regression models to estimate relative risk ratios and 95% confidence intervals, comparing between pairs of groups. We will additionally compare between pairs of groups the time to antiretroviral therapy initiation among participants with previously untreated HIV using Cox regression models.

To evaluate the effect of interventions on health-related quality of life, we will use analysis of covariance (ANCOVA) analysis to compare the mean EQ5D utility scores and visual analogue scale scores measured at Day 56 between pairs of groups, adjusting for participants’ corresponding values measured at Day 0.

## Planned sub-group analysis

For the preplanned subgroup analysis of the primary trial outcome we will construct Cox proportion hazard regression models including a term for either sex (male or female) or microbiological TB status (either microbiologically confirmed or clinically-diagnosed) to estimate hazard ratios and 95% confidence intervals. We will use the likelihood ratio test to look for interactions between covariates and the trial group term. These pre-specified exploratory analyses will be done for hypothesis-generating purposes and to support the case for future research, as our previous studies have shown that men fare considerable worse than women throughout the TB diagnostic and care pathways.

## Exploratory Bayesian analysis of primary trial outcome

Using within and between participant elicited probability distributions, we will construct stakeholder group-specific pooled prior probability distributions (known as a “community of priors”). Each prior will be converted to a log-hazard ratio scale and fitted to a normal distribution using a hierarchical random-effects model, with terms for stakeholder group, and a random-effects term to account for correlation of guesses within individuals. This will allow comparison between stakeholder groups of the similarity in support of opinions of effectiveness and of uncertainty.

Using Bayes’ theorem, we will combine elicited stakeholder group-specific log hazard ratio prior distributions with log-likelihood hazard ratio distributions from each pairwise comparison being made in the PROSPECT Study to construct posterior probability distributions. All analysis will be done in R and posterior mean hazard ratios and 95% credible intervals will be estimated by taking 4000 post-warmup draws from the posterior distributions using the No-U-Turn Sampler (NUTS) implemented with Stan.

It is anticipated that posterior distributions will demonstrate convergence between and within the sceptical and enthusiastic tails of stakeholder group prior distributions, indicating greater likelihood of accepting evidence. We will compare these findings with an analysis using a diffuse prior to investigate the impact of the prior specification on the posterior distribution. Finally, we will compare results obtained from the Bayesian analysis with that obtained from the main frequentist analysis.

# Economic evaluation

The main objective of the economic evaluation is to estimate the cost-effectiveness of the three interventions considered in the PROSPECT study. Specifically, we will estimate the incremental cost-effectiveness of the two optimized TB/HIV diagnosis and treatment linkage interventions in comparison to standard of care, and to each other. To achieve this, a systematic comparison of both the costs and consequences associated with the interventions will be conducted.

Two economic evaluations will be undertaken: firstly, a within trial evaluation; and secondly, a decision-analytic based cost effectiveness model. Both will be used to estimate the expected incremental cost per quality-adjusted life year (QALY) gained for the two optimized TB/HIV interventions in comparison to standard of care. For both analyses, the perspective will be that of the Malawi Ministry of Health and will only include the direct medical costs. The primary outcome measure will be the quality-adjusted life year (QALY). Health-related quality of life (HRQoL) will be estimated using responses to the EuroQol EQ-5D-3L obtained from participants. The within trial evaluation will adopt a time horizon matching the length of follow-up in the trial, and as the trial is following up patients for less than one year, there is no need to discount costs or QALYs. The model-based evaluation will adopt a lifetime horizon so as to incorporate the long-term costs and health consequences of delayed TB/HIV diagnosis and treatment initiation. For the model-based evaluation future costs and QALYs will be discounted at 3.5%, and sensitivity analysis will explore alternative discount rates.

As the trial is exploring the impact of the interventions on both TB and HIV diagnosis, the primary outcome for the health economic analysis will be QALYs. QALYs are a metric that unites quantity and health-related quality of life into a single metric. To do this, health-related quality of life is measured on an index scale where 1 equates to full health, and 0 equals death. These values are then combined with the time spent within each health state to generate QALYs. One QALY equates to one year in full health. To calculate QALYs it is imperative to obtain health state values for participants within the trial. To do this, the EQ-5D-3L measure has been included in the PROSPECT trial. The EQ-5D-3L is a generic HRQoL measure, and has been translated into Chichewa following international and EuroQoL guidelines. The EuroQol group has approved the final validated version. The EQ-5D measure consists of two principal measure components, a descriptive system and a visual analogue scale (VAS). The descriptive system defines HRQoL on the day of response in terms of five dimensions: ‘mobility’, ‘self care’, ‘usual activities’, ‘pain/discomfort’ and ‘anxiety/depression’. Responses in each dimension are divided into three ordinal levels, coded: (1) no problems; (2) some or moderate problems; and (3) severe or extreme problems. Responses to the three level version of the EQ-5D place respondents into one of 243 health states. Each response is then converted to an EQ-5D utility score using a tariff. Tariff sets have been derived from national surveys of the general population, with a subset of the 243 health states being valued, most commonly using the time trade-off method. Currently no Malawian EQ-5D tariff exists, and therefore the Zimbabwean EQ-5D tariff set will be used to derive EQ-5D utility scores. The Zimbabwean tariff results in EQ-5D utility scores ranging from 1.0 (no problems in the five dimensions) to -0·29 (severe problems in all five dimension). It is accepted practice to use tariffs from another country where none exists for the country of interest, provided the two populations value health comparably. The VAS, similar to a thermometer, ranges from 100 (best imaginable health state) to 0 (worst imaginable health state). Participants are asked to indicate how good or bad their health was on the day of response by drawing a line on the VAS.

In the trial, participants will complete the EQ-5D-3L at two time points. These measurements will occur at baseline and on Day 56. Health state values as measured by the EQ-5D-3L will be combined with time to calculate QALYs, this will be done by calculating the area under the curve. This method assumes that the health states reported at each time point are connected via a linear line between each time point. The trapezium rule is then used to calculate the area below this line (i.e. area under the curve). The area below this line are the QALYs associated with that health profile.

The primary analysis will focus on the direct intervention and the broader healthcare costs. The direct intervention costs are the costs associated with the application of the three interventions. This will include the costs of TB and HIV screening, the costs incurred in linking participants to TB/HIV services and any associated staff costs. The healthcare resources used will be captured prospectively and primary costing studies will be undertaken to estimate the unit costs for these identified resources. The broader healthcare resource use will be captured through a follow-up questionnaire at Day 56. This questionnaire will capture healthcare resources used since receiving the intervention, and will include:

- Outpatient clinic visits
- Days of inpatient hospital care
- Medications
- Investigations and procedures

As Malawi Ministry of Health does not have reference costs for these healthcare resources, previous unit costs estimated in Malawi will be used to derive total costs.

Cost-effectiveness results for the within trial analysis will be obtained by using methods appropriate for the trial data. Non-parametric bootstrap methods provide unbiased cost-effectiveness estimates only if baseline covariates (EQ-5D utility values) are balanced between the trial arms. Regression methods are justified when there is an imbalance of baseline EQ-5D utility values between the trial arms. Failure to account for such an imbalance will lead to biased cost-effectiveness estimates. As the distributions of costs and QALYs are commonly skewed, and often bimodal or truncated, a range of estimators will be explored, and model diagnostics will be undertaken to determine optimal choice. Mean costs and outcomes for each intervention will be estimated, together with the mean incremental cost-effectiveness ratio. Measures of uncertainty (standard errors and confidence intervals) will also be reported for the mean estimates. In addition, net monetary benefits (NMBs) will be estimated for a range of different willingness to pay (WTP) thresholds. Based on the NMB framework, cost-effectiveness acceptability curves (CEACs) will be constructed to identify the optimal intervention at different WTP thresholds.

The model-based evaluation will aim to extrapolate trial findings to allow estimation of cost-effectiveness over a lifetime time horizon. The model will likely consist of mutually exclusive Markov health states. These health states will be defined by a combination of untreated and treated states for both HIV and TB. The model will be parametrised by findings observed in the trial, and data extracted from the published literature.

Sensitivity and scenario analysis will also be considered. This will include undertaking a cost-effectiveness analysis where the primary outcome will reflect two outcomes being evaluated in the trial: the proportion of participants with undiagnosed or untreated TB; and the proportion of participants with undiagnosed untreated HIV. The aim of this analysis will be to estimate the:

- Incremental cost per additional individual started on TB treatment
- Incremental cost per additional individual started on ART.

As missing data is a common occurrence in trials, additional analysis will explore impact of missing data and alternative approaches to account for missing data

## Data handling and management

Data will be collected by research assistants using the mobile CommCare data collection platform running on fingerprint secured tablets. Data will be transmitted to the secure study server over encrypted cellular networks. The MLW Data Department has considerable experience in building robust electronic data collection surveys and in secure data management, backup and processing. A full audit trial of database changes will be maintained.

Building upon our extensive experience of conducting previous trials using electronic data collection systems in Blantyre, the trial statistician and Chief Investigator will write scripts within the statistical programme R that will interface with the trial database and, on a regular automated basis, use logical rules to identify records with missing or implausible values that will be hand-checked against source records to ensure completeness and validity of the final dataset.

We are strongly committed to ensuring that the trial datasets are made openly available, and that all code used in the analysis are published to allow fully reproducible research. The data collected by this research will be of importance to other researchers and the public, and could for example be used by other researchers conducting meta-analysis, or by policymakers modelling the potential return on investment of implementing interventions within their settings. Therefore, we will establish a public online GitHub repository, where the final anonymised individual- level trial dataset and code to allow reproduction of all analysis will be published. The availability of these resources will be publicised within academic manuscripts, through the MLW and LSTM websites.

# Laboratory methods

## Sputum GeneXpert MTB/Rif testing

Participants allocated to Group 3 will submit one sputum sample for GeneXpert MTB/Rif testing at baseline. Additionally, all participants assessed at the Day 56 outcome clinic visit will submit a sputum sample for GeneXpert MTB/Rif testing. Baseline sputum samples will be tested using the GeneXpert unit located at the study clinic. Day 56 outcome assessment sputum samples will be tested using the GeneXpert unit located at the College of Medicine of Malawi/MLW Research Tuberculosis Laboratory.

Research Assistants will provide instructions to participants to ensure production of sufficient volume and quality of sputa. Sputum specimens handled in the clinic are unlikely to pose a major hazard, due to natural ventilation and the low concentration of mycobacteria in unprocessed specimens. Nonetheless, safety training will be given to all employees and universal precautions (including the use of N95 masks) will be used to minimise the hazard of acquiring TB or other respiratory pathogens. Participants will be instructed to produce sputum samples in a designated, private, well-ventilated area outside the study research office.

## Sputum smear and TB culture testing

One sputum samples taken from each participant at the Day 56 outcome assessment visit will be transported in cool-boxes each day to the tuberculosis laboratory located at the College of Medicine of Malawi in Blantyre.

At the TB laboratory, trained and registered technicians will handle and process specimens. Sputum will be decontaminated with 4% sodium hydroxide for 15 minutes, neutralised with phosphate buffered saline, and concentrated using centrifugation at 3g for 15 minutes. Smears will be made from concentrated decontaminated (4% NaOH method) sputum, stained with Auramine-O and examined under fluorescence microscopy. All positive slides and one in ten negative slides will be reread by a second reader. The decontaminated pellet will be re-suspended in phosphate-buffered saline and aliquots inoculated onto a microscope slide, and into Mycobacterial Growth Indicator Tubes (MGIT) for culture, with the remaining aliquot stored at -20 degrees C.

In the case of contaminated cultures, the stored specimen will be retrieved and recultured after repeat decontamination. Cultures will be inspected weekly for up to 8 weeks. Species identification will use MBP 64 lateral flow assays, microscopic cording, and if either of these two are negative, colony morphology, temperature and inhibition by PNB.

Positive smear and culture results will be reported to participants within 3 days of confirmation, with home tracing if necessary. Participants will be supported to register for TB treatment through the National Tuberculosis Programme at the study clinic.

# Study administration and monitoring

## Approvals

The research has been granted ethical approval from the College of Medicine of Malawi Research Ethics Committee (COMREC), and from the Ethics Review Board of the Liverpool School of Tropical Medicine.

Additional approval has been granted from facility staff, and from Blantyre District Health Office.

## Trial Sponsor and Insurance

The Liverpool School of Tropical Medicine has agreed to act as the study sponsor, subject to satisfactory review of the research protocol, and appropriate permissions and ethical review being in place. The study will be covered by the Liverpool School of Tropical Medicine clinical trial insurance policy.

## Trial Steering Committee

We will establish a Trial Steering Committee comprising an independent chair, PI, co-investigators, statistician, clinician, independent experts (including from National TB Programme) and participant and community representatives. The Trial Steering Committee will meet prior to trial start and 6-monthly thereafter to monitor progress.

## Data, Safety and Monitoring Board

An independent Data, Safety and Monitoring Board will be established comprising three members (an experienced TB researcher, a TB clinician, and statistician with African clinical trials experience). The Data, Safety and Monitoring Board will meet prior to trial start, and subsequently 6-monthly, and will make recommendations to the TSC on recruitment rates, outcome accrual and adverse events.

# Ethical considerations and regulations

## Informed consent procedures

All prospective participants will be asked to provide informed consent to take part in the trial. After provision of oral information and a written participant information sheet (Appendix 1 Participant information forms) from Good Clinical Practice-trained Research Assistants, participants will be informed that participation is entirely voluntary, and can be withdrawn at any time. Individuals will be clearly informed that this study is a research study; the investigators do not know which interventions are "best", and consequently participants will be allocated to interventions at random to allow assessment of cost-effectiveness.

Prospective participants will be given sufficient opportunity to ask question and consider participation, including by discussion with family members if necessary.

Individuals who agree to participate in the trial will sign and date two copies of the informed consent form (Appendix 2 Participant consent forms); one copy will be retained by the study team as a source document, and the other will be given to the participant.

Individuals who are illiterate will be asked to provide a witnessed thumbprint to confirm their informed consent to participate. Witnesses will be an independent individual not involved with the study.

## Ethical issues and potential harms

There are a number of potential benefits for participants in the PROSPECT Study, including the potential to receive early diagnosis and treatment for HIV and TB. Nevertheless, we have carefully considered potential harms for participants, and will inform participants about potential risks.

A single chest x-ray typically delivers an average effective dose of 0.01mSv, comparable to 10 days of natural background radiation, and with less than one in a million chance of causing cancer. The potential benefits of chest x-ray as a TB diagnostic are then likely to outweigh this very small risk.

Generation or perpetration of stigma is a concern when HIV and TB testing and treatment interventions are being offered, although our previous trials and studies from elsewhere strongly suggest that stigmatization is relatively uncommon and rarely results in harm or adverse outcomes. We will take extensive actions to ensure any potential for stigmatization is remove through ensuring that HIV testing and TB screening activities are undertaken in private areas and confidentiality is maintained.

To minimise any potential misdiagnosis of HIV and TB, we will rigorously follow diagnostic algorithms established by WHO and recommended under Malawi National Guidelines.

## Potential constraints

The research team have a strong track record of undertaking large randomized trials of TB and HIV interventions in Blantyre that have directly changed practice and informed policy in Malawi and globally. We have carefully assessed the resources required, and are confident that we will be able to complete the trial within the allocated timeframe.

Nevertheless, we have carefully identified potential constraints to completing the trial, and have made efforts to mitigate against these.

To ensure that all technologies (digital fingerprint recording, GeneXpert MTB/Rif, digital chest x-ray, CAD4TB) are implemented within the required timescale, we have built in a 2-3-month piloting phase to allow systems to be installed and tested. To ensure that the sample size of participants is recruited within the allocated time, we have based sample size estimates on previous studies we have done in Blantyre health centers, and on a weekly basis, will review summary recruitment reports against targets to ensure that they are being met and to allow early intervention if not. Recruitment summary reports will be reviewed by the DSMB.

To ensure we achieve high levels of trial follow-up at the Day 56 outcome assessment (as we have done in previous studies), we will use the electronic ePALS geolocation system to facilitate home tracing of participants who do not return for their follow-up visit.

Finally, to ensure interventions are provided in accordance with the trial protocol, we will develop standard operating procedures to be followed by relevant staff. We will also undertake extensive quality assurance activities, including by doing exit-interviews with a random sample of trial participants to ensure they received correct interventions.

## Training provided

The trial Research Assistants and radiology technician will receive training in Good Clinical Practice through a course provided by the College of Medicine of Malawi. They will additional receive study-specific protocol training from the study Principal Investigator. All Research Assistants will be trained and certified in HIV testing and counselling.

Through the MLW Clinical Research Programme, study staff will have access to an ongoing programme of high-quality training in research methods and will attend research updates and summary meetings. Research Assistants will be encouraged to maintain and report study progress reports at the weekly trial management team meeting.

The Chief Investigator and study team will benefit from the world-leading expertise in tuberculosis and HIV available through collaborators at MLW (e.g. Prof Liz Corbett), and at the College of Medicine (Dr Marriott Nliwasa).

# Dissemination

## Dissemination

We will share study results with participants, community members and facility health workers at a public dissemination workshop to be held either at MLW or at the study clinic following completion of all study activities. This workshop will provide key stakeholders to gain first insight into the study results, and to ask questions about how the study findings will be further disseminated and acted upon.

Trial results will be shared with the Blantyre District Health Office, Malawi National Tuberculosis Programme and with the Malawi National HIV Programme. We will report findings at national, regional and international conferences, and will submit a manuscript reporting trial findings to a peer-reviewed journal specialising in public health, HIV and tuberculosis.

To facilitate reproducibility of analysis, an anonymised minimal final dataset and all code required to reproduce analysis will be published in the trial GitHub repository.

## Translating Research into Policy

Results of this research will be important in guiding national, regional and international health policy. WHO, policymakers and parliamentarians are currently grappling with how to improve access to TB and HIV diagnosis and treatment, including the role of chest x-ray. A key objective of this study is therefore to translate research findings into normative guidance in Malawi, in sub-Saharan Africa, and through WHO.

We recognise that early engagement with policymakers is essential to translate research into action. Therefore, we undertaken preliminary scoping activities to identify key stakeholders that we will work with, including Malawi Ministry of Health TB/HIV Technical Working Groups, the Malawi Network for Evidence-Informed Decision Making (EvIDeNt) which includes regional linkage through the African Institute for Development Policy (AFIDEP), and WHO TB-STAG.

# Study timeline

Estimates of recruitment rates are based upon our previously-published studies, and on surveillance data from Blantyre health centres. We estimate that approximately 2000 adults will attend the study clinic per month, of whom 10% have symptoms of tuberculosis. Assuming 50% of adults with TB symptoms are eligible and agree to participate in the study, recruitment will be completed over 15 months. Rates of clinic attendance, eligibility, recruitment, exclusions, and reasons for exclusion will be monitored on a weekly basis during the study.

Therefore, following an initial 6-month preparatory, piloting (4-6 weeks, from mid-April 2018) and training period, recruitment will commence in July 2018, and be completed in November 2019. Day 56 primary outcome assessments will be completed for all participants by December 2019, and data will be extracted from TB and ART clinic registers for a further 6-months for all participants who initiate treatment, meaning that all trial data collection will be completed by June 2020. Data cleaning, analysis (including of the nested diagnostic accuracy study) and dissemination will take place during July 2020 to July 2022.

**Study timeline**


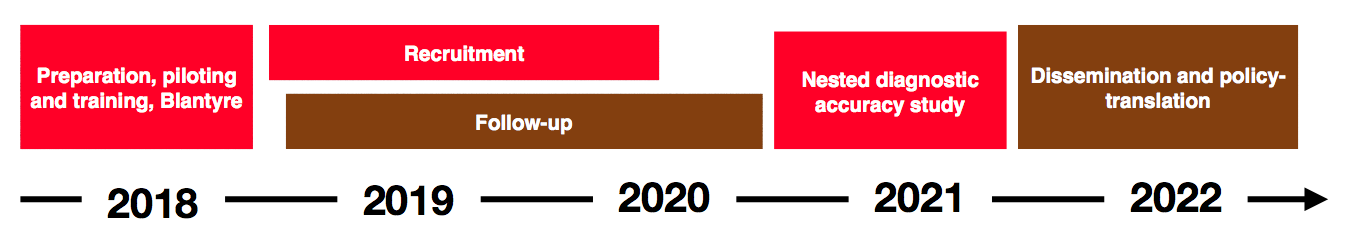


# Study budget

| **Budget line** | **LSTM (£)** | **MLW (£)** | **Total (£)** |
| --- | --- | --- | --- |
| **Salaries** |  |  |  |
| Research assistants x 6 |  | £53,020.00 | £53,020.00 |
| Radiography technician - TBC |  | £18,200.00 | £18,200.00 |
| **Subtotal** |  | **£71,220.00** | **£71,220.00** |
| **Materials and consumables** |  |  |  |
| OraSure OraQuick HIV 1/2 rapid diagnostic tests | £2,824.64 |  | £2,824.64 |
| Determine HIV 1/2 rapid HIV diagnostic tests |  | £214.59 | £214.59 |
| Sputum MGIT TB liquid culture (MLW lab) |  | £18,315.00 | £18,315.00 |
| Sputum TB smear microscopy |  | £2,442.00 | £2,442.00 |
| Sputum Xpert ULTRA MTB/Rif cartridges | £4,675.67 |  | £4,675.67 |
| CAD4TB chest x-ray software image processing | £1,965.60 |  | £1,965.60 |
| PACS digital chest x-ray cloud image storage and transfer | £1,225.00 |  | £1,225.00 |
| N95 masks for sputum collection |  | £750.00 | £750.00 |
| Cryoboxes for sputum transfer to lab | £135.25 |  | £135.25 |
| Nitrile gloves for HIV testing |  | £52.80 | £52.80 |
| Telephone communications (for participant tracing) |  | £600.00 | £600.00 |
| Participant reimbursement |  | £10,185.00 | £10,185.00 |
| Generator fuel for MinXray unit (2l/hr) |  | £1,200.00 | £1,200.00 |
| Shipping (WTCGHR) and clearing costs | £6,750.00 |  | £6,750.00 |
| Local transportation costs - (for tracing participants) |  | £550.00 | £550.00 |
| **Subtotal** | **£17,576.16** | **£27,034.39** | **£44,610.55** |
| **Equipment** |  |  | £- |
| Portable digital chest x-ray unit (MinXray, MinXray Inc, USA) | £37,184.00 |  | £37,184.00 |
| Portable diesel generator for MinXray unit |  | £2,200.00 | £2,200.00 |
| Xpert Unit | £1,667.00 |  | £1,667.00 |
| Laptop computer | £1,500.00 |  | £1,500.00 |
| Desktop computer for data processing/handling | £800.00 |  | £800.00 |
| Simprints fingerprint scanner and software | £480.00 |  | £480.00 |
| Android tablets and rugged cases for data collection | £1,800.00 |  | £1,800.00 |
| **Subtotal** | **£43,431.00** | **£2,200.00** | **£45,631.00** |
| **Miscellaneous** |  |  | £- |
| 1st International collaborator Pragmatic Clinical Trials workshops (Malawi), 20 people, room and equipment hire, refreshments |  | £4,880.00 | £4,880.00 |
| 2nd International collaborator Pragmatic Clinical Trials workshops (Liverpool), 20 people, room and equipment hire, refreshments | £6,745.00 |  | £6,745.00 |
| Good Clinical Practice training for research assistants |  | £1,260.00 | £1,260.00 |
| Radiology safety training (for technician) |  | £1,500.00 | £1,500.00 |
| Recruitment costs for research assistants |  | £200.00 | £200.00 |
| Research staff tee-shirts |  | £50.00 | £50.00 |
| IT internet access at MLW |  | £1,000.00 | £1,000.00 |
| Shipping insurance MinXray Unit | £100.00 |  | £100.00 |
| Translation costs (English-Chichewa-English) |  | £150.00 | £150.00 |
| Public engagement costs |  | £400.00 | £400.00 |
| Health and safety including post exposure prophylaxis for research assistants |  | £1,500.00 | £1,500.00 |
| LSTM trial sponsorship costs | £5,750.00 |  | £5,750.00 |
| COMREC Ethics application fee |  | £100.00 | £100.00 |
| COM direct research costs |  | £11,149.00 |  |
| **Subtotal** | **£12,595.00** | **£11,040.00** | **£23,635.00** |
| **GRAND TOTAL** | **£73,602.16** | **£111,494.39** | **£185,096.55** |

# References

1. World Health Organization. Global tuberculosis report 2017. Geneva, 2017.

2. The Global Burden of Disease Study Team. Global, regional, and national incidence and mortality for HIV, tuberculosis, and malaria during 1990-2013: a systematic analysis for the Global Burden of Disease Study 2013. *Lancet (London, England)* 2014; **384**(9947): 1005-70.

3. Corbett EL, Watt CJ, Walker N, et al. The growing burden of tuberculosis: global trends and interactions with the HIV epidemic. *Archives of internal medicine* 2003; **163**(9): 1009-21.

4. World Health Organization. END TB Strategy. Geneva, 2016.

5. Khan MS, Fletcher H, Coker R. Investments in tuberculosis research - what are the gaps? *BMC medicine* 2016; **14**(1): 123.

6. Harries AD, Lawn SD, Getahun H, Zachariah R, Havlir DV. HIV and tuberculosis--science and implementation to turn the tide and reduce deaths. *Journal of the International AIDS Society* 2012; **15**(2): 17396.

7. Gupta RK, Lucas SB, Fielding KL, Lawn SD. Prevalence of tuberculosis in post-mortem studies of HIV-infected adults and children in resource-limited settings: a systematic review and meta-analysis. *AIDS (London, England)* 2015; **29**(15): 1987-2002.

8. MacPherson P, Lalloo DG, Choko AT, et al. Suboptimal patterns of provider initiated HIV testing and counselling, antiretroviral therapy eligibility assessment and referral in primary health clinic attendees in Blantyre, Malawi. *Tropical medicine & international health : TM & IH* 2012; **17**(4): 507-17.

9. Peter JG, Zijenah LS, Chanda D, et al. Effect on mortality of point-of-care, urine-based lipoarabinomannan testing to guide tuberculosis treatment initiation in HIV-positive hospital inpatients: a pragmatic, parallel-group, multicountry, open-label, randomised controlled trial. *Lancet (London, England)* 2016; **387**(10024): 1187-97.

10. MacPherson P, Corbett EL, Makombe SD, et al. Determinants and consequences of failure of linkage to antiretroviral therapy at primary care level in Blantyre, Malawi: a prospective cohort study. *PloS one* 2012; **7**(9): e44794.

11. Macpherson P, Dimairo M, Bandason T, et al. Risk factors for mortality in smear-negative tuberculosis suspects: a cohort study in Harare, Zimbabwe. *The international journal of tuberculosis and lung disease : the official journal of the International Union against Tuberculosis and Lung Disease* 2011; **15**(10): 1390-6.

12. MacPherson P, Houben RM, Glynn JR, Corbett EL, Kranzer K. Pre-treatment loss to follow-up in tuberculosis patients in low- and lower-middle-income countries and high-burden countries: a systematic review and meta-analysis. *Bulletin of the World Health Organization* 2014; **92**(2): 126-38.

13. World Health Organization. Systematic screening for active tuberculosis: principles and recommendations. Geneva, 2013.

14. Cazabon D, Alsdurf H, Satyanarayana S, et al. Quality of tuberculosis care in high burden countries: the urgent need to address gaps in the care cascade. *International journal of infectious diseases : IJID : official publication of the International Society for Infectious Diseases* 2016.

15. Chihota VN, Ginindza S, McCarthy K, Grant AD, Churchyard G, Fielding K. Missed Opportunities for TB Investigation in Primary Care Clinics in South Africa: Experience from the XTEND Trial. *PloS one* 2015; **10**(9): e0138149.

16. Corbett EL, MacPherson P. Tuberculosis screening in high human immunodeficiency virus prevalence settings: turning promise into reality. *The international journal of tuberculosis and lung disease : the official journal of the International Union against Tuberculosis and Lung Disease* 2013; **17**(9): 1125-38.

17. Davis JL, Cattamanchi A, Cuevas LE, Hopewell PC, Steingart KR. Diagnostic accuracy of same-day microscopy versus standard microscopy for pulmonary tuberculosis: a systematic review and meta-analysis. *The Lancet Infectious diseases* 2013; **13**(2): 147-54.

18. Steingart KR, Henry M, Ng V, et al. Fluorescence versus conventional sputum smear microscopy for tuberculosis: a systematic review. *The Lancet Infectious diseases* 2006; **6**(9): 570-81.

19. Cruciani M, Scarparo C, Malena M, Bosco O, Serpelloni G, Mengoli C. Meta-analysis of BACTEC MGIT 960 and BACTEC 460 TB, with or without solid media, for detection of mycobacteria. *Journal of clinical microbiology* 2004; **42**(5): 2321-5.

20. Steingart KR, Schiller I, Horne DJ, Pai M, Boehme CC, Dendukuri N. Xpert(R) MTB/RIF assay for pulmonary tuberculosis and rifampicin resistance in adults. *The Cochrane database of systematic reviews* 2014; (1): Cd009593.

21. Hoog AH, Meme HK, van Deutekom H, et al. High sensitivity of chest radiograph reading by clinical officers in a tuberculosis prevalence survey. *The international journal of tuberculosis and lung disease : the official journal of the International Union against Tuberculosis and Lung Disease* 2011; **15**(10): 1308-14.

22. World Health Organization. Chest Radiology in Tuberculosis Detection: Summary of recommendation and guidance on programmatic approaches. Geneva, 2016.

23. Pande T, Cohen C, Pai M, Ahmad Khan F. Computer-aided detection of pulmonary tuberculosis on digital chest radiographs: a systematic review. *The international journal of tuberculosis and lung disease : the official journal of the International Union against Tuberculosis and Lung Disease* 2016; **20**(9): 1226-30.

24. Choko AT, MacPherson P, Webb EL, et al. Uptake, Accuracy, Safety, and Linkage into Care over Two Years of Promoting Annual Self-Testing for HIV in Blantyre, Malawi: A Community-Based Prospective Study. *PLoS medicine* 2015; **12**(9): e1001873.

25. Nliwasa M, MacPherson P, Mukaka M, et al. High mortality and prevalence of HIV and tuberculosis in adults with chronic cough in Malawi: a cohort study. *The international journal of tuberculosis and lung disease : the official journal of the International Union against Tuberculosis and Lung Disease* 2016; **20**(2): 202-10.

# Appendix 1: Participant information sheets

1. Participant information sheet (English) – Version 1.0: 2018_03_01

1. Participant information sheet (Chichewa) – Version 1.0: 2018_03_01

# Appendix 2: Participant consent forms

1. Participant Consent Form (English) – Version: V1.0: 2018_03_01

1. Participant consent form (Chichewa) – Version: 1.0: 2018_03_01

# Appendix 3: Case record forms

Attached:

1. Case record forms (English & Chichewa) – version 0.3: 2018-02-14

# Appendix 3: Statistical analysis plan

Attached:

1. Statistical analysis plan – version 1.0: 2018-03-13

# Appendix 4: Trial results tables

This Appendix shows figures and tables for key results that will be reported by the study.

**Figure 1: Trial profile**


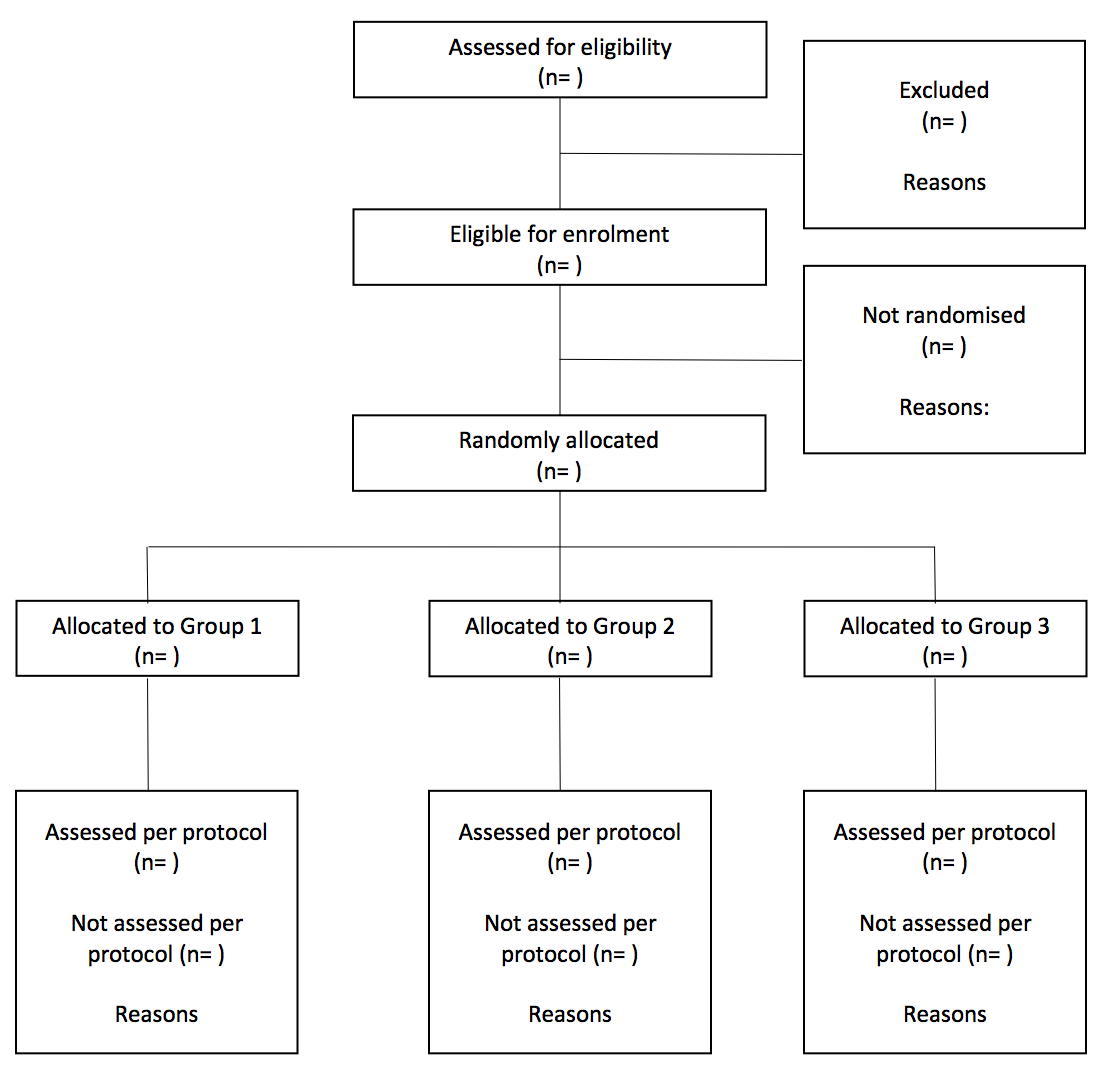


**Table 1: Baseline characteristics**

| **Characteristic** | **Group 1** | **Group 2** | **Group 3** |
| --- | --- | --- | --- |
| Age in years (mean, sd) |  |  |  |
| Sex |  |  |  |
| Male (n, %) |  |  |  |
| Female (n, %) |  |  |  |
| BMI (mean kg/m^2^, sd) |  |  |  |
| Marital status |  |  |  |
| Married/cohabiting (n, %) |  |  |  |
| Never married (n, %) |  |  |  |
| Widowed/separated/divorced (n, %) |  |  |  |
| Highest level of education |  |  |  |
| No schooling (n, %) |  |  |  |
| Primary (n, %) |  |  |  |
| Secondary no MSCE (n, %) |  |  |  |
| Secondary with MSCE (n, %) |  |  |  |
| Higher (n, %) |  |  |  |
| Tuberculosis symptoms |  |  |  |
| Cough (n %) |  |  |  |
| Duration of cough (median weeks, IQR) |  |  |  |
| Night sweats (n %) |  |  |  |
| Weight loss (n %) |  |  |  |
| Fever (n %) |  |  |  |
| Previously treated for TB (n %) |  |  |  |
| HIV status |  |  |  |
| HIV-positive (n %) |  |  |  |
| Taking ART (n %) |  |  |  |
| HIV-negative (n %) |  |  |  |
| Unknown (n %) |  |  |  |
| EQ5D utility score (mean, sd) |  |  |  |
| EQ5D visual analogue scale score (mean, sd) |  |  |  |

* MSCE: Malawi Secondary Certificate of Education

**Table 2: Primary outcome: Effectiveness of interventions on time to TB treatment initiation**

| **Outcome** | **Group 1** | **Group 2** | **Group 3** | **HR (95% CI): Group 2 vs. 1** | **HR (95% CI): Group 3 vs. 2** | **HR (95% CI): Group 3 vs. 1** |
| --- | --- | --- | --- | --- | --- | --- |
| Median (IQR) time to TB treatment initiation (days) |  |  |  |  |  |  |

* HR: Hazard ratio; CI: Confidence interval

**Table 3: Secondary outcome: Effectiveness of interventions on same day TB treatment initiation**

| **Outcome** | **Group 1** | **Group 2** | **Group 3** | **RR (95% CI): Group 2 vs. 1** | **RR (95% CI): Group 3 vs. 2** | **RR (95% CI): Group 3 vs. 1** |
| --- | --- | --- | --- | --- | --- | --- |
| Same-day TB treatment initiation (n/N %) |  |  |  |  |  |  |

* RR: Risk ratio; CI: Confidence interval

**Table 4: Secondary outcome: Effectiveness interventions on undiagnosed/untreated microbiologically-confirmed TB**

| **Endpoint** | **Group 1** | **Group 2** | **Group 3** | **RR (95% CI): Group 2 vs. 1** | **RR (95% CI): Group 3 vs. 2** | **RR (95% CI): Group 3 vs. 1** |
| --- | --- | --- | --- | --- | --- | --- |
| Undiagnosed or untreated microbiologically-confirmed pulmonary TB (n, %) |  |  |  |  |  |  |

* RR: Risk ratio; CI: Confidence interval

**Table 5: Secondary outcome: Effectiveness interventions on undiagnosed/untreated HIV**

| **Endpoint** | **Group 1** | **Group 2** | **Group 3** | **RR (95% CI): Group 2 vs. 1** | **RR (95% CI): Group 3 vs. 2** | **RR (95% CI): Group 3 vs. 1** |
| --- | --- | --- | --- | --- | --- | --- |
| Undiagnosed or untreated HIV (n, %) |  |  |  |  |  |  |

* RR: Risk ratio; CI: Confidence interval

**Table 6: Secondary outcome: All-cause mortality**

| **Endpoint** | **Group 1** | **Group 2** | **Group 3** | **RR (95% CI): Group 2 vs. 1** | **RR (95% CI): Group 3 vs. 2** | **RR (95% CI): Group 3 vs. 1** |
| --- | --- | --- | --- | --- | --- | --- |
| Deaths (n, %) |  |  |  |  |  |  |

* RR: Risk ratio; CI: Confidence interval

**Table 7: Secondary outcome: Proportion with successful TB treatment outcome 6-months post treatment initiation**

| **Endpoint** | **Group 1** | **Group 2** | **Group 3** | **RR (95% CI): Group 2 vs. 1** | **RR (95% CI): Group 3 vs. 2** | **RR (95% CI): Group 3 vs. 1** |
| --- | --- | --- | --- | --- | --- | --- |
| Successful TB treatment outcome (n/N, %) |  |  |  |  |  |  |

* RR: Risk ratio; CI: Confidence interval

**Table 8: Secondary outcome: Mean difference in EuroQoL EQ5D utility score**

| **Endpoint** | **Group 1** | **Group 2** | **Group 3** | **RR (95% CI): Group 2 vs. 1^†^** | **RR (95% CI): Group 3 vs. 2^†^** | **RR (95% CI): Group 3 vs. 1^†^** |
| --- | --- | --- | --- | --- | --- | --- |
| Mean EuroQoL EQ5D utility score at Day 56 (sd) |  |  |  |  |  |  |

* RR: Risk ratio; CI: Confidence interval

† Adjusted for baseline EuroQoL EQ5D utility score

**Table 9: Secondary outcome: Mean difference in EuroQoL EQ5D visual analogue scale score**

| **Endpoint** | **Group 1** | **Group 2** | **Group 3** | **RR (95% CI): Group 2 vs. 1^†^** | **RR (95% CI): Group 3 vs. 2^†^** | **RR (95% CI): Group 3 vs. 1^†^** |
| --- | --- | --- | --- | --- | --- | --- |
| Mean EuroQoL EQ5D visual analogue scale score at Day 56 (sd) |  |  |  |  |  |  |

* RR: Risk ratio; CI: Confidence interval

† Adjusted for baseline EuroQoL EQ5D visual analogue scale score

**Table 10 Pre-specified subgroup analysis: Time to TB treatment initiation by sex**

| Outcome | Group 1 | Group 2 | Group 3 | HR (95% CI): Group 2 vs. 1 | HR (95% CI): Group 3 vs. 2 | HR (95% CI): Group 3 vs. 1 |
| --- | --- | --- | --- | --- | --- | --- |
| *Men:* Median (IQR) time to TB treatment initiation (days) |  |  |  |  |  |  |
| *Women:* Median (IQR) time to TB treatment initiation (days) |  |  |  |  |  |  |

* HR: Hazard ratio; CI: Confidence interval

**Table 11 Pre-specified subgroup analysis: Time to TB treatment initiation by microbiological TB status**

| Endpoint | Group 1 | Group 2 | Group 3 | HR (95% CI): Group 2 vs. 1 | HR (95% CI): Group 3 vs. 2 | HR (95% CI): Group 3 vs. 1 |
| --- | --- | --- | --- | --- | --- | --- |
| *Microbiologically-confirmed pulmonary TB:* Median (IQR) time to TB treatment initiation (days) |  |  |  |  |  |  |
| *Clinically-diagnosed TB:* *Men:* Median (IQR) time to TB treatment initiation (days) |  |  |  |  |  |  |

* HR: Hazard ratio; CI: Confidence interval

# Appendix 5: Letters of Support

Attached:

1. Letter of support from the College of Medicine of Malawi


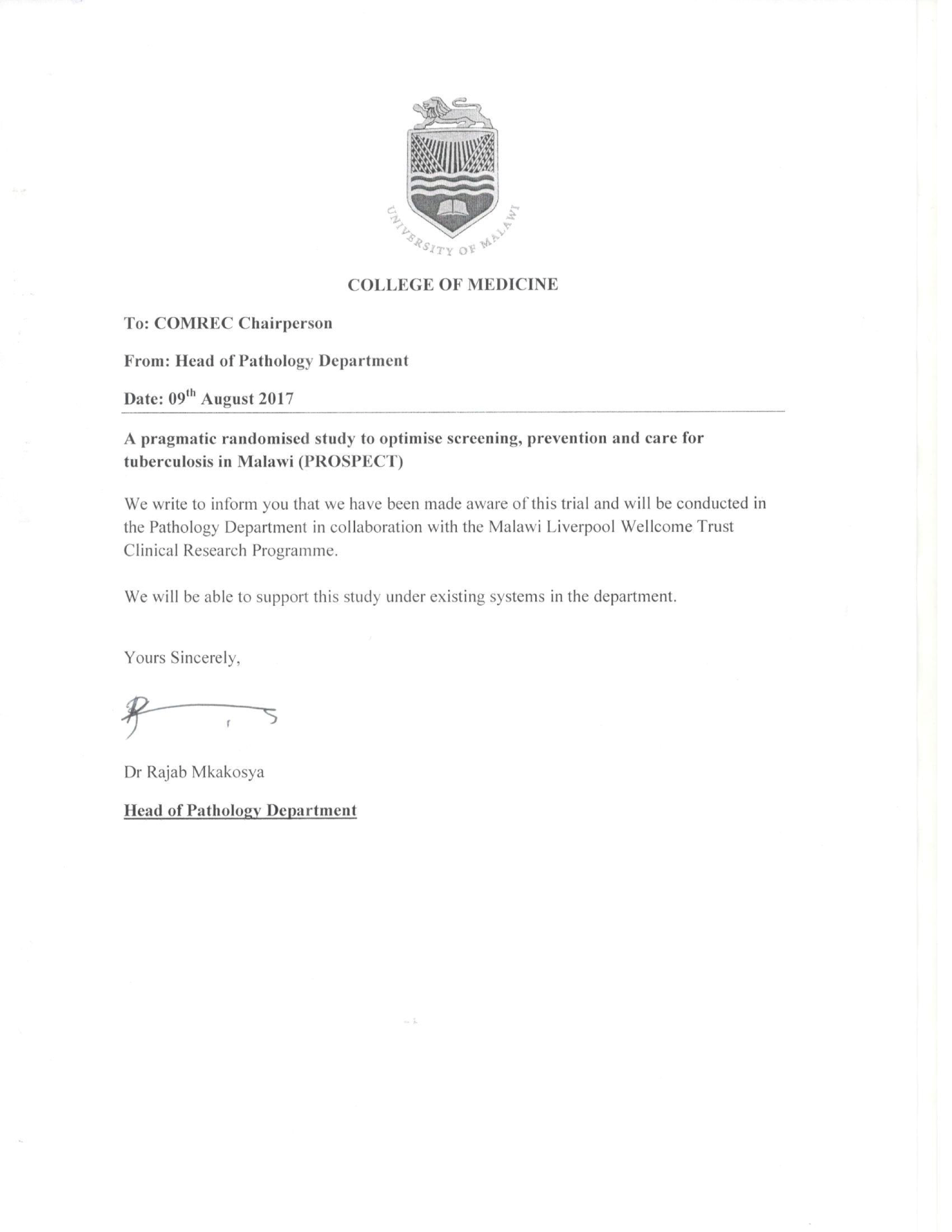


1. Letter of support from the Blantyre District Health Office


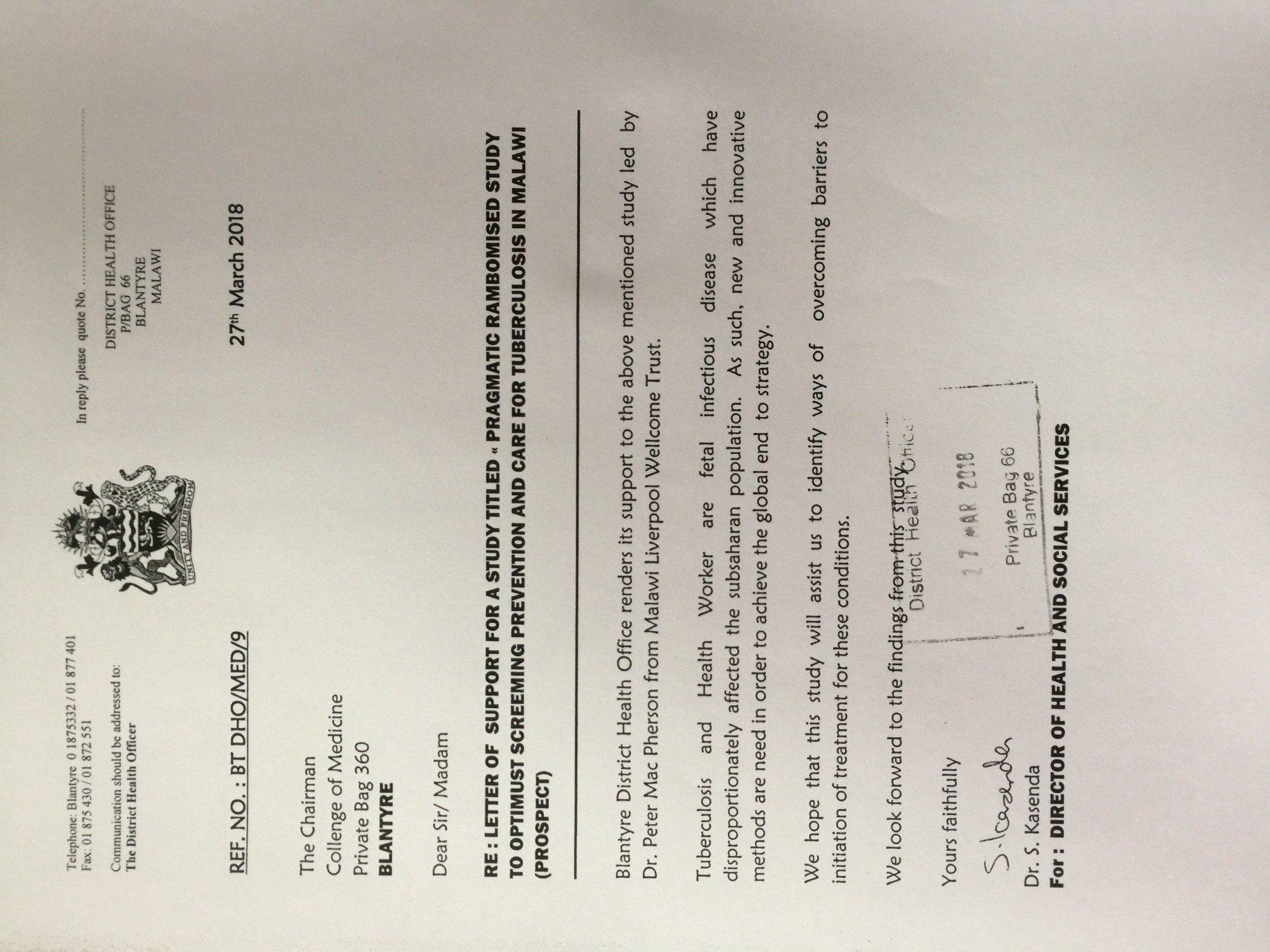

Supplement: S1 Text — (DOCX) [file pmed.1003752.s003.docx]
