# Supplementary material for: Computer-aided X-ray screening for tuberculosis and HIV testing among adults with cough in Malawi (the PROSPECT study): A randomised trial and cost-effectiveness analysis
Source: PLoS Med. 2021 Sep 9;18(9):e1003752. doi: 10.1371/journal.pmed.1003752 (PMC8459969; doi:10.1371/journal.pmed.1003752)
Supplement: S2 Text — (DOCX) [file pmed.1003752.s004.docx]

**The PROSPECT Study**

*A pragmatic randomised study to optimise prevention, screening and care for tuberculosis in Malawi*

**Document:** Statistical Analysis Plan

**Prepared by:** Peter MacPherson

Liverpool School of Tropical Medicine, UK &

Malawi-Liverpool-Wellcome Trust Clinical Research Programme

**Version:** 1.0: 2018-03-13

**Approved by:** Peter MacPherson
 Chief Investigator

**Approved on:** 2018-03-13

**Signature:**

**
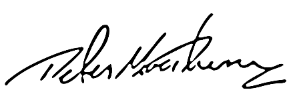
**

---------------------------------------------------------------------------------------------

Table of Contents

[Investigators 3](#_Toc504134855)

[Background to the PROSPECT Study 4](#_Toc504134856)

[Objectives 6](#_Toc504134857)

[Randomisation and blinding 6](#_Toc504134858)

[Description of interventions 7](#_Toc504134859)

[Outcomes 9](#_Toc504134860)

[Sample size justification 10](#_Toc504134861)

[Recruitment and trial profile 12](#_Toc504134862)

[Baseline enrolment characteristics 12](#_Toc504134863)

[Missing data 12](#_Toc504134864)

[Statistical analysis of primary outcome 13](#_Toc504134865)

[Statistical analysis of secondary outcomes 13](#_Toc504134866)

[Appendix 15](#_Toc504134867)

# **Investigators**

| Investigator | Institute | Role |
| --- | --- | --- |
| Dr Peter MacPherson | Malawi-Liverpool-Wellcome Trust Clinical Research Programme, Malawi & Liverpool School of Tropical Medicine, UK | Chief Investigator |
| Prof Liz Corbett | Malawi-Liverpool-Wellcome Trust Clinical Research Programme, Malawi, College of Medicine of Malawi, and London School of Hygiene and Tropical Medicine, UK | Co-investigator |
| Dr Emily Webb | London School of Hygiene and Tropical Medicine, UK | Statistician |
| Dr Liz Joekes | University of Liverpool, UK | Radiologist |
| Prof Madhukar Pai | McGill University, Canada | Co-Investigator |
| Dr Marriott Nliwasa | Malawi-Liverpool-Wellcome Trust Programme, Malawi and College of Medicine of Malawi | Trial Physician |
| Dr Hendramoorthy Maheswaran | University of Liverpool, UK | Health Economist |
| Prof Bertie Squire | Liverpool School of Tropical Medicine, UK | Co-Investigator |
| Prof David Lalloo | Liverpool School of Tropical Medicine, UK | Co-Investigator |

# **Background to the PROSPECT Study**

The PROSPECT Study is a pragmatic open, three-arm individually-randomised controlled trial and economic evaluation, which will be conducted in one primary health care centre in Blantyre, Malawi, where HIV and TB are major contributors to early mortality.

Participants will be adults (18 years or older) with symptoms of tuberculosis (any of: cough, fever, weight-loss, night sweats) attending the primary clinic with an acute care episode. We will exclude adults who have taken treatment for TB within the previous 6-months, who are taking isoniazid preventive therapy, or who plan to move outside of Blantyre.

Eligible participants who provide informed consent to participate will be randomly allocated into one of three groups:

**Group 1: Standard of care:** Participants will be seen by facility health workers and receive clinician-directed screening for HIV and TB according to Malawi national guidelines without further study input.

**Group 2: Optimised HIV testing and treatment linkage:** Participants will be offered testing for HIV using rapid oral fluid kits by research assistants. Those with confirmed HIV infection will be linked to the HIV care clinic where facility healthworkers will screen for TB using standard sputum-based diagnostics without further study input.

**Group 3: Optimised TB diagnosis, HIV screening and treatment linkage:** Participants will receive a high-throughput and high-sensitivity TB screening intervention, in addition to the HIV testing intervention. This will comprise of an initial digital chest x-ray classified by the CAD4TB image-recognition software as either "high probability of TB", or "low probability of TB". Participants whose x-rays are classified as having high probability of TB will receive confirmatory sputum testing with Xpert MTB/Rif Ultra cartridges, whilst participants whose x-rays have a low probability of TB will be referred to facility healthworkers for routine care.

All participants will be seen at the health facility at day 56, where they will be assessed to determine whether they are taking treatment for tuberculosis by inspection of medication, inspection of treatment cards, and inspection of facility TB registers. They will also be offered testing for HIV (if not on ART) and screened for TB, including by sputum culture, Xpert and smear microscopy.

The Primary Trial Outcome will compare between pairs of groups the time to tuberculosis treatment initiation up to but not including Day 56. The trial is sufficiently powered to permit three pairwise comparisons between groups (i.e. Group 1 vs. 2; Group 2 vs. 3; and Group 1 vs. 3).

This three-arm pragmatic trial design allows us to efficiently answer two separate, important public health questions: firstly, by comparing Group 2 to Group 1, we should be able to determine whether HIV care should be prioritised for adults with TB symptoms. Additionally, by comparing Group 3 to Group 2, we will provide evidence for the effectiveness of an optimised and integrated HIV and TB diagnostic and treatment linkage approach.

Figure 1 below outlines the design and delivery of interventions within the PROSPECT Study.

**Figure 1: Study design**


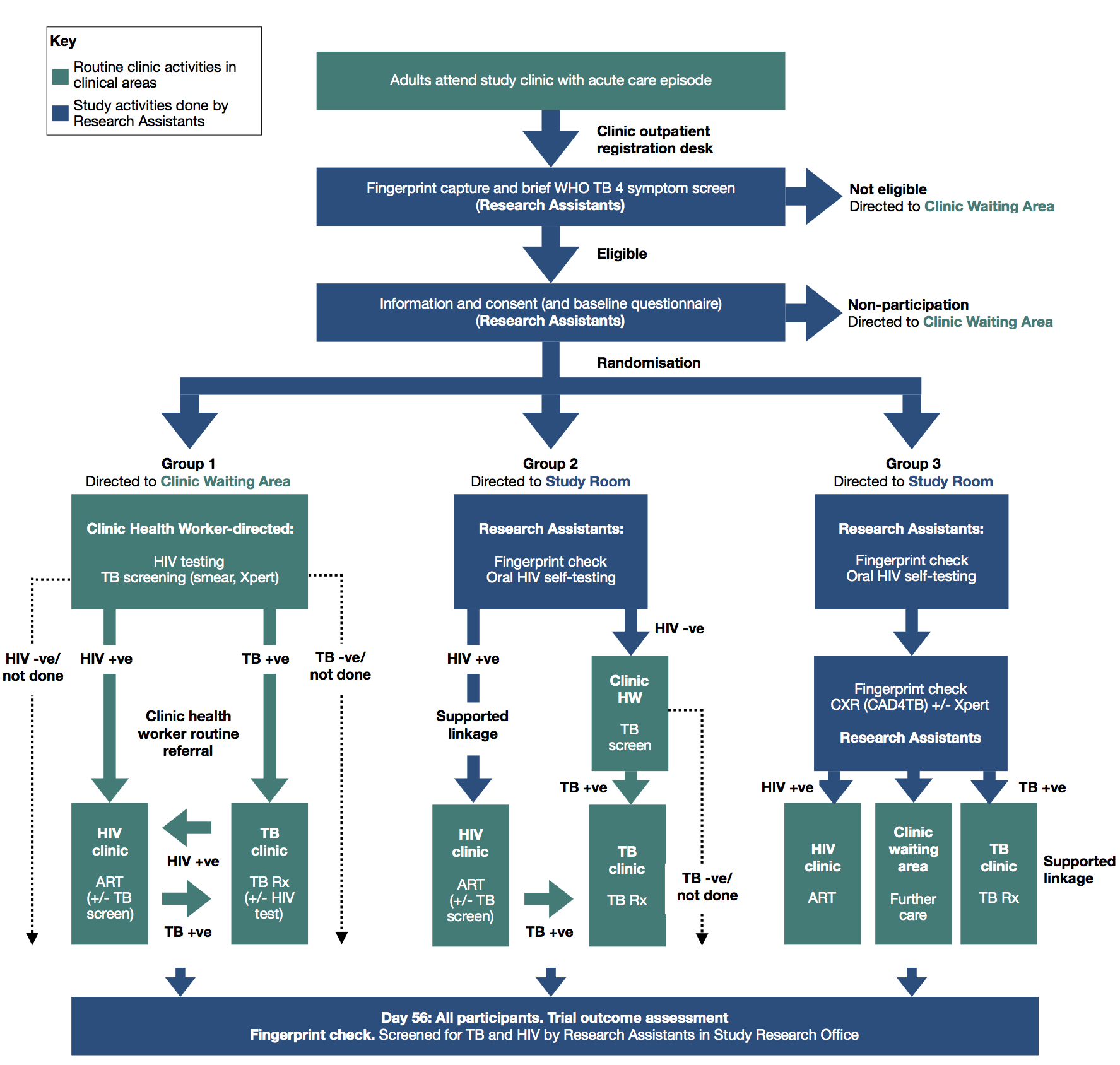


# **Objectives**

The overall aim of the PROSPECT Study is to investigate the effectiveness and cost-effectiveness of optimised TB/HIV diagnosis and treatment linkage interventions on TB and HIV case detection, treatment initiation and mortality.

The objectives of the PROSPECT Study are to:

1. Among adults with TB symptoms attending primary care in Malawi, investigate the effectiveness of an optimised same-day screening algorithm consisting of rapid HIV testing, computer-assisted CAD4TB chest x-ray triage and, if abnormal, Xpert MTB/Rif Ultra rapid sputum molecular testing, and linkage to treatment.
2. In a nested diagnostic accuracy study evaluate the sensitivity and specificity of computer-assisted chest x-ray triage compared to classification by radiologists and bacteriological diagnosis.
3. Undertake a cost-utility analysis of the PROSPECT interventions to estimate the incremental cost per QALY gained from providing optimised TB and HIV diagnosis and linkage to care.

This statistical analysis plan describes the analyses to be conducted for Objective 1; Objectives 2 and 3 will be assessed separately.

# **Randomisation and blinding**

Eligible participants who provide consent to take part in the PROSPECT Study) will be individually-randomly allocated in a 1:1:1 ratio into one of three groups

Randomisation will be done by Research Assistants immediately following recruitment (hereafter referred to as Day 0) and using a random number computer programme running on study data-collection electronic tablets. To remove any potential for allocation bias, the participant's unique identifying number, randomisation code, time of randomisation and demographic characteristics will be automatically recorded and uploaded to the trial database by cellular network, facilitating full reproducibility and audit of the randomisation and allocation process.

Using pre-written computer programmes, and without unblinding group allocation, the trial statistician and Chief Investigator will review on a weekly basis the numbers of participants allocated to each group to facilitate early identification of any issues with randomisation and allocation.

Because of the nature of the study and the interventions offered, it will not be possible to blind participants or research assistants to allocation groups. Nevertheless, extensive steps will be taken to ensure that Research Assistants undertaking day 56 outcome assessments are blinded to participants' group. Additionally, the investigators, including the Chief Investigator and Trial Statistician will remain blinded to allocation groups until database lock preceding final analysis. No unblinded interim analysis will be conducted.

# **Description of interventions**

*All participants*

All participants who provide written (or witnessed thumbprint) informed consent to participate in the study will complete a baseline questionnaire on Day 0, that will record demographic and clinical characteristics (including previous HIV and TB care), as well as geolocation information to facilitate home tracing using the ePALs geolocation system. All participants will additionally complete the EQ5D (Chichewa) tool to measure health-related quality of life.

Participants will be randomly allocated into one of three groups. Interventions provided to participants in each of the three trial groups are described below.

*Group 1: Standard of care*

In Group 1 (**standard of care),** participants will be directed to wait in the clinic waiting area to be seen by facility health workers. The facility health workers will direct all further investigations and care **without any further input from the study team**. Facility health workers at the clinic will have access to the following services through the National HIV and TB programmes:

- HIV testing and counselling, provided by Facility HIV Testers in the clinic HIV and testing room, who follow a serial rapid fingerprick diagnostic kit-based algorithm recommended by WHO (see Protocol for full HIV testing and counselling algorithm details). Note that WHO and Malawian guidelines based on expert technical review recommend that only very brief pre-test HIV counselling is required.
- Routine TB screening, with both sputum smear microscopy and Xpert MTB/Rif testing available onsite at the clinic.
- Routine linkage to the onsite HIV clinic, where patients are registered and assessed for initiation onto antiretroviral therapy by facility HIV Care Clinic health workers. Malawi guidelines recommend universal treatment for HIV. HIV Care Clinic healthworkers will additionally have access to TB screening tests as described above; Malawi and WHO guidelines recommend that all HIV-positive individuals are assessed for TB on every healthcare encounter.
- Routine linkage to the onsite TB clinic, where patients are registered and initiated onto tuberculosis treatment. Malawi guidelines recommend universal HIV testing for all patients with confirmed TB.

*Group 2: Optimised HIV testing and treatment linkage*

In Group 2 participants will be directed to the study room located in a separate building close to the main clinic building. Here, Study Research Assistants will validate the participant’s identity and trial allocation group by repeating the fingerprint scan that will validate identity and allocation group, and will offer a supervised HIV self-testing intervention, based on a model we have developed and implemented widely in Malawi and sub-Saharan Africa. Participants will be given brief pre-test instructions using previous developed materials, and will be asked to self-test in a private area using the OraQuick 1/2 (OraSure Technologies) oral fluid HIV kit. WHO strongly recommends against detailed pre-test HIV counseling, except in exceptional circumstances. Participants will be supported to read their HIV test result by study Research Assistants and, if they have a reactive OraQuick result, will be provided with confirmatory fingerprick testing by the trained Research Assistants following serial algorithm recommended by WHO and the Malawi National HIV Programme – see Protocol for full algorithm details. Following confirmatory testing,

- HIV-positive participants will be supported by Research Assistants to register at the onsite HIV care clinic, and all further care (including TB screening) will be directed by facility health workers **without any further study input**.
- HIV-negative participants will be referred to the clinic waiting area (with a copy of their HIV test results) to be seen by the facility health workers who will direct all further investigations (including TB screening) and care **without further study input**.

*Group 3: Optimised TB diagnosis, HIV screening and treatment linkage*

Group 3 participants will be directed to the Study Room and will have their identity and trial allocation group validated by fingerprint scanning. They will be offered the HIV self-testing and linkage intervention as described above for Group 2.

Additionally, they will be offered a TB screening intervention comprising of:

- A digital chest x-ray performed by the study radiographer in the study room, and using the study MinXray unit.
- Chest x-rays will be immediately classified by the CAD4TB image-classification software running on the MinXray unit laptop as either “high probability of TB”, or “low probability of TB”.
- Participants whose chest x-rays indicate a low probability of TB will be referred to facility health workers (at either the onsite HIV care clinic if HIV-positive, or the clinic waiting area), with copies of their results for further routine care. All further investigations and care will be directed by facility health workers without further study input
- Participants whose chest x-ray suggests a high probability of TB will submit a single spot sputum sample for testing using the Xpert MTB/Rifs Ultra cartridges. This will be done by trained Research Assistants within the clinic TB laboratory.

*Outcome assessment: all participants*

All participants will be seen at the health facility at Day 56, where following fingerprint validation, they will be able to reimburse a transport voucher. Attempts will be made to contact participants who do not attend their scheduled clinic appointment at home on up to three occasions. Tracing will be facilitated by use of our ePALS patient geolocation system.

At the Day 56 appointment, all participants will undergo a detailed questionnaire and check of any TB and HIV treatment cards and medication bottles to ascertain treatment status. TB and HIV treatment status will be validated against the Blantyre Enhanced Electronic Tuberculosis Surveillance System, where the details of all registering TB patients are routinely recorded by TB Officers.

All participants will be offered screening for tuberculosis by submitting two sputum samples, which will be tested by smear, culture and Xpert MTB/Rif Ultra at the College of Medicine tuberculosis research laboratory. All participants will additionally receive a digital chest x-ray, classified by CAD4TB, and subsequently read by a panel of seven radiologists as part of the nested diagnostic accuracy study (see protocol for full details of evaluation). Participants who have microbiologically-confirmed tuberculosis, or whose x-rays require further clinical evaluation will be traced and linked to care, either at the primary clinic, or at the respiratory clinic at Queen Elizabeth Central Hospital through the Trial Physician.

All participants confirmed to not be taking antiretroviral therapy will be offered testing for HIV done by the Research Assistants and using the serial testing algorithm described above and in the Protocol. Participants with confirmed HIV infection will be supported to register for further care and treatment at the facility HIV care clinic.

To estimate TB treatment outcomes at 6-months, we will use prospectively-collected TB treatment cohort data collected for the Blantyre Enhanced TB Surveillance Programme. On a quarterly basis TB treatment registers from all TB treatment centres in Blantyre are captured electronically and reconciled. In the PROSPECT Study, when a participant initiates TB treatment, we will record their national TB treatment number in the study database, and use this unique identifier to link participants to their TB treatment outcome data.

# **Outcomes**

*Primary outcome*

The primary trial outcome will compare between pairs of groups the time in days to tuberculosis treatment initiation, evaluated at Day 56 following randomisation.

Analysis of the primary outcome will be done on an intention to treat basis, with all participants analysed according to the group to which they were randomised. Time to TB treatment outcome analysis will be right censored at Day 56 from randomisation if TB treatment is not initiated, or at day of loss to follow-up (i.e. Day 0). We will make three pair-wise comparisons *(Group 2 vs. Group 1; Group 3 vs. Group 2; and Group 3 vs. Group 1)*.

This primary endpoint has been chosen because reducing time to initiation of treatment could have important individual and public health benefits. Assessment over eight weeks has been selected because: (i) TB culture is typically completed within 8 weeks, (ii) mortality is highest during this period, and (iii) previous trials and our pilot data show that TB treatment initiations plateau by 8 weeks.

*Secondary outcomes*

The secondary trial outcomes will compare between pairs of groups:

- The proportion of randomised participants initiated onto tuberculosis treatment on the same day as randomisation, with the numerator being participants who were initiated on tuberculosis treatment on Day 0, and the denominator being all randomised participants.
- The proportion of randomised participants with undiagnosed/untreated microbiologically-confirmed pulmonary TB at Day 56, with the numerator being participants with microbiologically-confirmed tuberculosis (either sputum culture, or sputum Xpert, or sputum smear microscopy positive on a sample taken on Day 56) and who are confirmed not to be taking tuberculosis treatment on Day 56 (including participants who have previously initiated tuberculosis treatment, but have defaulted or stopped treatment – regardless of reason – for at least one week). The denominator will be all randomised participants.
- The proportion of randomised participants with undiagnosed/untreated HIV at Day 56, with the numerator being participants with positive confirmatory HIV test results at Day 56 and who are not taking antiretroviral therapy (regardless of previous HIV test results during or before the study period), and the denominator being all randomised participants.
- Time in days - from Day 0 up to but not including Day 56 - to initiation of antiretroviral therapy among participants with positive confirmatory HIV test results at Day 56 and who were not taking antiretroviral therapy at Day 0.
- The proportion of randomised participants reported to have died by Day 56, with the numerator being participants confirmed to have died through home tracing visits or TB treatment records, and the denominator being all randomised participants
- The proportion of TB cases with a successful TB treatment outcome. The numerator will be participants who were initiated onto tuberculosis treatment (either microbiologically-confirmed or clinically-diagnosed tuberculosis) up to, but not including Day 56, and who have a successful TB treatment outcome (either cured or completed treatment) at 6-months after starting treatment. The denominator will be all participants confirmed to have initiated tuberculosis treatment between Day 0 and up to, but not including Day 56.
- Mean difference in EuroQoL EQ5D utility score at Day 56, adjusting for participants’ EQ5D utility score measured at Day 0.
- Mean difference in EuroQoL EQ5D visual analogue scale score, adjusting for participants’ EQ5D visual analogue scale score measured at Day 0.
- Incremental cost-effectiveness per QALY gained (see Economic Analysis Protocol for full details of measurement and analysis).

*Subgroup analysis*

In pre-planned exploratory analysis, we will stratify analysis of the primary trial outcome by:

1. Sex (male vs. female). In this analysis, we will compare between pairs of groups the time to tuberculosis treatment for male and female participants.
2. Microbiological status (microbiologically-confirmed TB vs. clinically-diagnosed TB). In this analysis, we will compare between pairs of groups the time to tuberculosis treatment for participants with microbiologically-confirmed and clinically-diagnosed TB.

*Exploratory Bayesian analysis*

Additionally, we will undertake a Bayesian analysis of the primary trial outcome. Prior distributions for the proportion of participants initiating TB treatment will be elicited from key stakeholder groups. We anticipate that key stakeholders will include:

**Table 1: Key stakeholders identified for Bayesian prior elicitation exercise**

| Stakeholder group | How identified |
| --- | --- |
| Community members/trial participants | Through MLW Community Engagement Officer |
| Clinicians | Through existing links with DHO and health centre/hospital clinicians |
| Researchers | Through MLW Research Group Leads |
| Experts (TB/HIV) | Through Trial Investigators’ contacts |
| Policymakers (Malawi, Regional, International) | Through Trial Investigators’ existing links with individuals and policy groups |

Key stakeholders will be invited to attend workshop meetings, where they will be introduced to the study design and interventions through presentations and group discussions. To elicit prior beliefs for effect of interventions, we will use a “bin-and-chip” method, implemented within an interactive web application:

(<https://pmacp.shinyapps.io/bayesian_prior_elicit_prospect/>).

Before eliciting stakeholders’ prior beliefs for trial interventions, we will provide a series of “warm-up” vignettes based around familiar events such as the probability of a football team winning, or the probability of it raining tomorrow. This will allow stakeholders to become familiar with the process of assigning probability to future events, and with completing the interactive web application.

Each stakeholder will then be asked to make ten guesses for the proportion of participants starting TB treatment using the interactive web application. Responses will be saved directly into a dedicated study database.

# **Sample size justification**

The primary trial outcome will compare between pairs of groups the time to tuberculosis treatment initiation among all randomised participants. To evaluate the relative effects of the HIV and TB screening/linkage interventions, we wish to make *k=3* pairwise comparison *(Group 2 vs. Group 1; Group 3 vs Group 2; and Group 3 vs Group 1)*.

Our pilot data show that 17% of 190 adults with TB symptoms initiated TB treatment under standard of care conditions within 56 days.

First, we estimate the sample size required for 80% power to detect various hazard ratios at 5% significance level, comparing Group 2 vs. Group 1. In sample size calculations, we use a formula for the proportional hazards model developed by Schoenfield. We assume 17% of participants in Group 1 will initiate TB treatment, as per pilot data.

**Table 2: Sample size estimates, comparing Group 2 vs. Group 1**

| **Cumulative hazard of TB treatment (Group 1)** | **Hazard ratio (Group 2 vs. 1)** | **Total number of events** | **Total N**  **(Groups 2 & 1)** |
| --- | --- | --- | --- |
| 0.17 | 1.30 | 456.10 | 2369 |
| 0.17 | 1.35 | 348.60 | 1777 |
| 0.17 | 1.40 | 277.31 | 1388 |
| 0.17 | 1.45 | 227.41 | 1119 |
| 0.17 | 1.50 | 190.97 | 923 |
| 0.17 | 1.55 | 163.46 | 777 |
| 0.17 | 1.60 | 142.12 | 665 |

**Note:** ^a^ Using formula for the proportional hazards model developed by Schoenfield

Now we estimate the sample size required for 80% power to detect various hazard ratios at 5% significance level, comparing Group 3 vs. Group 2.

**Table 6 Sample size estimates, comparing Group 3 vs. Group 2**

| **Cumulative hazard of TB treatment (Group 2)** | **Hazard ratio (Group 3 vs. 2)** | **Total number of events** | **N (Groups 3 & 2)** |
| --- | --- | --- | --- |
| 0.255 | 1.39 | 289.517 | 981 |
| 0.255 | 1.40 | 277.312 | 936 |
| 0.255 | 1.41 | 265.942 | 895 |
| 0.255 | 1.42 | 255.331 | 856 |
| 0.255 | 1.43 | 245.410 | 820 |

**Note:** ^a^ Using formula for the proportional hazards model developed by Schoenfield. ^b^ Assuming hazard ratio of 1.5 comparing Group 2 vs. Group 1.

Finally, assuming participants are allocated in a 1:1:1 ratio across the three groups, and inflating by 5% to account for loss, we estimate the overall sample size required.

**Table 7 Sample size estimates for pairwise comparisons**

| **Baseline hazard (Group 1)** | **Hazard ratio detectable (Group 2 v 1)** | **Sample size (Groups 1 & 2)** | **Baseline hazard (Group 2)** | **Hazard ratio detectable (Group 3 v 2)** | **Sample size (Groups 2 & 3)** | **Allocated in 1:1:1 ratio across three groups, and with 5% loss to follow-up** |
| --- | --- | --- | --- | --- | --- | --- |
| 17% | 1.40 | 1388 | 25.5% | 1.39 | 981 | 2187 |
| 17% | 1.45 | 1119 | 25.5% | 1.40 | 936 | 1763 |
| 17% | 1.50 | 923 | 25.5% | 1.41 | 895 | 1455 |
| 17% | 1.55 | 777 | 25.5% | 1.42 | 856 | 1224 |
| 17% | 1.60 | 665 | 25.5% | 1.43 | 820 | 1049 |

1 With power=80% and loss to follow-up of 5%. Assuming Group 2 vs. Group 1 HR=1.50

Therefore, adjusting for 5% loss to follow-up, a total sample size of 1455 participants (485 per group, = 923/2 x 3 x 1.05) gives at least 80% power to detect at least a cumulative hazard ratio for TB treatment initiation of 1.5 comparing Group 2 to Group 1, and a hazard ratio of 1.41 comparing Group 3 to Group 2. Additionally, under these assumptions, 485 participants per group would give 80% power to detect a hazard ratio of at least 1.50 comparing Group 3 to Group 1.

# **Recruitment and trial profile**

A trial profile figure (annex- Figure 1) will be produced illustrating the following:

1. Number of participants screened for eligibility, recruited and randomised
2. Number of participants randomly-allocated to each group
3. Number of participants who received allocated interventions, by group
4. Number of participants assessed at Day 56 outcome assessment, by group
5. Number of participants lost to follow-up, by group
6. Number of participants with data that are not analysable, and reasons (i.e. missing diagnosis, key data missing), by group
7. Number of participants analysed for each trial outcome, by group

# **Baseline enrolment characteristics**

Baseline enrolment characteristics will be examined in each trial arm (Annex- Table 1), and will include:

- Age in years
- Sex
- Body mass index (in kg/m^2^)
- Marital status
- Level of education
- Tuberculosis symptoms (cough, cough duration, fever, weight loss, night sweats)
- Previous TB treatment
- HIV status and antiretroviral therapy treatment
- EuroQoL EQ5D utility score
- EuroQoL EQ5D visual analogue scale score (from 0-100)

# **Missing data**

Data will be collected using an electronic data collection application, and we will use logical rules and skip patterns to minimise missing data.

Missing data will be examined for each variable and for each individual participant. A systematic assessment of missingness will be conducted to ascertain the reason and possible mechanism for missing data by identifying the quantity of missing data and patterns within the data. Missingness will be compared between randomised arms to assess for systematic biases.

Participants who have missing information for outcomes will be excluded from primary analysis. However, in sensitivity analysis, we will use multiple imputation by chained equations to replace missing outcome variables.

# **Statistical analysis of primary outcome**

Trial reporting will follow CONSORT Guidelines. Analysis of the primary and secondary outcomes will be done on an intention to treat basis, with all participants allocated to trial groups included in the denominator. We do not anticipate imbalance between groups, so unadjusted effect estimates will be reported.

We will index the day of recruitment to be Day 0 and outcome assessment will take place on, or as close to possible after, Day 56.

Initiation of tuberculosis treatment will be defined by a participant in whom there is documented evidence of commencement of anti-tuberculosis treatment between Day 1 and up to, but not including Day 56, either by: inspection of the participant-carried national tuberculosis treatment card; or inspection of the facility tuberculosis treatment register; or inspection of TB treatment medication bottles or pill boxes.

Time in days to TB treatment outcome analysis will be right-censored at day 56 from randomisation if TB treatment is not initiated, or earlier if the participant is lost to follow-up.

We will estimate per-group median times to TB treatment initiation, and plot cumulative hazard function graphs.

To investigate the relative effectiveness of interventions on the cumulative hazard of TB treatment initiation, we will conduct log rank tests and construct Cox proportional hazard regression models to estimate hazard ratios and 95% confidence intervals for each pairwise comparison *(e.g. Group 2 vs. Group 1, Group 3 vs. Group 2, and Group 3 vs. Group 1)*. Log-log plots will be examined and Schoenfeld residuals used to test the proportional hazards assumption.

# **Statistical analysis of secondary outcomes**

To analyse secondary outcomes (proportion with same-day tuberculosis treatment initiation, proportion with undiagnosed/untreated pulmonary tuberculosis, proportion with undiagnosed/untreated HIV, proportion reported to have died by Day 56, proportion with successful TB treatment outcome), we will construct log-binomial regression models to estimate relative risk ratios and 95% confidence intervals, comparing between pairs of groups. We will additionally compare between pairs of groups the time to antiretroviral therapy initiation among participants with previously untreated HIV using Cox regression models.

To evaluate the effect of interventions on health-related quality of life, we will use analysis of covariance ANCOVA analysis to compare the mean EQ5D utility scores and visual analogue scale scores measured at Day 56 between pairs of groups, adjusting for participants’ correspoding values measured at Day 0.

For the preplanned subgroup analysis of the primary trial outcome we will construct Cox proportion hazard regression models including a term for either sex (male or female) or microbiological TB status (either microbiologically confirmed or clinically-diagnosed) to estimate hazard ratios and 95% confidence intervals. We will use the likelihood ratio test to look for interactions between covariates and the trial group term.

# **Bayesian analysis of primary trial outcome**

Using within and between participant elicited probability distributions, we will construct stakeholder group-specific pooled prior probability distributions (known as a “community of priors”). Each prior will be converted to a log-hazard ratio scale and fitted to a normal distribution using a hierarchical random-effects model, with terms for stakeholder group, and a random-effects term to account for correlation of guesses within individuals. This will allow comparison between stakeholder groups of the similarity in support of opinions of effectiveness and of uncertainty.

Using Bayes’ theorem, we will combine elicited stakeholder group-specific log hazard ratio prior distributions with log-likelihood hazard ratio distributions from each pairwise comparison being made in the PROSPECT Study to construct posterior probability distributions. All analysis will be done in R and posterior mean hazard ratios and 95% credible intervals will be estimated by taking 4000 post-warmup draws from the posterior distributions using the No-U-Turn Sampler (NUTS) implemented with Stan.

It is anticipated that posterior distributions will demonstrate convergence between and within the sceptical and enthusiastic tails of stakeholder group prior distributions, indicating greater likelihood of accepting evidence. We will compare these findings with an analysis using a diffuse prior to investigate the impact of the prior specification on the posterior distribution. Finally, we will compare results obtained from the Bayesian analysis with that obtained from the main frequentist analysis.

# **Appendix**

This Appendix shows figures and tables for key results that will be reported by the study.

**Figure 1: Trial profile**


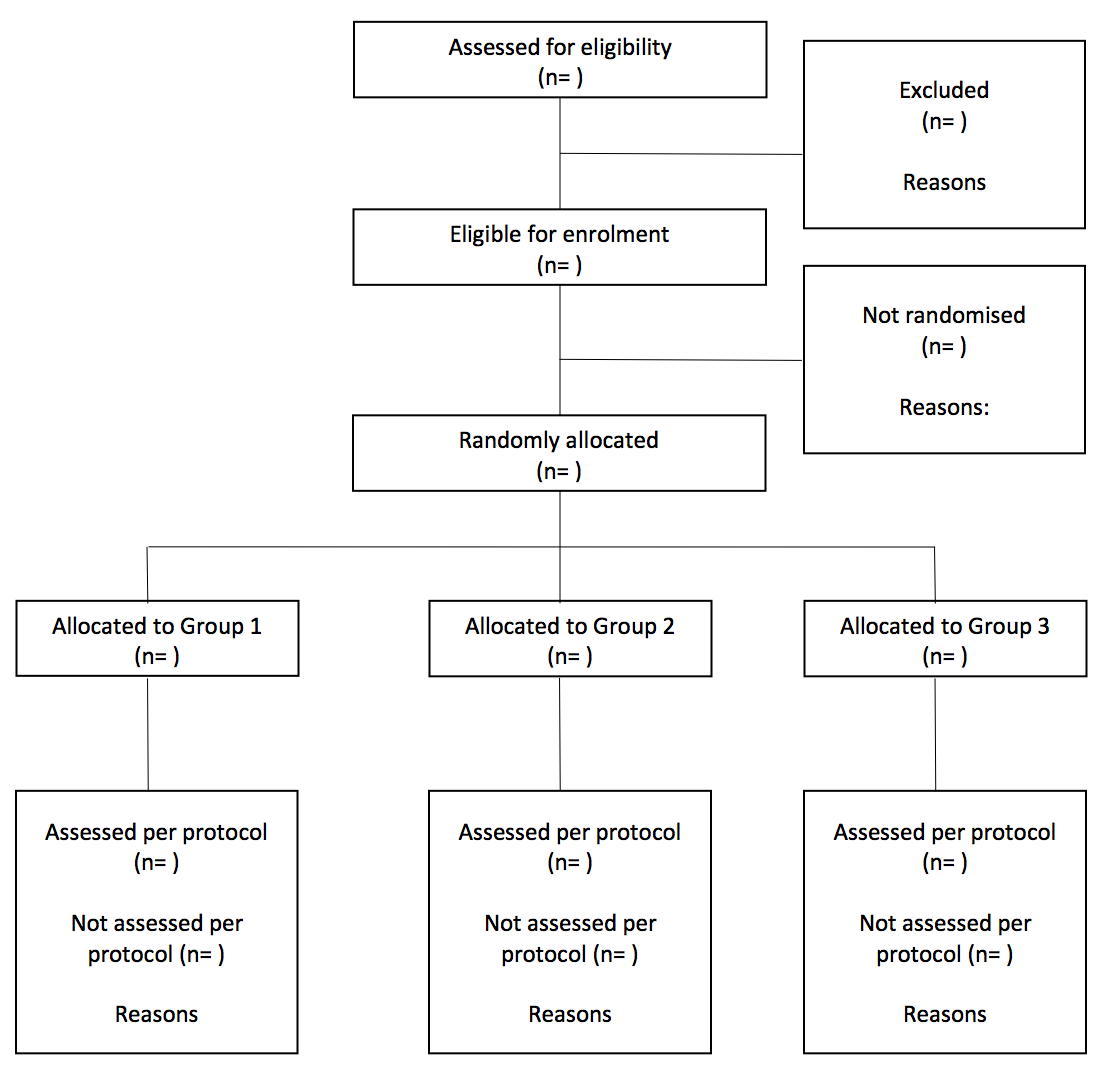


**Table 1: Baseline characteristics**

| **Characteristic** | **Group 1** | **Group 2** | **Group 3** |
| --- | --- | --- | --- |
| Age in years (mean, sd) |  |  |  |
| Sex |  |  |  |
| Male (n, %) |  |  |  |
| Female (n, %) |  |  |  |
| BMI (mean kg/m^2^, sd) |  |  |  |
| Marital status |  |  |  |
| Married/cohabiting (n, %) |  |  |  |
| Never married (n, %) |  |  |  |
| Widowed/separated/divorced (n, %) |  |  |  |
| Highest level of education |  |  |  |
| No schooling (n, %) |  |  |  |
| Primary (n, %) |  |  |  |
| Secondary no MSCE (n, %) |  |  |  |
| Secondary with MSCE (n, %) |  |  |  |
| Higher (n, %) |  |  |  |
| Tuberculosis symptoms |  |  |  |
| Cough (n %) |  |  |  |
| Duration of cough (median weeks, IQR) |  |  |  |
| Night sweats (n %) |  |  |  |
| Weight loss (n %) |  |  |  |
| Fever (n %) |  |  |  |
| Previously treated for TB (n %) |  |  |  |
| HIV status |  |  |  |
| HIV-positive (n %) |  |  |  |
| Taking ART (n %) |  |  |  |
| HIV-negative (n %) |  |  |  |
| Unknown (n %) |  |  |  |
| EQ5D utility score (mean, sd) |  |  |  |
| EQ5D visual analogue scale score (mean, sd) |  |  |  |

* MSCE: Malawi Secondary Certificate of Education

**Table 2: Primary outcome: Effectiveness of interventions on time to TB treatment initiation**

| **Outcome** | **Group 1** | **Group 2** | **Group 3** | **HR (95% CI): Group 2 vs. 1** | **HR (95% CI): Group 3 vs. 2** | **HR (95% CI): Group 3 vs. 1** |
| --- | --- | --- | --- | --- | --- | --- |
| Median (IQR) time to TB treatment initiation (days) |  |  |  |  |  |  |

* HR: Hazard ratio; CI: Confidence interval

**Table 3: Secondary outcome: Effectiveness of interventions on same day TB treatment initiation**

| **Outcome** | **Group 1** | **Group 2** | **Group 3** | **RR (95% CI): Group 2 vs. 1** | **RR (95% CI): Group 3 vs. 2** | **RR (95% CI): Group 3 vs. 1** |
| --- | --- | --- | --- | --- | --- | --- |
| Same-day TB treatment initiation (n/N %) |  |  |  |  |  |  |

* RR: Risk ratio; CI: Confidence interval

**Table 4: Secondary outcome: Effectiveness interventions on undiagnosed/untreated microbiologically-confirmed TB**

| **Endpoint** | **Group 1** | **Group 2** | **Group 3** | **RR (95% CI): Group 2 vs. 1** | **RR (95% CI): Group 3 vs. 2** | **RR (95% CI): Group 3 vs. 1** |
| --- | --- | --- | --- | --- | --- | --- |
| Undiagnosed or untreated microbiologically-confirmed pulmonary TB (n, %) |  |  |  |  |  |  |

* RR: Risk ratio; CI: Confidence interval

**Table 5: Secondary outcome: Effectiveness interventions on undiagnosed/untreated HIV**

| **Endpoint** | **Group 1** | **Group 2** | **Group 3** | **RR (95% CI): Group 2 vs. 1** | **RR (95% CI): Group 3 vs. 2** | **RR (95% CI): Group 3 vs. 1** |
| --- | --- | --- | --- | --- | --- | --- |
| Undiagnosed or untreated HIV (n, %) |  |  |  |  |  |  |

* RR: Risk ratio; CI: Confidence interval

**Table 6: Secondary outcome: All-cause mortality**

| **Endpoint** | **Group 1** | **Group 2** | **Group 3** | **RR (95% CI): Group 2 vs. 1** | **RR (95% CI): Group 3 vs. 2** | **RR (95% CI): Group 3 vs. 1** |
| --- | --- | --- | --- | --- | --- | --- |
| Deaths (n, %) |  |  |  |  |  |  |

* RR: Risk ratio; CI: Confidence interval

**Table 7: Secondary outcome: Proportion with successful TB treatment outcome 6-months post treatment initiation**

| **Endpoint** | **Group 1** | **Group 2** | **Group 3** | **RR (95% CI): Group 2 vs. 1** | **RR (95% CI): Group 3 vs. 2** | **RR (95% CI): Group 3 vs. 1** |
| --- | --- | --- | --- | --- | --- | --- |
| Successful TB treatment outcome (n/N, %) |  |  |  |  |  |  |

* RR: Risk ratio; CI: Confidence interval

**Table 8: Secondary outcome: Mean difference in EuroQoL EQ5D utility score**

| **Endpoint** | **Group 1** | **Group 2** | **Group 3** | **RR (95% CI): Group 2 vs. 1^†^** | **RR (95% CI): Group 3 vs. 2^†^** | **RR (95% CI): Group 3 vs. 1^†^** |
| --- | --- | --- | --- | --- | --- | --- |
| Mean EuroQoL EQ5D utility score at Day 56 (sd) |  |  |  |  |  |  |

* RR: Risk ratio; CI: Confidence interval

† Adjusted for baseline EuroQoL EQ5D utility score

**Table 9: Secondary outcome: Mean difference in EuroQoL EQ5D visual analogue scale score**

| **Endpoint** | **Group 1** | **Group 2** | **Group 3** | **RR (95% CI): Group 2 vs. 1^†^** | **RR (95% CI): Group 3 vs. 2^†^** | **RR (95% CI): Group 3 vs. 1^†^** |
| --- | --- | --- | --- | --- | --- | --- |
| Mean EuroQoL EQ5D visual analogue scale score at Day 56 (sd) |  |  |  |  |  |  |

* RR: Risk ratio; CI: Confidence interval

† Adjusted for baseline EuroQoL EQ5D visual analogue scale score

**Table 10 Pre-specified subgroup analysis: Time to TB treatment initiation by sex**

| Outcome | Group 1 | Group 2 | Group 3 | HR (95% CI): Group 2 vs. 1 | HR (95% CI): Group 3 vs. 2 | HR (95% CI): Group 3 vs. 1 |
| --- | --- | --- | --- | --- | --- | --- |
| *Men:* Median (IQR) time to TB treatment initiation (days) |  |  |  |  |  |  |
| *Women:* Median (IQR) time to TB treatment initiation (days) |  |  |  |  |  |  |

* HR: Hazard ratio; CI: Confidence interval

**Table 11 Pre-specified subgroup analysis: Time to TB treatment initiation by microbiological TB status**

| Endpoint | Group 1 | Group 2 | Group 3 | HR (95% CI): Group 2 vs. 1 | HR (95% CI): Group 3 vs. 2 | HR (95% CI): Group 3 vs. 1 |
| --- | --- | --- | --- | --- | --- | --- |
| *Microbiologically-confirmed pulmonary TB:* Median (IQR) time to TB treatment initiation (days) |  |  |  |  |  |  |
| *Clinically-diagnosed TB:* *Men:* Median (IQR) time to TB treatment initiation (days) |  |  |  |  |  |  |

* HR: Hazard ratio; CI: Confidence interval
