# Supplementary material for: Computer-aided X-ray screening for tuberculosis and HIV testing among adults with cough in Malawi (the PROSPECT study): A randomised trial and cost-effectiveness analysis
Source: PLoS Med. 2021 Sep 9;18(9):e1003752. doi: 10.1371/journal.pmed.1003752 (PMC8459969; doi:10.1371/journal.pmed.1003752)
Supplement: S3 Text — (PDF) [file pmed.1003752.s005.pdf]

CONSORT Checklist

Section/Topic	Item No	Checklist item	Reported in section
**Title and abstract**			
	1a	Identification as a randomised trial in the title	Title Page
	1b	Structured summary of trial design, methods, results, and conclusions (for specific guidance see CONSORT for abstracts)	Abstract
**Introduction**			
Background and objectives	2a	Scientific background and explanation of rationale	Introduction, Paragraphs 1-4
	2b	Specific objectives or hypotheses	Introduction, Paragraph 5
**Methods**			
Trial design	3a	Description of trial design (such as parallel, factorial) including allocation ratio	Methods, Paragraph 1
	3b	Important changes to methods after trial commencement (such as eligibility criteria), with reasons	NA
Participants	4a	Eligibility criteria for participants	Methods, Paragraph 2
	4b	Settings and locations where the data were collected	Methods, Paragraph 1
Interventions	5	The interventions for each group with sufficient details to allow replication, including how and when they were actually administered	Methods, Paragraph 4-7
Outcomes	6a	Completely defined pre-specified primary and secondary outcome measures, including how and when they were assessed	Methods, Paragraph 9
	6b	Any changes to trial outcomes after the trial commenced, with reasons	NA
Sample size	7a	How sample size was determined	Methods, Paragraph 10
	7b	When applicable, explanation of any interim analyses and stopping guidelines	NA
Randomisation:			
Sequence	8a	Method used to generate the random allocation sequence	Methods,

generation			Paragraph 3
	8b	Type of randomisation; details of any restriction (such as blocking and block size)	Methods, Paragraph 3
Allocation concealment mechanism	9	Mechanism used to implement the random allocation sequence (such as sequentially numbered containers), describing any steps taken to conceal the sequence until interventions were assigned	Methods, Paragraph 3
Implementation	10	Who generated the random allocation sequence, who enrolled participants, and who assigned participants to interventions	Methods, Paragraph 3
Blinding	11a	If done, who was blinded after assignment to interventions (for example, participants, care providers, those assessing outcomes) and how	Methods, Paragraph 3
	11b	If relevant, description of the similarity of interventions	Methods, Paragraph 4-7
Statistical methods	12a	Statistical methods used to compare groups for primary and secondary outcomes	Methods, Paragraph 11
	12b	Methods for additional analyses, such as subgroup analyses and adjusted analyses	Methods, Paragraph 11
**Results**			
Participant flow (a diagram is strongly recommended)	13a	For each group, the numbers of participants who were randomly assigned, received intended treatment, and were analysed for the primary outcome	Figure 1, Results, Paragraph 1 & 4
	13b	For each group, losses and exclusions after randomisation, together with reasons	Results, Paragraph 4
Recruitment	14a	Dates defining the periods of recruitment and follow-up	Results, Paragraph 1
	14b	Why the trial ended or was stopped	Results, Paragraph 4
Baseline data	15	A table showing baseline demographic and clinical characteristics for each group	Table 1, Results, Paragraph 2
Numbers analysed	16	For each group, number of participants (denominator) included in each analysis and whether the analysis was by original assigned groups	Results, Paragraph 4

Outcomes and estimation	17a	For each primary and secondary outcome, results for each group, and the estimated effect size and its precision (such as 95% confidence interval)	Results, Paragraph 5-7
	17b	For binary outcomes, presentation of both absolute and relative effect sizes is recommended	NA
Ancillary analyses	18	Results of any other analyses performed, including subgroup analyses and adjusted analyses, distinguishing pre-specified from exploratory	NA
Harms	19	All important harms or unintended effects in each group (for specific guidance see CONSORT for harms)	NA
**Discussion**			
Limitations	20	Trial limitations, addressing sources of potential bias, imprecision, and, if relevant, multiplicity of analyses	Discussion, Paragraph 8
Generalisability	21	Generalisability (external validity, applicability) of the trial findings	Discussion, Paragraph 7-8
Interpretation	22	Interpretation consistent with results, balancing benefits and harms, and considering other relevant evidence	Discussion, Paragraph 1 & 9
**Other information**			
Registration	23	Registration number and name of trial registry	Abstract
Protocol	24	Where the full trial protocol can be accessed, if available	Supplementary material
Funding	25	Sources of funding and other support (such as supply of drugs), role of funders	Supplementary material
