# Supplementary material for: Computer-aided X-ray screening for tuberculosis and HIV testing among adults with cough in Malawi (the PROSPECT study): A randomised trial and cost-effectiveness analysis
Source: PLoS Med. 2021 Sep 9;18(9):e1003752. doi: 10.1371/journal.pmed.1003752 (PMC8459969; doi:10.1371/journal.pmed.1003752)
Supplement: S4 Text — (DOCX) [file pmed.1003752.s006.docx]

**S4 Text: Economic evaluation**

**Study overview**

A within-trial health economic evaluation was undertaken to assess the cost-effectiveness of the two optimized TB/HIV screening interventions in comparison to standard of care for patients attending Bangwe Health Centre, Malawi for acute care with symptoms of tuberculosis (cough of any duration). The economic evaluation took the perspective of the Malawi Ministry of Health. Public health services in Malawi are provided free of charge to patients at the point of delivery.

A cost-utility analysis was undertaken to estimate the incremental cost per quality-adjusted life-year (QALY) gained. All costs were expressed in 2018 US dollars. The time horizon of the trial was 56 days and therefore costs and health outcomes were not discounted. A detailed description of the study procedures has been published^1^.

**Healthcare resource use and costs**

All participants randomised to the three trial arms had healthcare resource use collected at baseline on the components of the intervention received. This included whether or not they had received an HIV self-test; confirmatory HIV testing; digital chest x-ray; and sputum testing using GeneXpert MTB/Rif. Current HIV treatment, including antiretroviral drug regimen, was captured on those who reported positive-HIV status.

Additional healthcare resource use was collected on participants’ day 56 outcome assessment visit, either at the clinic or through tracing for those who did not attend. At these visits participants were asked a standard set of questions to establish whether they had attended any outpatient health clinics or been admitted to hospital as inpatients over the preceding 56 days. Those who responded yes to outpatient clinic attendance had further questions on the frequency of, and reasons for these visits, including any investigations performed or treatments given. Those who responded yes to inpatient hospital admission had further questions on the primary medical reason for their admission and duration of their inpatient stay. All participants were asked about HIV and TB treatment history over the preceding 56 days.

All healthcare resource use items were converted to costs using the units costs estimated in this study (e.g. direct interventions costs) or from recent primary costing studies undertaken in the same setting (Blantyre, Malawi) by the health economist on this study^2-4^. Costs for drugs, including those given for TB and HIV treatment were based on the international market price^5^. Table A provides unit costs for all healthcare resource use items and the source of the costs.

We undertook a primary costing study to estimate the unit cost of a digital CXR. We first estimated the total annual cost and then divided this by the likely number of digital CXRs that would be performed per year at Bangwe Clinic if the intervention was provided to all eligible outpatients. The total costs included: salary of the radiographer; digital x-ray machine; batteries and back-up power supply; internet connectivity and cloud archiving service; CAD4TB related items; staff training; servicing costs; and other overheads. Equipment, licence and servicing costs were annuitized over their useful life with an annual discount rate of 3%. The number of digital CXRs performed per year was based on the total number of participants randomised in the trial extrapolated to a one-year time horizon.

We undertook primary costing study to also estimate the intervention related HIV self-test (HIVST) episode and GeneXpert test. For the HIVST episode we estimated separate costs for those who had just an HIVST (i.e. tested negative) and those who had an HIVST with subsequent confirmatory HIV testing.

**Table A: Unit costs of healthcare resource use items**

| **Healthcare resource** | | **Unit cost**  **(2018 US Dollars)** | **Source** |
| --- | --- | --- | --- |
| **Intervention related** | Outpatient clinic visit | 6.00 | Maheswaran et al^3^ |
|  | HIVST only | 8.65 | Primary costing |
|  | HIVST + confirmatory testing | 10.28 |  |
|  | Digital CXR | 10.98 |  |
|  | Sputum for GeneXpert | 13.29 |  |
| **Outpatient visits** | Outpatient clinic visit | 6.00 | Maheswaran et al^3^ |
|  | Sputum Smear (AFB/Microscopy)* | 9.58 | Maheswaran et al^4^ |
|  | Sputum Culture* | 28.48 | Maheswaran et al^4^ |
|  | Standard finger-prick HIV testing and counselling | 14.71 | Maheswaran et al^2^ |
|  | Standard CXR* | 17.54 | Maheswaran et al^4^ |
|  | Full Blood Count* | 8.06 | Maheswaran et al^4^ |
| **Inpatient admission**  **(cost per day of admission)** | Acute gastrointestinal infection | 37.77 | Maheswaran et al^4^ |
|  | Anaemia | 54.65 |  |
|  | Candidiasis | 50.88 |  |
|  | Chronic gastrointestinal infection | 82.09 |  |
|  | Congestive Heart Failure | 45.48 |  |
|  | Hypertension | 41.64 |  |
|  | Malaria | 72.21 |  |
|  | Musculoskeletal | 38.57 |  |
|  | Neoplasm | 34.46 |  |
|  | Other cardiovascular | 49.03 |  |
|  | Other respiratory | 42.39 |  |
|  | Pneumocystis Carinii Pneumonia | 42.80 |  |
|  | Upper gastrointestinal disorders | 52.84 |  |
|  | Other/Did not know | 52.55 |  |

*based on resources used to perform investigation in hospital

**Health outcomes**

Health outcomes for the economic evaluation were measured in quality-adjusted life-years (QALYs). Trial participants completed the approved Chichewa language version of the EuroQoL EQ-5D-3L^6^ at baseline and on their day 56 outcome assessment visit.

Participants completed both the descriptive EQ-5D-3L system and the accompanying visual analogue scale (VAS). Responses to the EQ-5D-3L instrument were converted to EQ-5D utility scores using the Zimbabwean EQ-5D tariff set^7^. The Zimbabwean tariff set generates utility scores ranging between -0.145 and 1.0, with 1.0 corresponding to “perfect health” and 0 representing a health state considered to be equivalent to death. The visual analogue scale is similar to a thermometer, and ranges from 100 (best imaginable health state) to 0 (worst imaginable health state). For our sensitivity analysis we also derived EQ-5D utility scores using the UK EQ-5D tariff set. The UK tariff set often equates severe health states to lower health utility scores.

Quality-adjusted life-years (QALYs) were generated for each trial participant using the area under the baseline-adjusted utility curve, assuming linear interpolation between the assessment points.

**Cost-effectiveness analysis**

As missing data is a common occurrence in randomised clinical trials, we explored the occurrence of missing data and the pattern of missingness to assess whether the assumptions of missing at random (MAR) were violated. In this trial healthcare resource and EQ-5D-3L data were only collected on recruitment and on the final day 56 outcome assessment. There were no missing data on recruitment into the trial.

As missing data were an occurrence and MAR assumptions were met, we undertook multiple imputation using chained equations to impute missing values for cost and EQ-5D utility scores for trial participants who could not be traced at Day 56.^8^ Our imputation models included current antiretroviral treatment status, age, sex, and socio-economic variables. We used predictive mean matching to impute missing values for cost and EQ-5D utility scores as they were non-normally distributed, and to ensure imputed costs were non-negative.^9^ Imputation was run 10 times and all the primary cost-effectiveness analysis was undertaken with the imputed datasets.

We made comparisons between the trial arms with regards to the mean total costs, mean total post-intervention costs (i.e. outpatient and inpatient healthcare utilisation after receiving intervention) and mean total QALYs accrued. The results of the cost-effectiveness analysis were expressed as the incremental cost-effectiveness ratio (ICER). In this trial we estimated the mean incremental cost and incremental QALYs by comparing the two intervention trial arms (HIV screening; TB-HIV screening) to the standard of care (SOC) arm. The ICER was calculated by dividing incremental costs by incremental QALYs gained. We used the seemingly unrelated regression model (sureg) estimator for our multivariable analysis to estimate the incremental costs and QALYs over the 56-day trial horizon.

All mean and incremental costs and QALYS accrued were estimated through non-parametric bootstrap methods with 1000 replications to provide bias-corrected 95% confidence intervals (CI).^10^ We used cost-effectiveness scatter plots to present the 1000 bootstrapped replications for incremental costs and incremental QALYs.

Malawi does not have formal cost-effectiveness threshold. We therefore compared the estimated ICERs to WHO-recommended thresholds using the gross domestic product (GDP) per capita for the country. Interventions that have an incremental cost per gain in QALY less than the national GDP per capita were defined as “*very cost-effective”*, and those less than three times GDP per capita as “*cost effective”*^11^. The GDP per capital of Malawi is approximately US400 per capital. We therefore used the bootstrapped replications to present the probability the two interventions (HIV screening; TB-HIV screening) were cost-effective at increasing cost-effectiveness thresholds: US$400/QALY; US$800/QALY; US$1200/QALY. The probability represents the proportion of the 1000 bootstrapped replications where the estimated ICER was below these cost-effectiveness thresholds.

**Sensitivity analysis**

We undertook a range of sensitivity analysis. We undertook a complete case analysis estimating the ICER using cost and EQ-5D utility data from trial participants for whom data was available. We also estimated the ICERs using EQ-5D data derived using the UK tariff set. We also estimated the ICERs had the cost of digital CXR been only US$5. The cost of digital CXR will potentially be lower in a busier clinic that sees more individuals with a cough; or if implemented at scale.

**Results**

**Costs and health outcomes**

Table B shows the number of participants with missing EQ-5D and resource use data at baseline and on day 56. The proportion of participants with missing data was comparable across the three arms.

**Table B: Missing heath economics data by treatment allocation**

| **Variable** | **Missing values** | | |
| --- | --- | --- | --- |
|  | **SOC**  **(n=472)** | **HIV screening (n=492)** | **TB-HIV screening (n=497)** |
| EQ-5D utility score – baseline | 0 (0) | 0 (0) | 0 (0) |
| EQ-5D utility score – day 56 | 89 (18.9) | 77 (15.6) | 81 (16.3) |
| QALYs generated from EQ-5D utility scores | 89 (18.9) | 77 (15.6) | 81 (16.3) |
| Resource use at baseline | 0 (0) | 0 (0) | 0 (0) |
| Resource use between baseline and day 56 | 58 (12.3) | 46 (9.3) | 48 (9.7) |

Table C shows the healthcare resources used by participants across the three arms. The uptake of HIV testing was comparable between the HIV screening and TB-HIV screening arms. The majority of participants in the TB-HIV screening arm had a digital CXR performed whilst 56.1% had a sputum processed using GeneXpert MTB/Rif.

Table D shows the direct medical costs by treatment allocation. The mean total direct medical cost for the HIV screening and TB-HIV screening arms were comparable. The mean total direct medical cost for the TB-HIV screening arm was US$20.14 (bootstrap 95%CrI: US$14.51 to US$25.76) higher than for the SOC arm. The mean total direct medical cost for the TB-HIV screening arm was US$16.61 (bootstrap 95%CrI: US$13.42 to US$19.79) higher than for the HIV screening arm. In comparison to SOC, the mean direct medical cost incurred from inpatient care was significantly lower for the HIV screening and TB-HIV screening arms. As expected, the total direct medical costs of providing the intervention was highest for the TB-HIV screening arm.

**Table C: Healthcare resources use by treatment allocation***

|  | **Treatment allocation** | | |
| --- | --- | --- | --- |
|  | **SOC**  **(n/N & %)** | **HIV screening (n/N & %)** | **TB-HIV screening (n/N & %)** |
| **Intervention related resources**  HIV testing  Digital CXR  Sputum Gene Xpert | 0 (0)  0 (0)  0 (0) | 328/492 (66.7)  0 (0)  0 (0) | 358/497 (72.0)  448/497 (90.1)  279/497 (56.1) |
| **Resource use between baseline and Day 56**  HIV treatment  TB treatment**  Hospital admission  Outpatient clinic visit | 93/472 (19.7)  5/472 (1.1)  11/415 (2.7)  90/415 (21.7) | 119/492 (24.2)  8/492 (1.6)  9/446 (2.0)  93/446 (20.8) | 122/497 (24.5%)  14/497 (2.8)  3/449 (0.7)  100/449 (22.3) |

*complete case analysis

**TB-HIV screening arms excludes one participant who started TB treatment on Day 56 and therefore no healthcare resources used trial period

**Table D: Direct medical costs by treatment allocation (2018 US Dollars)***

|  | **Treatment allocation** | | | **Mean difference (95% CrI)**** | | |
| --- | --- | --- | --- | --- | --- | --- |
|  | **SOC**  **(mean/SE)** | **HIV screening**  **(mean/SE)** | **TB-HIV screening**  **(mean/SE)** | **HIV screening v SOC** | **TB-HIV screening v SOC** | **TB-HIV screening v HIV screening** |
| Direct Intervention | 6.00  (0) | 11.85  (0.19) | 29.66  (0.41) | 5.85  (5.48, 6.21) | 23.66  (22.90, 24.42) | 17.81  (16.93, 18.70) |
| HIV treatment | 5.57  (0.53) | 7.14  (0.58) | 7.30  (0.59) | 1.57  (0.07, 3.08) | 1.73  (0.23, 3.23) | 0.16  (-1.49, 1.80) |
| TB Treatment | 0.15  (0.07) | 0.26  (0.10) | 0.55  (0.15) | 0.12  (-0.13, 0.36) | 0.40  (0.08, 0.72) | 0.28  (-0.06, 0.63) |
| Outpatient care | 2.59  (0.35) | 1.88  (0.24) | 2.39  (0.29) | -0.71  (-1.56, 0.14) | -0.20  (-1.13, 0.72) | 0.51  (-0.23, 1.25) |
| Inpatient care | 7.02  (2.88) | 3.00  (1.23) | 0.68  (0.51) | -4.02  (-10.47, 2.43) | -6.34  (-12.25, -0.43) | -2.32  (-4.86, 0.021) |
| Post-intervention costs*** | 14.19  (2.77) | 11.88  (1.35) | 10.67  (0.86) | -2.31  (-8.43, 3.80) | -3.52  (-9.13, 2.08) | -1.21  (-4.43, 2.01) |
| Total costs | 20.19  (2.77) | 23.72  (1.31) | 40.33  (0.91) | 3.53  (-2.56, 9.62) | 20.14  (14.51, 25.76) | 16.61  (13.42, 19.79) |

*complete case analysis

**Bootstrapped differences (1000 replications) and credible interval (CrI)

***All costs excluding direct intervention costs

Table E shows the baseline and day 56 EQ-5D utility and VAS scores across the three arms, and the estimated mean differences between the arms. In comparison to SOC, the EQ-5D utility scores on day 56 were marginally lower in the HIV-screening (0.027; 95%CrI: 0.002 to 0.053) and TB-HIV screening (0.023; 95%CrI: -0.002 to 0.047) arms.

**Table E: Health-related quality of life outcomes by treatment allocation***

|  | **Treatment allocation** | | | **Mean difference (95% CrI)**** | | |
| --- | --- | --- | --- | --- | --- | --- |
|  | **SOC**  **(mean/SE)** | **HIV screening**  **(mean/SE)** | **TB-HIV screening**  **(mean/SE)** | **HIV screening v SOC** | **TB-HIV screening v SOC** | **TB-HIV screening v HIV screening** |
| Baseline EQ-5D utility score (Zim Tariff) | 0.786 (0.007) | 0.772 (0.007) | 0.770 (0006) | -0.014  (-0.032, 0.005) | -0.016  (-0.033, 0.002) | 0.002  (-0.017, 0.020) |
| Baseline EQ-5D utility score (UK Tariff) | 0.721 (0.010) | 0.699 (0.010) | 0.700 (0.010) | -0.021  (-0.050, 0.007) | -0.021  (-0.048, 0.007) | -0.001  (-0.030, 0.027) |
| Baseline VAS score | 64.9  (0.6) | 65.5  (0.6) | 64.4  0.6) | 0.57  (-1.17, 2.31) | -0.52  (-2.14, 1.11) | 1.09  (-0.60, 2.77) |
| Day 56 EQ-5D utility score (Zim Tariff) | 0.790 (0.009) | 0.817 (0.009) | 0.813 (0.009) | 0.027  (0.002, 0.053) | 0.023  (-0.002, 0.047) | -0.005  (-0.027, 0.017) |
| Day 56 EQ-5D utility score (Zim Tariff) | 0.725 (0.013) | 0.761 (0.013) | 0.756 (0.013) | 0.036  (-0.001, 0.073) | 0.031  (-0.006, 0.068) | -0.005  (-0.027, 0.017) |
| Day 56 VAS score | 73.3  (0.7) | 74.4  (0.7) | 73.7  (0.8) | 1.12  (-1.01, 3.26) | 0.39  (-1.67, 2.45) | -0.73  (-2.47, 1.02) |

*Unadjusted complete case analysis

**Bootstrapped differences (1000 replications) and credible interval (CrI)

**Cost-effectiveness analysis**

The findings of the within-trial cost-effectiveness analysis are shown in Table F. The incremental analysis presented compares the intervention arms (HIV screening; TB-HIV screening) to the SOC arm. Figure A shows the joint distribution for the incremental costs and incremental QALYs for HIV screening and TB-HIV screening arms when compared to SOC in the base case analysis.

In the base case analysis, the mean difference in direct medical costs were significantly higher for those in the HIV screening arm (US$3.58, 95%CrI: 1.70 to 5.45) compared to those in the SOC arm. The mean difference in direct medical costs were significantly higher for those in the TB-HIV screening arm (US$19.92, 95%CrI: 18.17 to 21.68) compared to those in the SOC arm. The mean difference in QALYs were significantly higher for those in the HIV screening arm (0.004, 95%CrI: 0.003 to 0.005) compared to those in the SOC arm. The mean difference in QALYs were significantly higher for those in the TB-HIV screening arm (0.004, 95%CrI: 0.003 to 0.005) compared to those in the SOC arm.

In the base-case analysis the ICER for HIV screening v SOC was US$901.29 per QALY gained; the ICER for TB-HIV screening v SOC was US$4620.47.36 per QALY gained. At the cost-effectiveness thresholds of US$400 per QALY, US$800 per QALY and US$1200 per QALY, the probability of cost-effectiveness for HIV screening was 0.030, 0.362 and 0.839, respectively. Across all these three cost-effectiveness thresholds the probability of cost-effectiveness for TB-HIV screening (in comparison to SOC) was 0. Figure A demonstrates that in comparison to SOC, participants in the HIV screening and TB-HIV screening arms experienced comparable gains in QALYS, whilst the incremental costs for TB-HIV screening was much greater than for HIV screening.

The sensitivity analysis supports the findings from base case analysis. Cost-effectiveness of the intervention arms improves when the UK tariff set was used to estimate EQ-5D utility scores. The ICER for TB-HIV screening v SOC is lower, had the cost of performing a digital CXR been US$5 instead of the estimated US10.98.

The within-trial cost-effectiveness analysis suggests that offering oral HIV testing to adults attending primary health care clinics with cough of any duration was cost-effective, potentially highly cost-effective. Offering TB-HIV screening through a combination of oral HIV testing, digital CXR and sputum for Gene Xpert was not cost-effective at ICER thresholds below US$1200 per QALY gained. The cost-effectiveness of TB-HIV screening improved if the cost of performing digital CXRs was lower.

**Further analysis**

The findings of the within-trial economic analysis are limited by the timeframe for which costs and consequences were considered. Further decision-analytic modelling is being undertaken to incorporate the long-terms costs and consequences of timely TB and HIV treatment; and potential impact on the incidence of these conditions from increased access to treatment.

**Table F: Health-related quality of life outcomes by treatment allocation***

|  | **Total Cost (mean/SE)** | **Incremental cost**  **(95% CrI)^†^** | **QALYs**  **(mean/SE)** | **Incremental QALYs (95% CrI)^†^** | **ICER^†^** | **Probability cost-effective at cost-effectiveness threshold:** | | |
| --- | --- | --- | --- | --- | --- | --- | --- | --- |
|  |  |  |  |  |  | **US$400/QALY** | **US$ 800/QALY** | **US$ 1200/QALY** |
| **Base case analysis^#^** | | | | | | | | |
| Standard of care | 21.45 (3.18) | - | 0.001 (0.002) | - | - | - | - | - |
| HIV screening | 24.29 (1.61) | 3.58  (1.70, 5.45) | 0.007 (0.002) | 0.004  (0.003, 0.005) | 901.29 | 0.030 | 0.362 | 0.839 |
| TB-HIV screening | 41.01 (1.17) | 19.92  (18.17, 21.68) | 0.007 (0.002) | 0.004  (0.003, 0.005) | 4620.47 | 0 | 0 | 0 |
| **Sensitivity analysis – Imputed data using UK Tarif to derive EQ-5D utility scores^#^** | | | | | | | | |
| Standard of care | 21.45 (3.18) | - | 0.001 (0.003) | - | - | - | - | - |
| HIV screening | 24.29 (1.61) | 3.58  (1.70, 5.45) | 0.010 (0.002) | 0.005  (0.004, 0.007) | 714.69 | 0.062 | 0.652 | 0.959 |
| TB-HIV screening | 41.01 (1.17) | 19.92  (18.17, 21.68) | 0.009 (0.002) | 0.005  (0.004, 0.007) | 3841.67 | 0 | 0 | 0 |
| **Sensitivity analysis – complete cases^#^** | | | | | | | | |
| Standard of care | 20.19 (2.77) | - | 0.001 (0.001) | - | - | - | - | - |
| HIV screening | 23.72 (1.31) | 3.91  (-2.72, 10.53) | 0.007 (0.002) | 0.004  (0.0005, 0.008) | 953.85 | 0.266 | 0.445 | 0.595 |
| TB-HIV screening | 40.33 (0.91) | 19.94  (13.50, 26.38) | 0.007 (0.002) | 0.005  (0.001, 0.009) | 4139.92 | 0.007 | 0.008 | 0.09 |
| **Sensitivity analysis – Imputed data using lower cost for digital CXR (US$5)*^#^** | | | | | | | | |
| Standard of care | 21.11 (2.95) | - | 0.001 (0.002) | - | - | - | - | - |
| HIV screening | 24.16 (1.42) | 3.57  (1.72, 5.41) | 0.007 (0.002) | 0.004  (0.003, 0.005) | 961.12 | 0.020 | 0.292 | 0.748 |
| TB-HIV screening | 35.56 (1.09) | 14.53  (12.74, 16.32) | 0.007 (0.002) | 0.004  (0.003, 0.005) | 3600.37 | 0 | 0 | 0 |

**^†^**Incremental estimates are all in comparison to standard of care arm

*In primary analysis cost of digital CXR was US$10.98

^#^Adjusted for age, sex, marital status, highest level of education, employment status and poverty quintile

Bootstrapped differences (1000 replications)

SE: standard error, CrI: credible interval, QALY: Quality adjusted life year, ICER: Incremental cost-effectiveness ratio

**Figure A: Cost-effectiveness scatter plot for primary analysis***

*Imputed data with Zimbabwean Tarif

**References**

1. MacPherson P, Webb EL, Lalloo DG, et al. Design and protocol for a pragmatic randomised study to optimise screening, prevention and care for tuberculosis and HIV in Malawi (PROSPECT Study). *Wellcome Open Res* 2018; **3**: 61.

2. Maheswaran H, Petrou S, MacPherson P, et al. Cost and quality of life analysis of HIV self-testing and facility-based HIV testing and counselling in Blantyre, Malawi. *BMC Med* 2016; **14**: 34.

3. Maheswaran H, Petrou S, MacPherson P, et al. Economic Costs and Health-Related Quality of Life Outcomes of HIV Treatment After Self- and Facility-Based HIV Testing in a Cluster Randomized Trial. *J Acquir Immune Defic Syndr* 2017; **75**(3): 280-9.

4. Maheswaran H, Petrou S, Cohen D, et al. Economic costs and health-related quality of life outcomes of hospitalised patients with high HIV prevalence: A prospective hospital cohort study in Malawi. *PLoS One* 2018; **13**(3): e0192991.

5. Health MSf. International Drug Price Indicator Guide. Available at: <https://www.msh.org/blog/2015/07/02/new-edition-of-international-drug-price-indicator-guide-available>. 2015.

6. Dolan P. Modeling valuations for EuroQol health states. *Medical care* 1997; **35**(11): 1095-108.

7. Jelsma J, Hansen K, De Weerdt W, De Cock P, Kind P. How do Zimbabweans value health states? *Popul Health Metr* 2003; **1**(1): 11.

8. White IR, Royston P, Wood AM. Multiple imputation using chained equations: Issues and guidance for practice. *Statistics in medicine* 2011; **30**(4): 377-99.

9. Briggs A, Clark T, Wolstenholme J, Clarke P. Missing... presumed at random: cost-analysis of incomplete data. *Health economics* 2003; **12**(5): 377-92.

10. Thompson SG, Barber JA. How should cost data in pragmatic randomised trials be analysed? *BMJ* 2000; **320**(7243): 1197-200.

11. WHO. Making choices in health: WHO guide to cost-effectiveness analysis. Available at: <http://www.who.int/entity/choice/publications/p_2003_generalised_cea.pdf>. . 2003.
