# Supplementary material for: Computer-aided X-ray screening for tuberculosis and HIV testing among adults with cough in Malawi (the PROSPECT study): A randomised trial and cost-effectiveness analysis
Source: PLoS Med. 2021 Sep 9;18(9):e1003752. doi: 10.1371/journal.pmed.1003752 (PMC8459969; doi:10.1371/journal.pmed.1003752)
Supplement: Attachment — Submitted filename: 2021-03-15_response-R1_PROSPECT.pdf [file pmed.1003752.s008.pdf]

Dr Peter MacPherson MBChB MPH PhD
Wellcome Trust Fellow & Reader in Population Health
Liverpool School of Tropical Medicine &
Malawi-Liverpool-Wellcome Trust Clinical Research Programme, Blantyre, Malawi

Honorary Professor in Population Health
London School of Hygiene and Tropical Medicine

Honorary Consultant in Communicable Disease Control
Public Health England

Mob: +265 99 717 6230
Email: peter.macpherson@lstmed.ac.uk

24/03/2021

Dear Dr Raffaella Bosurgi (Executive Editor, PLOS Medicine)

Re: PMEDICINE-D-20-05974R1. Computer-aided X-ray screening for tuberculosis and HIV testing: a randomised trial and cost-effectiveness analysis in Malawi (PROSPECT)

I write on behalf of my co-authors to thank the reviewers for their careful review, and appreciate the opportunity to respond with a revised submission.

Please see point-by-point responses below.

Yours Sincerely,

Peter MacPherson
(On Behalf of the Authors

Reviewer #1:

1. Computer aided diagnostic systems have been evaluated in my multiple studies and have been shown to have utility in triaging for TB diagnosis. The finding that use of CAD in the screening reduces the time to diagnosis is not a surprise finding.

Thank you for this comment. Although there have been multiple studies that have evaluated the diagnostic accuracy (sensitivity, specificity, etc) of CAD for triaging for TB, we are not aware of any previous randomised controlled trials that have evaluated the impact of CAD systems on patient outcomes, including timing and completeness of treatment initiation and mortality. Many countries, including Malawi, have now started implementing DCXR-CAD systems into health facilities. We therefore think this study is a valuable contribution to the literature, and should help guide implementation.

2. Previous studies evaluating CAD systems are not adequately referenced

Thank you for this comment. We have now additionally referenced both a previously published and newly published systematic review that summarises the diagnostic accuracy of CAD for tuberculosis systems (see Line 88 in the introduction).

3. There is not much description given of the HIV and TB programs in the setting. For example, in most high TB and HIV settings, opt out HIV testing is offered to all patients accessing services. The study suggests that this is not offered in routine services and that the study was offering this outside of routine services. Is this correct?

Thank you. We have added additional description of the HIV and TB programmes to clarify (Lines 107-112):

"Bangwe health centre is located within a densely-populated neighbourhood in the East of the city. Adult HIV prevalence in Blantyre is estimated to be 18%,[20] and Malawi urban TB prevalence was estimated at 900 per 100,000 in the most recent national TB prevalence survey.[21] Comprehensive HIV care (including ART) and TB treatment are available at primary care level through the national HIV and TB treatment programmes in Malawi."

Although national HIV guidelines in Malawi and in similar settings recommend routine "opt out" HIV testing for adults attending primary care, this is very often not completed under routine conditions (see Mair et al 2020, where clinic exit interviews showed that only 21% of eligible adult clinic attenders in Blantyre completed HIV testing and counselling). To overcome these barriers, we offered an oral HIV screening intervention delivered by research staff, with the aim of investigating whether this would reduce undiagnosed and untreated HIV (and TB) (Lines 142-143):

"Malawi National Guidelines recommend HIV testing for all adults attending health facilities, although this is often not completed.[20]"

4. CAD threshold setting is not fully described. How was the threshold of >=45 arrived at? The threshold setting is based on the prevalence of TB in the population being studied. Was this taken into consideration. Were there any patients with CAD score below 45 on whom Xpert was positive?

The CAD threshold was selected based on preliminary studies (described in the referenced study protocol), and with guidance from the manufacturer. We have added text to the Methods to report this (Lines 162-163):

"Where the CAD4TB score was ≥45 (threshold selected based on pilot studies and in discussion with the software developer) participants' sputum was tested onsite by Xpert;…"

Although it would have been ideal to have conducted a full diagnostic accuracy study prior to the trial to guide threshold selection, this would have been extremely expensive due to the large numbers of TB microbiological tests required, and the low prevalence of disease in the population. We have added text to the discussion to emphasise these points (Lines 428-434):

"Importantly, although CAD thresholds would ideally be adapted to local epidemiological characteristics and available resources, the large numbers of participants required and cost of undertaking diagnostic accuracy studies often precludes this, as in this trial. To minimise the need for multiple diagnostic accuracy studies with microbiological reference standards prior to implementation, modelling of accuracy at varying thresholds—as well as repeated surveillance and threshold adaption under programmatic conditions—will be required."

Baseline sputum Xpert was not done for participants whose CAD4TB score was <45 (see Methods, Lines 158-169). This was because CAD4TB was being used as a "triage test" to determine whether sputum Xpert should be done or not.

5. Since there is limited CXR accessibility in most TB and HIV high burden countries, the authors could have considered a HIV-TB screening arm (without CXR) and HIV-TB screening arm (with CXR) and compare the findings. TB screening is recommended for every HIV positive patient whenever in contact with the health services- generating evidence for the impact of on time to TB treatment, TB diagnosis can support the scale up of routine TB screening in HIV positive patients even when there is no CXR with CAD.

Thank you for this suggestion. Our intervention was selected so that, if effective, it would be feasible to implement under routine programmatic conditions. We have previously shown (Feasey et al 2021) that under routine conditions, completion of TB screening based on symptom screen with subsequent sputum examination, is very low (0.2% symptomatic adults eligible for screening receiving same-day sputum results). Given these data, we are sceptical that an intervention comprised of TB symptom screening and sputum examination would be sufficient to appreciably increase diagnosis and reduce time to treatment, but we agree that this might be an interesting future trial. CXR is recognised by WHO as an essential medical diagnostic, and is becoming increasingly available in primary care, even in low resource settings such as Blantyre (following on from this trial digital chest X-ray and CAD are now available in several primary health care centres in the city). CAD additionally facilitates automated triage of patients based on their CXR where availability of trained readers is low, and this is why we set out to investigate this high-throughput, high sensitivity initial triage test (CXR with CAD) in this trial.

6. It would be helpful to report the retention at day 56 instead of leaving it to the readers to calculate. If there were any drop outs, what was the reason for drop out (or not returning at day 56)- If unknown or due to death can the assumption be made that some patients could have died from undiagnosed TB?

We have included data on numbers lost to follow-up in the CONSORT diagram (Figure 1) and have added text to the results describing the overall number and % with outcomes ascertained at day 56. We have additionally added text to describe procedures undertaken to trace participants to home who did not attend their Day 56 outcome assessment clinic visit (Lines 175-176).

We agree that it is possible that some individuals who died or who were not traceable may have died from undiagnosed TB, but have no way of determining this.

Reviewer #2:

The manuscript by MacPherson et al from the Malawi research program supported by the Liverpool and London Schools of Hygiene and Tropical Medicine investigates the impact of an enhanced approach to TB diagnosis in early detection of TB in PLHIV and early TB treatment. A single clinic site, Bangwe Health Centre in Blantyre was chosen for the study. The diagnostic enhancement is a study staff controlled digital chest Xray with an interpretation software application followed by sputum Gene Xpert probe for all x-rays likely to be TB above a pre-set cutoff. The impact was compared with 'Standard of Care' managed by Ministry of Health staff and a second comparator group of study staff-managed 'oral HIV screening'. The main finding was median time to TB treatment which was 11 days for SoC, 6 days for oral HIV and 1 day for HIV and TB. The Hazard Ratio for HIV-TB compared with SoC is calculated at 2.84 which was statistically significant. A cost analysis is reported in a subsidiary section and found no chance of cost-effectiveness for the HIV-TB intervention although oral HIV screening was probably cost effective in the short term.

1. The primary audience for such studies should be Departments of Public Health, in particular in this case the Malawi Ministry of Health, but they may not find this study as helpful as it could have been. My main criticism is that, although the study is described as 'pragmatic', it is designed to measure the impact of complex and expensive pieces of technology to diagnose TB. A number of 'pragmatic' studies over the last 5 years of 'breakthrough' molecular diagnostics for TB, implemented in LMIC health systems, have been disappointing. One of the main reasons is that the systems - human resources, facilities, supply chains etc. - are not reliable enough to deliver the increased sensitivity and specificity of new technology.

Thank you for these helpful comments. We agree that substantial health systems limitations remain in high HIV/TB burden countries. However, we believe that novel diagnostics should be evaluated in the communities that require them most, in conjunction with interventions to strengthen health systems. We are undertaking modelling, working with policymakers in the National TB programme, and are planning further trials and implementation evaluations to investigate how best to overcome these limitations.

2. In this study, performed at a single site that accommodated all three arms, two of the arms were supervised by 'study staff' that have a large impact on implementation and adherence to standards of care, compared with the Ministry public health workers supervising the SoC arm. Thus there was benefit observed in the oral HIV arm supervised by study staff over the SoC arm, even though no direct TB diagnostic enhancement was used. It is not

mentioned how much the study staff added to the cost of care but the impact of 'oral HIV' arm combined with its cost-effectiveness may have been more impressive, sustainable, and attractive to public servants than the high technology solution.

We undertook primary costing studies to estimate the costs of each intervention. For an oral HIVST episode, this included the costs to employ an HIV counsellor and their associated training, as well as clinic space and equipment/consumables needed to deliver the supervised HIVST. We have amended the Appendix Page 2 to highlight what resources were included in our estimate of the HIVST episode. We agree that the HIV testing arm may be more attractive to policymakers currently, and think that this is an important finding. Despite HIV treatment coverage being very high in Malawi, the HIV testing intervention substantially improved HIV diagnosis and treatment initiation and was cost-effective within the trial follow-up horizon (over the 56-day follow-up period). Of note, for the TB screening intervention, the economic evaluation was also conducted within the trial horizon (over the 56-day follow-up period); given the trial findings, we anticipate that any benefits in terms of health, epidemiological or economic impact may be accrued over longer time periods. We are therefore currently conducting transmission and economic modelling to investigate the optimal approaches to implementation over longer time horizons with scale-up in setting such as Malawi. These modelling analyses will be reported separately.

3. As far as the report in itself - it is well written, pretty clear, and the figures and analysis seem clear and supportable. Some specific criticisms - the digital Xray platform and interpretation software is not well described.

We have added additional information about the digital x-ray platform and CAD system (Lines 158-163):

"In addition to the HIV testing intervention, participants in the HIV-TB screening arm were offered a single posterior-anterior digital chest X-ray (MinXray Commander CMDR.T.120.60. S, USA) done by study radiographers with interpretation using CAD4TB v5 (Delft Imaging, Netherlands). CAD4TB interpretation was done locally on a dedicated computer, with results automatically transferred to study data collection devices."

4. The study staff are not described - how many people, what level of training, location within Bangwe Clinic.

Thank you. We have added additional information to Lines 115-116 and 133-134 to describe:

"[…] were screened by study research assistants (six in total, not medically trained, but with experience in community and clinic-based research) at the clinic registration desk."

"All participants underwent a baseline questionnaire by study research assistants, conducted in a dedicated study research building within the grounds of the clinic."

5. A brief discussion of 'cross-contamination' between the study and SoC within a single clinic is included but I think this could be a major issue.

We undertook extensive precautions to minimise contamination (including fingerprint bioidentification of participants prior to randomisation and delivering of interventions), and these issues were discussed in the published trial protocol (MacPherson et al 2018), and in the Discussion of this manuscript (Lines: 470-476). Study interventions (including chest x-ray and CAD and oral HIV testing) were done by study research assistants in a separate research building within the grounds of the clinic. One potential alternative study design to further reduce potential contamination would have been to conduct a clinic-level cluster randomised trial. However, this would have required a large number of study clinics to be randomised, probably requiring a multicountry study, and because multiple x-ray units and CAD systems would have had to have been procured, would have been orders of magnitude more expensive. Given the promising evidence reported here, we believe that such a trial would now be warranted.

6. Finally, Malawi generally has implemented an efficient national ART program with excellent coverage and adherence and this may well be responsible for unexpectedly low rates of community TB and present the most cost-effective approach. A discussion of the use of enhanced TB diagnosis in places where there is a much lower ART program efficiency should be included.

We agree that the Malawi National HIV Programme has made tremendous progress in achieving high levels of HIV testing and ART coverage, and that these interventions may be more effective in other settings. We have added text to the discussion to emphasise this point (Lines 480-481).

"Malawi has achieved very high levels of HIV testing and ART coverage; the effectiveness of these interventions should be evaluated in other settings."

Reviewer #3

1. The paper reports an innovative approach to try and improve diagnosis of both TB and HIV in a high HIV prevalence setting among people presenting to the clinic with cough. The study provides additional useful information on the importance of oral HIV self-testing, however, the importance of oral HIV self-testing in Blantyre and similar high HIV prevalence settings and OPD settings has been well described. The use of Computer-aided X-ray screening for TB is innovative, but findings are limited to patients with cough presenting to the facility, which needs to be clear in the title.

Thank you. We have updated the title to reflect this:

"Computer-aided X-ray screening for tuberculosis and HIV testing among adults with cough: a randomised trial and cost-effectiveness analysis in Malawi (PROSPECT)"

2. In addition, the combined HIV and TB screening intervention was not cost-effective. The paper provides important data points in this field, but the authors could and, in my opinion, should do more to describe the considerable cost, feasibility, and human resource challenges associated with rolling out Computer-aided X-ray screening in low-and middle income countries like Malawi where the burden of undiagnosed TB is highest.

Thank you. As noted above, Ministries of Health in Malawi and several other countries in the region have already began implementing DCXR-CAD, both within health centres and as part of mobile TB active case finding vans. In Blantyre the National TB Programme has installed several DCXR-CAD systems in primary health facilities. We therefore believe that robust evidence about cost-effectiveness of these interventions is important to guide implementation. We agree that there can be challenges in deploying DCXR-CAD, and we have added additional text to describe issues in implementation of computer-aided chest x-ray screening in settings like Malawi. (Lines 379-385):

"However, implementation of DCXR-CAD in high HIV/TB burden settings where they are most needed can be challenging, and requires careful consideration by Ministries of Health, as well as technological optimisation for low-resource settings and operational research to support deployment. Particular challenges can include absent or intermittent power supplies, limited internet availability, availability of maintenance and servicing personnel, and laboratory capacity to handle potential increased sputum specimen samples for confirmatory testing, although these were all overcome in this trial."

3. The study population represents symptomatic persons attending acute primary care clinics:
- The title could include the population studied such as: "Computer-aided X-ray screening for tuberculosis and HIV testing among adults presenting with cough: a randomised trial and cost-effectiveness analysis in Malawi (PROSPECT)". This is important because a significant percentage of patients with active TB are asymptomatic. In addition, the four-symptom TB screen is only able to detect 51% of active TB cases among PLHIV on ART. Therefore, this is an intervention targeted at detecting symptomatic TB where the symptom is cough (and HIV infection among people with at least one symptom (cough))

Thank you we have made the suggested change to the title.

4. The primary outcome was time to TB treatment. Is there a reason that the authors and investigators chose this as the primary outcome? Several studies have shown that even though new diagnostic approaches, like using Xpert instead of smear microscopy, have shortened the time to TB treatment, these approaches did not significantly improve patient important outcomes like morbidity or mortality.

Thank you. We selected time to TB treatment initiation as the primary outcome as it is an important programmatic indicator of the success of complex screening interventions. In primary care, where the prevalence of TB is low and mortality is very rare, the suggested alternative primary outcomes would have been infeasible to evaluate, although we did compare these and quality of life as secondary outcomes. We are currently conducting a further randomised trial to evaluated DCXR-CAD screening among hospitalised adults with HIV with a mortality outcome (https://clinicaltrials.gov/ct2/show/NCT04545164). We recommend that for the primary care use-case, future substantially larger cluster randomised trials may additionally wish to evaluate the effectiveness of these interventions against mortality and morbidity outcomes, as we note in the Discussion (Lines 475-477).

5. One of the secondary outcomes is undiagnosed or untreated TB. Is this because the authors could not differentiate between undiagnosed and untreated TB based on available data in the medical records? If the authors could differentiate diagnosis from treatment, wouldn't undiagnosed TB be the cleaner outcome of interest because the intervention is designed to increase diagnosis of TB? Were there cases where an individual was diagnosed but not treated?

This was because, based on previous research and systematic reviews, a substantial fraction of adults with microbiologically-confirmed TB (up to 18% in Africa, MacPherson et al 2013) do not initiate TB treatment. Therefore, this outcome captures both of these unfavourable outcomes – TB that is not diagnosed (i.e. missed by screening interventions), and TB that is diagnosed by not treated (i.e. pre-treatment loss to follow-up) – both of which we would hope the interventions would reduce. By the Day 56 outcome assessment, there were no participants with diagnosed but untreated TB.

6. In the second last sentence of the abstract, the abstract talks about undiagnosed/untreated HIV. Again, can the authors indicate whether (1) it was impossible to differentiate between undiagnosed and untreated HIV, or (2) this represents a combined outcome.

Thank you. As above this pre-specified combined outcome set out to record participants who were diagnosed with HIV, but not treated, a not uncommon scenario in routine care programmes. Given the small numbers of participants experiencing this outcome, we have not disaggregated further.

7. In the abstract, the intervention itself seems focused on screening for HIV and no mention of the linkage component is made. However, on line 136: it suggests that support for ART enrolment was provided as part of the intervention - this is important for the abstract and should ideally be included. E.g., "oral HIV testing (HIV screening) and linkage to treatment for those who screen positive."

Thank you – we have modified this sentence to include linkage to HIV treatment (Lines 34-37):

"Adults (≥18 years) with cough attending acute primary services in Malawi were randomised (1:1:1) to standard-of-care (SOC); oral HIV testing (HIV screening) and linkage to care; or HIV testing and linkage to care plus DCXR-CAD with sputum Xpert for high CAD4TBv5 scores (TB-HIV screening)."

8. In the conclusions of the abstract, the term "universal HIV testing" is used, but earlier in the abstract the term HIV screening is used. Wouldn't "universal HIV screening" more accurately reflect the intervention?

Thanks - we have made this change (Lines 54-55):

"DCXR-CAD with universal HIV screening significantly increased the timeliness and completeness of HIV and TB diagnosis."

9. In reporting the primary outcome analysis, it is a little unclear which is the primary outcome comparison - is it the median time to TB treatment? The hazard ratio analysis compares rates of TB diagnosis between arms. Can the authors clarify why the statistical test used was Cox proportional hazards ratio analysis when the primary outcome comparison is of medians [6,7]?

The primary outcome is time at which TB treatment is initiated. Since not everyone initiated treatment, we quantified this by presenting both the proportion who initiated treatment, and the time at which they experienced it, and then calculated hazard ratios and 95% confidence intervals from Cox regression which compares the hazard of TB treatment initiation (across all follow-up time, i.e. using all the survival time information from the whole Kalpan Meier curve) between trial arms. We have now additionally included results from a log rank test which compares TB treatment times directly. These results are consistent with those from Cox regression.

10. In the conclusion, does the term "completeness of diagnosis" mean completeness of both HIV and TB diagnosis? For clarity suggest adding the word "HIV and TB diagnosis".

Thank you. We have made this change (Lines 54-59):.

"DCXR-CAD with universal HIV screening significantly increased the timeliness and completeness of HIV and TB diagnosis."

**11. Page 8 - line 125 "we intended that the SOC arm would reflect the routine TB and HIV screening"
- To what extent is it truly possible to ensure that SOC stayed the same. Surely the healthcare workers would have been aware of the study and its hypothesis, and could this have affected SOC (i.e., improved quality) through a type of Hawthorne effect?**

Whilst this is possible, and we acknowledge this in the Discussion (Line 470-477), we took extensive steps to reduce the possibility of the Hawthorne effect. As note in response to Reviewer 2, Point 5 above, one alternative study to mitigate further against this would have been a clinic-level cluster randomised trial. This would have required a large number of clinics and would have been a substantially more expensive. Nevertheless, given the promising findings here, we do believe it would be warranted now to undertake such a trial.

12. Page 9 - line 148: How many of the suspect TB cases were identified by the consultant radiographer rather than the CAD4TB? Most primary healthcare clinics will not have ready access to a consultant radiographer, so the authorship team should ideally specify whether this radiologist checking the x-ray results was part of the intervention or not?

We did not consider the radiologist interpretation to be part of the trial intervention, but rather this was required as by the ethical review boards. Although it is difficult to say from the data, given the median time to TB treatment in the DCXR-CAD arm was only 1 day, and that all TB treatment initiations were microbiologically-confirmed, it is likely that the consultant radiologist read did not add additional TB treatment initiations. We have added a sentence to the limitations to discuss this (Lines 470-481):

"For safety reasons, remote radiologists (not usually available in similar primary care settings) provided a clinical interpretation of chest x-rays, although, given the very short median time to TB treatment initiation, this did not seem to influence TB treatment starts."

13. Page 11 -Line 181-182: As noted in the abstract comments, please can the authors explain and clarify the terminology "undiagnosed/untreated tuberculosis" and "undiagnosed/untreated HIV". The key questions are: (1) do these represent composite outcomes? (2) were the authors able to differentiate between diagnosis and treatment? And (3) given the intervention on the TB side, which seems focused on diagnosis and not linkage to treatment, is it appropriate to include the TB treatment component in the secondary TB outcome? If linkage to TB treatment was part of the intervention, this should be clarified in the abstract and methods section. If someone was diagnosed with TB but not treated for TB, would this contribute to the numerator of the percentages reported for each arm? Similarly, if someone was diagnosed with HIV but not treated for HIV, would this contribute to the numerator of the percentages reported for each arm? What is the rationale behind the decision the authors made?

Thank you. Yes, these are composite outcomes, comprising participants who were either not diagnosed with TB or HIV, or diagnosed but not treated for TB or HIV, respectively. Therefore, participants diagnosed but not treated for TB would contribute to the numerator of this secondary outcome. The reason for this was that there is substantial evidence from systematic reviews, and from primary data in Malawi and elsewhere showing that pre-treatment loss to follow-up can be very high for both TB and HIV. Linkage to TB treatment for those diagnosed as part of study activities (i.e. only in the DCXR-CAD + HIV screening arm), comprised of the research assistant escorting the participant from the study building located within the grounds of the clinic to the onsite TB clinic office with a copy of their sputum Xpert results.

14. Page 12 - cost-effectiveness analysis. Please include the following details in the main manuscript text in the methods section - without this information, it is hard to understand what the authors did. Since word limits are not a constraint, the following text should be included: "Malawi does not have formal cost-effectiveness threshold. We therefore compared the estimated ICERs to WHO-recommended thresholds using the gross domestic product (GDP) per capita for the country. Interventions that have an incremental cost per gain in QALY less than the national GDP per capita were defined as "very cost-effective", and those less than three times GDP per capita as "cost effective"11. The GDP per capital of Malawi is approximately US400 per capital. We therefore used the bootstrapped replications to present the probability the two interventions (HIV screening; TB-HIV screening) were cost-effective at increasing cost-effectiveness thresholds: US$400/QALY; US$800/QALY; US$1200/QALY. The probability represents the proportion of the 1000 bootstrapped replications where the estimated ICER was below these cost-effectiveness thresholds."

Thank you. We have now included this text in the Methods (Lines 224-234).

15. The Fig 1 did not show up well on the manuscript I reviewed.

We apologies for this and hope the revised Figure 1 will be clearer now.

16. Can the authors add the percentages for those seen/vital status and the % lost to follow-up?

We have added these percentages to Figure 1, and also included the overall % for seen/vital status in the results section (Lines 281-284).

17. Seems like close to 20% were lost to follow-up? Will need to address this potential source of selection bias and measurement error.

A total of 1320 (90%) participants had day 56 vital status ascertained, i.e. 10% loss to follow-up, and a total of 1206 (82%) gave samples and had day 56 TB/HIV outcomes ascertained. Thus loss to follow-up in relation to the primary outcome was 18%, and this was evenly distributed across the three trial arms. We have additionally compared baseline characteristics between participants who were and were not followed up, and these are similar (Supplemental Table 1),

with the exception that participant not followed up were on average slightly younger, and more likely to report unknown HIV status. We have added these data to Figure 1 and the body of the Results (Lines 279-287). We have additionally mentioned this in the Discussion (Lines 469-470):

"However, loss to follow-up was similar between arms, and characteristics of participants followed-up were similar to those lost to follow-up."

18. Line 219 - the problem of asymptomatic TB among the +-20% of clients living with HIV and mostly on ART needs to be considered in the discussion section.

Whilst we appreciate the growing research interest and have written extensively on asymptomatic TB, the large majority of research and burden estimates into asymptomatic TB come from secondary analysis of TB prevalence surveys conducted among randomly sampled community members. The population under investigation in the present trial was adults (HIV-positive and HIV-negative) with cough attending primary care with an acute illness. Given that all adults attending the clinic for an illness are likely to have a symptomatic illness that prompted their care-seeking, we feel that this population is not optimal for investigation of asymptomatic TB. This may be an important consideration for community-based active case finding interventions but is beyond the scope of the current manuscript.

19. Line 231 - 232: the CAD4TB approach screened in over two thirds of the population for GeneXpert (68%), but only 4% of the screened in population tested positive for microbiologically confirmed TB. A positive predictive value of only 4% among a group of symptomatic patients among whom HIV prevalence was 20%, seems low. Is the specificity of the CAD4TB approach too low? Do you have any data on TB prevalence among those who screened negative with the CAD4TB?

Thank you for these helpful comments. Unfortunately, due to the fact that sputum collection in participants with a low CAD4TB score would comprise an additional diagnostic test, potentially biasing outcomes, and the cost of undertaking TB culture (approximately US$30 per sample) and Xpert (approximately US$28 per sample in our setting) and the low probability of detection of microbiologically-positive TB (based on previous diagnostic accuracy studies) in participants with low CAD4TB scores, we did not undertake baseline sputum investigations among those who screened negative with CAD4TB. This could be considered for future studies.

20. Table 2: Can the 95% CI of the number initiating TB treatment be added?

We have now included 95% CIs for the proportion initiating TB treatment in each arm (Lines 289-291).

21. Line 276: the EuroQoL EQ5D utility score increases of 0.03 in both arms - this seems a small number. Is it possible to contextualize what a 0.03 means in the discussion section?

The best way is to interpret these figures are to extrapolate to the whole population over the timeframe of the trial. An EQ-5D utility increase of 0.03 over one year in equivalent to 3,000 QALYS gained per 100,000 people. We have added test to the results to add clarity (Lines 331-332):

"Mean EuroQoL EQ5D utility scores at day 56 were significantly higher in participants in the HIV-TB arm compared to SOC (adjusted mean difference [AMD]: 0.03, 95% CI: 0.004-0.06), and in participants in the HIV screening arm compared to SOC (AMD: 0.03, 95% CI: 0.001-0.05). If these differences were maintained for one year, at the population-level, this would result in 3,000 QALYS gained per 100,000 people."

22. The fact that the EuroQoL increases was 0.03 in both the HIV and HIV-TB arms, does this suggest that all the improvements were due to the HIV screening? Would the authors have expected a dose-response effect if additional improvements from undiagnosed TB on top of undiagnosed HIV were to be observed?

Economic evaluations aim to estimate differences in economic outcomes between two or more interventions and findings cannot be used to make inferences around what is driving these differences. However, we are currently conducting transmission modelling to investigate the potential health and economic impact of the interventions beyond the 56 day within-trial analysis reported here. These modelling studies will be reported separately.

23. Page 23: Note: the line numbering is absent from the Discussion

We apologies for this omission, and the line numbers have been added.

24. Page 23: Last sentence of first paragraph of the Discussion section: The following sentence should be revised to

indicate that the intervention helped detect symptomatic TB, and helped detect HIV among those with at least one symptom "cough". In addition the words "rapidly and efficiently" should be removed. E.g., the sentence could be edited to read: "If implemented at scale in primary care, DCXRCAD combined with oral HIV screening has potential to improve diagnosis and treatment of symptomatic TB and HIV among persons presenting to the health facility with cough.". The word "efficiently" needs to be deleted because (1) the paper did not assess the term "efficiency" from a costing perspective, and (2) from the cost-effectiveness analysis, the HIV-TB component was not cost-effective. If the HIV-TB screening component was not cost-effective, why would the authors consider it efficient? the word "rapidly" needs to be removed because the paper provided no evidence that either is shortened time for the patient at the clinic, or that DCXRCAD could be rapidly rolled out.

Thank you, we have made these recommended changes (Lines 357-360):

"If implemented at scale in primary care, DCXRCAD combined with oral HIV screening has potential to improve diagnosis and treatment of symptomatic TB and HIV among persons presenting to the health facility with cough."

25. Page 23: The authors state: "However, with nearly 60% of unselected acute care clinic attendees reporting TB symptoms,[7] the current recommended screening approach for TB is not reliably implemented, reflecting workload, turnaround time, affordability and throughput issues for sputum-based diagnostics." The authors then list perceived advantages of the chest x-ray screening approach. However, the authors do not provide a balanced view of the pros and cons of the chest radiography screening approach. There are substantial barriers facing widespread scale-up of the proposed intervention including:
(1) no abnormalities are definitive of TB and therefore specificity is low,
(2) special equipment with constant source of electricity needed,
(3) Equipment maintenance is needed which is a challenge in LMIC,
(4) Trained personnel are needed to operate the equipment,
The authors observed the intervention was not cost-effective in their own analysis.
The authors need to better describe the challenges associated with wide scale-up of the proposed intervention, especially in a very resource-constrained country like Malawi where only 10% of the population has access to electricity and less than 60% of health facilities have any supply of electricity. Even those facilities that have a supply of electricity have routine, unscheduled blackouts.

Thank you for these helpful comments, and we agree that deployment of DXCR-CAD can be challenging in low-resource settings. We have added additional text to the Discussion to highlight these important points (Lines 379-385):

"However, implementation of DCXR-CAD in high HIV/TB burden settings where they are most needed can be challenging, and requires careful consideration by Ministries of Health, as well as technological optimisation for low-resource settings and operational research to support deployment. Particular challenges can include absent or intermittent power supplies, limited internet availability, availability of maintenance and servicing personnel, and laboratory capacity to handle potential increased sputum specimen samples for confirmatory testing, although these were all overcome in this trial."

26. Page 23: The authors note that all persons attending these clinics should be screened for HIV and TB risk, given the very high prevalence of HIV. For those clients with HIV, the WHO 4-symptom screen is recommended. Instead of introducing more complicated TB screening approaches with chest radiography and other needs (e.g., stable electricity, maintenance, skilled personnel), what would be the cost and impact of improving current WHO-recommended approaches to screening for TB among PLHIV and HIV-negative persons by providing additional healthcare workers, and training in current algorithms? In at least one study, this was effective among PLHIV [8]. There is only limited discussion of other approaches to improving screening for TB, and a broader acknowledgement of other potential approaches (e.g., health system strengthening to improve implementation of current algorithms, and use of simple risk scores) could ideally be mentioned.

We disagree regarding the WHO 4-symptom screening test for HIV positive adults. The WHO 4-symptom screening test is a highly specific screening test used to rule out active tuberculosis to permit safe TB preventive therapy initiation, and is not a sensitive screening test for active TB. Instead, many countries, including Malawi, use a symptom screen of "cough of any duration" as a more sensitive initial screening test to guide further investigation for TB (e.g. sputum testing) in HIV-positive adults. In our (and many other researchers' studies) the prevalence of cough of any duration is extremely high among adults attending primary care with an acute illness. We have added additional text to describe alternative screening approaches (particularly C-reactive protein, and clinical risk prediction scores) that might be considered, and the importance of health system strengthening (Lines 395-398):

"Potential approaches to further reducing sputum-test demand include increasing the CAD4TB score used to define presumptive TB, or adding in a further rapid screening step such as point-of-care C-reactive protein, or clinical risk prediction scores.[29,30] These should be implemented and evaluated in conjunction with health system and laboratory strengthening."

27. The authors state that CAD4TBv6 has high specificity (84%), without mentioning the population in which these data come from - can this be added? In addition, over 60% of persons attending the clinic with cough screened into Xpert screening after CAD4TBv6 screening with only 4% yield. Do the authors have any data on sensitivity and specificity of the TB screening approach taken in this study?

Thank you. There have been a considerable number of studies that have evaluated the diagnostic accuracy of CAD systems for TB against microbiological and clinical reference standards (see systematic reviews referenced in the Introduction). Unfortunately, due to the cost of undertaking TB culture (approximately US$40 per sample) and Xpert (approximately US$28 per sample in our setting) and the low probability of detection of microbiologically-positive TB) in participants with low CAD4TB scores, we did not undertake sputum investigations at baseline among those who screened negative with CAD4TB. This would have also influenced the trial outcome, by potentially diagnosing TB earlier, and culture not part of routine care at the clinic.

28. In the concluding sentence, the authors state: "If effective in larger trials and under programmatic conditions, this approach has potential to rapidly and efficiently improve diagnosis and treatment of TB and HIV. Recommend removing the words "rapidly and efficiently" because the data in this paper do not support this.

We have made this recommended change (Lines 485-486):

"If effective in larger trials and under programmatic conditions, this approach has potential to improve diagnosis and treatment of TB and HIV."

Reviewer #4:

1. MacPherson and colleagues describe the results of the PROSPECT trial in Malawi. The trial is both impressive and informative despite not having observed as many endpoints as it needed to be well-powered for the result. The manuscript is very well-written. I especially commend the many ways that the authors went above and beyond to make the manuscript transparent and clear. It is really helpful to have the power analysis described, to help readers explain why the trial seemed to be adequately powered at the outset but ended up having so few events in the primary outcome. The Appendix is a terrific addition, and having the unit cost assumptions broken out in great detail is an excellent addition to the literature and a leg up to future researchers.

Thank you for these comments.

2. Major points: 1. The article presents both HIV and TB outcomes, but the HIV component is presented as just a footnote. The Introduction only discusses TB, providing no context about HIV in this setting. To balance this, consider moving some of the HIV background (such as local HIV prevalence and ART coverage) from Discussion to Introduction and consider ways to balance the other sections of the paper.

Thank you – we have added additional text to describe the burden of HIV in Malawi, and the Malawi national HIV programme (Lines 107-112):

"Bangwe health centre is located within a densely-populated neighbourhood in the East of the city. Adult HIV prevalence in Blantyre is estimated to be 18%,[20] and Malawi urban TB prevalence was estimated at 988 per 100,000 in the most recent national TB prevalence survey.[21] Comprehensive HIV care (including ART) and TB treatment are available at primary care level through the national HIV and TB treatment programmes in Malawi."

Importantly, although this study was conducted in a high HIV prevalence setting, the target populational included both HIV-positive and HIV-negative adults. A key finding of the trial was that the HIV screening intervention was effective, even though the coverage of ART was extremely high at baseline. We think that these results are important for HIV programmes in high HIV-prevalence settings, and discussed these in Lines 436-448 of the Discussion.

3. There was a statistically significant reduction in undiagnosed HIV on Day 56, but this finding does not seem to jive with the reported HIV diagnoses leading up to Day 56 (20, 25, and 15 new diagnoses in the SOC, HIV, and HIV-TB arms). How can one reconcile the finding of similar numbers diagnosed through Day 56 in each arm, and yet more undiagnosed cases of HIV on Day 56 in the SOC arm (10 compared to 2 and 1 in the other arms)? One would expect, if the arms were balanced in terms of number with undiagnosed HIV, that more HIV diagnoses would need to occur in the HIV and HIV-TB arm in order to "deplete" those arms of undiagnosed PLHIV.

The figures given for new HIV diagnoses (20, 25 and 15 new diagnoses in the SOC, HIV and HIV-TB arms) include both those who were newly diagnosed during the follow-up period, and those who were newly diagnosed at the day 56 visit.

Therefore, these figures include the 10, 2 and 1 participants, in the SOC, HIV and HIV-TB arms, respectively, who were ascertained at the day 56 visit to have previously undiagnosed/untreated HIV infection. We have edited the text to clarify that the figures of 20, 25 and 15 include those newly diagnosed with HIV throughout follow-up, including the day 56 visit.

4. It is surprising that a majority of patients received a positive result from the triage test at the cut-off value used in this study, yet the actual prevalence of Xpert-positive TB was quite low, given the previously published CAD4TB performance of 91% sensitivity and 84% specificity. Could the authors calculate the specificity of the CAB4TB test that was observed in the trial? It would also be helpful if the Discussion could provide any insights into why the specificity might be so much lower than expected.

Unfortunately, due to the cost of undertaking TB culture (approximately US$40 per sample) and Xpert (approximately US$28 per sample in our setting) and the low probability of detection of microbiologically-positive TB) in participants with low CAD4TB scores, we did not undertake sputum investigations at baseline among those who screened negative with CAD4TB. This would have also influenced the trial outcome, by potentially diagnosing TB earlier, and is not part of routine care at the clinic. Therefore, we cannot calculate specificity of the CAD4TB system in this trial. We have added text to the Discussion to describe this, and emphasise that, if DCXR-CAD is widely implemented, regular assessment of diagnostic accuracy is required (Lines 448-452):

"We did not estimate the diagnostic accuracy of DCXR-CAD, as baseline sputum samples were not collected from participants with a low CAD4TB score; if DCXR-CAD is widely implemented, surveillance of accuracy at DCXR-CAD thresholds using microbiological and clinical reference standards should be part of routine monitoring and evaluation activities."

5. Relatedly, the paper could provide more information about co-occurrence of TB and HIV in the cohort. What was the HIV and ART status of those who screened positive with the CAB4TB test? Did CAD4TB specificity differ by HIV status? ART status?

Among those screening positive with the CAB4TB test (n=305), 282 had HIV status at baseline ascertained, and of these, 84 (30%) were HIV positive, of whom 69 (82%) were on ART. In the CAB4TB<45 group, the corresponding numbers were 19/134 (14%) HIV positive, of whom 15 (79%) were on ART. In summary, participants who screened positive on CAB4TB were twice as likely to be HIV-positive compared to those screening negative on CAB4TB (p=0.002 from chi-squared test). As noted above, in this study, we cannot estimate specificity of CAD4TB.

6. Do the investigators have access to the raw CAB4TB score (rather than just the binary outcome of whether or not it met the cutoff)? It could provide insight if the authors could tabulate (or show in a bar graph) the number of patients with different CAB4TB scores colored according to whether they had a positive Xpert, negative Xpert, or did not receive Xpert testing after the x-ray. Augmenting the table/graph with symptom and smear data stratified by CAB4TB score may also help inform the critical issue of specificity.

Thank you for these suggestions. We have undertaken the suggested analysis (where possible), and these are summarised in the Figure below. Although we do think these data are interesting, they are somewhat limited by the fact that we can't estimate specificity, and that these analyses are not the primary focus of this trial. We are therefore happy to be guided by the Editor and Reviewer as to whether these should be included.

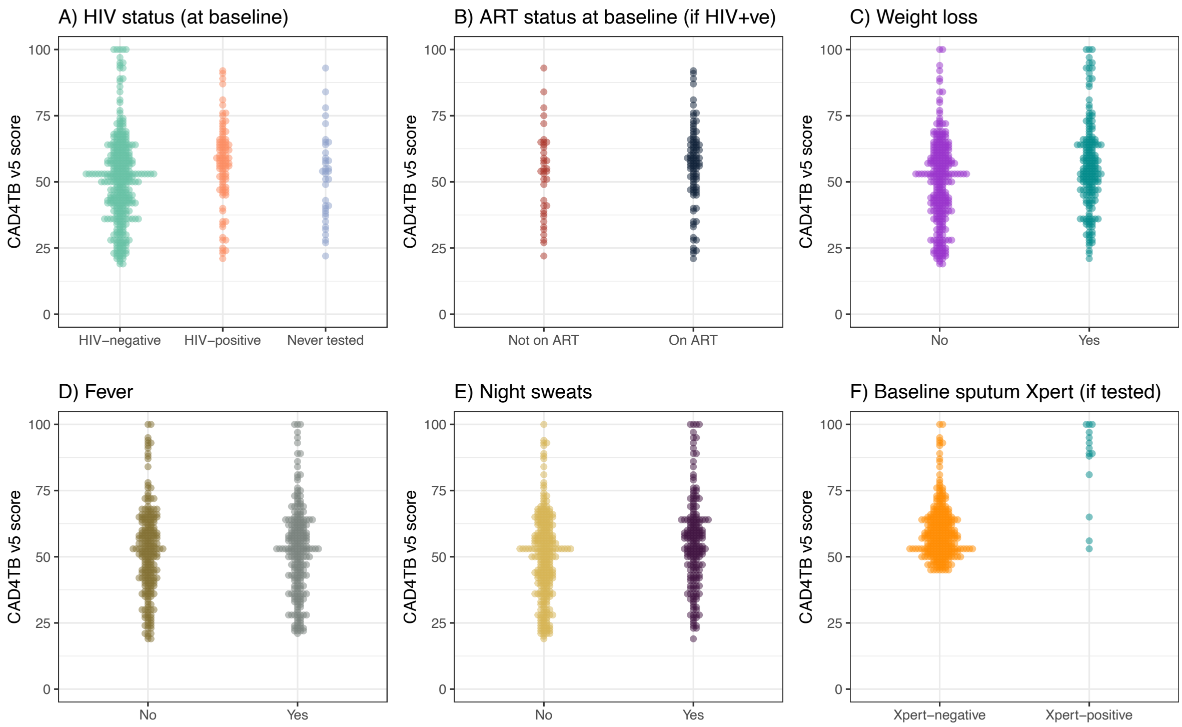

7. Cost-effectiveness analysis appears (at least based on what I understood from the Appendix) to only take into account the EuroQoL results, but in general one would expect that the main health benefit of prompt diagnosis would be later avoidance of critical illness and death, with only a small proportion of the effect being immediate quality of life after diagnosis. Is there a way to up-weight or forecast undiagnosed HIV and TB cases to account for future QALYs? Many published mathematical models make assumptions about future mortality risk for HIV and TB accounting for prompt vs delayed diagnosis; some of these models are open-source and available for download free of charge

Many thanks for this suggestion. Here, we report results of the pre-specified within-trial economic evaluation and therefore the time frame for the economic analysis has to correspond to the main effectiveness analysis (up to Day 56). Nevertheless, we are undertaking transmission modelling to investigate the health and economic impact of intervention implementation over a longer horizon. These analyses require adaption of our TB transmission models and calibration to local epidemiology, and will be reported separately.

8. The abstract uses the label "TB-HIV screening" but elsewhere in the manuscript it is referred to as "HIV-TB screening." Helpful to use consistent terminology.

Thank you. We have corrected this.

9. "Undiagnosed/untreated" -- unclear if this means "undiagnosed OR untreated" or if it means "undiagnosed AND untreated." Please specify. If it is "OR" consider simply stating "undiagnosed" since all indiagnosed individuals would be untreated.

Thanks – we mean "undiagnosed and untreated TB" here. We do think the distinction is important, as a substantial body of research shows that a large proportion of adults diagnosed with microbiologically-confirmed TB do not go on to initiate TB treatment (e.g. see MacPherson et al 2013, where 18% of adults with microbiologically-confirmed TB in Africa did not initiate TB treatment).

10. This sentence is difficult to understand -- "HIV screening reduced the proportion with undiagnosed/untreated HIV…"

Thank you. We have reworded to read:

"HIV screening reduced the proportion with undiagnosed or untreated HIV"

11. "which can approach 60%" -- of all attendees?

Yes, that is correct, as found in the referenced manuscript and also in more recent work from our study site (Feasey et al, BMC Infectious Diseases 2021).

12. Would be helpful to provide context and references/rationale for the CEA thresholds used.

Thank you. We have added text to provide more details here, and have also referenced the WHO Commission on Macroeconomics and Health[1], from whose guidance we selected CEA thresholds (Lines 224-234):.

"Malawi does not have formal cost-effectiveness thresholds. We therefore compared the estimated ICERs to WHO-recommended thresholds using the gross domestic product (GDP) per capita for the country. Interventions that have an incremental cost per gain in QALY less than the national GDP per capita were defined as "very cost-effective", and those less than three times GDP per capita as "cost effective". The GDP per capital of Malawi is approximately US400 per capital. We therefore used 1000 bootstrapped replications to present the probability the two interventions (HIV screening; HIV-TB screening) were cost-effective at increasing cost-effectiveness thresholds: US$400/QALY; US$800/QALY; US$1200/QALY. The probability represents the proportion of the 1000 bootstrapped replications where the estimated ICER was below these cost-effectiveness thresholds."

1. WHO Commission on Macroeconomics and Health: **Macroeconomics and health: investing in health for economic development. Report of the Commission on Macroeconomics and Health: Executive Summary.** *Geneva, World Health Organization* 2001.

13. Methods. The ART linkage intervention should be specified. "HIV-positive participants were supported to register for ART at the online clinic." -- what does the support entail?

Apologies, but the reviewer may have misread this text. We wrote: "HIV-positive participants were supported to register for ART at the **onsite** clinic" (not "**online**").

The linkage support included the study research assistants walking with the participant from the study research building to the ART clinic within the clinic grounds, and making a treatment assessment appointment with clinic ART providers (in practice, they were usually assessed on the same day, as per Malawian guidelines). We have added text to the methods to provide details (Lines 151-154):

"HIV-positive participants were supported to register for ART at the onsite clinic by walking with them to the ART clinic registration area and making an appointment for treatment initiation assessment by the ART clinic staff (in practice usually done on the same day, in accordance with Malawi guidelines)."

14. The method of recall of participants whose X-ray was flagged by the radiologist should also be specified.

Thank you – we have added text to the methods to describe (Lines 167-169):

"Digital chest X-rays were additionally read remotely by a Consultant Radiologist, and participants with abnormal findings identified were recalled by telephone and referred to the clinic or city Central Hospital with results."

We have also added text to the Discussion to describe this potential limitation (Lines 477-480):

"For safety reasons, remote radiologists (not usually available in similar primary care settings) provided a clinical interpretation of chest x-rays, although, given the very short median time to TB treatment initiation, this did not seem to influence TB treatment starts."

15. Has the EuroQoL EQ5D score been validated in Chichewa? Please site validation studies.

The Chichewa version of the EuroQoL EQ-5D-3L instrument was previously translated and validated in accordance with the EuroQoL group procedures in 2012. The Chichewa version we used in our trial is the groups' approved version.

16. It isn't clear why linear regression is used to analyze the change in EQ5D by arm. What is the independent variable?

EQ-5D is a continuous variable that can take values between 0 and 1 – we have added clarification of this in the methods. Thus linear regression is an appropriate technique for assessing the impact of the trial interventions on EQ-5D at day 56,

adjusting for baseline EQ-5D (essentially, analysis of covariance). As is recommended for within-trial economic analysis we used multiple imputation and seemingly unrelated multivariable regression analysis to estimate the incremental costs and QALYs gained over the 56-day trial horizon between the intervention arms.[1] We control for the main socio-demographic variables. For EQ-5D we also include the baseline EQ-5D utility score in the model as this will impact on potential improvements in EQ-5D utility score from receiving the intervention.[2]

1. Willan et al (2004). Regression methods for covariate adjustment and subgroup analysis for non-censored cost-effectiveness data. Health Econ. 13: 461–475
2. Manca et al (2005). Estimating mean QALYs in trial-based cost-effectiveness analysis: the importance of controlling for baseline utility. Health Econ. 14: 487–496

17. Typo: "CD4TB" rather than "CAD4TB"

Thank you. We have corrected this.

18. Results: What was the reason for the large drop-off in terms of screen-to-enroll ratio?

The main reason was non-eligibility, predominately not having cough. This is reported in Figure 1 (CONSORT Flow Diagram)

19. Important to qualify claims like "participants in the HIV-TB screening arm were more likely to have initiated TB treatment..." since this was not a statistically significant trend.

Thank you – we have modified this sentence:

"A greater percentage of participants in the HIV-TB screening arm (15, 3.0%) initiated TB treatment by day 56 compared to participants in the HIV screening arm (8, 1.6%) or SOC (5, 1.1%) – Table 2"

20. Figure 1 (Trial Profile) did not render correctly in the PDF. What I'm able to see (but these might be incorrect due to the rendering issue) is of n=8,236 screened, n=6,773 were "excluded" (why?), n=159 say "Multiple inclusion criteria not met" (what if only one is not met?), n=70 "did not consent" and n=1 "Withdrew before randomization." Need info on why the 6,773 were excluded and what happened with the remaining 1,224.

We apologies that the Figure did not properly render on the publisher's software platform. We have reattached the updated Figure and is hopefully clearer now. The detailed reasons for exclusion are fully reported in this CONSORT Flow Diagram, and answer the reviewer's comments.

21. Figure 2 would be helpful to overlay statistics on the figure itself.

Apologies. We are not clear what statistics the reviewer is asking to be added here. Comparisons of intervention arms are fully reported in Table 2 and in the text.

22. Discussion: Would be helpful for Discussion to address why it might be that the rates of undiagnosed TB on day 56 were similar across arms, despite 2-3x more TB diagnosis and treatment between arms. Why didn't the intervention reduce prevalence of undiagnosed TB on day 56? For example, do you suspect much of the undiagnosed TB in the SOC arm may have self-resolved? Or treatment may have been unsuccessful in the HIV-TB arm?

We are not sure why this is the case, and don't have any data to support any particular theories. We have added text to the discussion to emphasise this point (Lines 461-464):

"Although more participants initiated TB treatment in the HIV-TB screening arm compared to the other arms, the proportion with undiagnosed TB was similar across arms. It is not clear why this is the case, but may be due to chance with small numbers of events here."

23. Typo: "target produce profiles" -> target product profiles

Thank you – we have corrected this typo.

Reviewer #5:

Thanks for the opportunity to review your manuscript. My role is as a statistical reviewer, so my review is focused on the study design, data, and analysis (and the presentation of these). I have put general comments about the

manuscript first, followed by comments specific to a section of the manuscript (with line numbers). This trial compares two treatments (oral HIV tests, and HIV test + computer aided chest x-rays) with usual care in adults with cough attending primary health services in Malawi. Two key strengths are the study is the large size of patients enrolled, and how the study was embedded within normal clinical practice.

1. My first concern is about the final event rate (TB treatment initiated) in the study. The sample size for the study was based on 17% of patients initiating TB treatment, with a reasonably large effect size (HR of 1.5). The study recruited ~ 500 participants per arm (~1500) total and saw TB treatment in 28 participants (~2% initiated TB treatment). There is a 'significant'' difference between the HIV-TB screening arm and usual care, but the estimated upper limit of the 95% CI for this effect is 7.87. How should this effect be interpreted given this very high upper limit? A relatively small missing-not-random effect could diminish the difference very quickly - how robust is this main finding given the drop-out rate observed? The lower event rate is mentioned as a limitation of the study but this is only mentioned in the context of sub-group analyses not performed (I agree that this was a good decision). At the very least this should be discussed directly in the context of the assumed event rate in the sample size calculation. The Cox method is a robust for large samples, but I am also concerned that the very small number of events makes this inappropriate with the data collected. There are proposed modification of the log-rank test that should be more robust in these situations (e.g. Mehrotra + Roth, Stats in Medicine 2001: doi: 10.1002/sim.854.) that would be appropriate to apply here to ensure the event-rate does not cause a bias in the hazard ratio.

It is true that the primary outcome event rate was lower than anticipated. We have now expanded on this as a limitation in the discussion, stating that the fewer-than-expected number of events results in a loss of precision in treatment estimates, and that this means that our findings may not be robust to any differential missingness between groups (Lines 466-470). However, losses to follow-up were similar between trial arms, and characteristics of those followed-up were similar to those not followed-up.

As the reviewer notes, the Cox method is robust for large samples. The sample size of our study is large, so the only concern to address is with the event rate. We feel that the number of events (n=28) is sufficiently high for the Cox model to produce unbiased estimates of the regression coefficients, and for the calculated confidence intervals to give the required coverage. We base this on findings from a simulation study conducted by Peduzzi et al[1] which found that Cox regression performed well as long as the number of events per model parameter was greater than 10, i.e. substantially less than the 28 events we observed in our study. Therefore, we feel that the Cox method is an appropriate approach here. Thank you for referring us to the Mehrotra and Roth paper. On reviewing this paper, we are also not convinced that the proposed modification of the log rank test will be more robust for our dataset. The authors suggest that the generalised log rank approach should be avoided in situations where there are heavy ties. In our study, 6 of the 28 participants who initiated treatment did so on the day of their baseline visit, and there were also several who initiated the following day. Therefore, we are concerned that if we attempted to apply this test, we would be introducing a new issue into the analysis.

1. Importance of events per independent variable in proportional hazards regression analysis II. Accuracy and precision of regression estimates, Journal of Clinical Epidemiology 1995, 48(12): 1503-1510)

2. Was the study prospectively registered? Was a SAP created? Having access to the SAP would help with the review process.

Yes, the trial was prospectively registered, as reported in the Abstract and Methods. (clinicaltrials.gov: NCT03519425). The statistical analysis plan was prospectively written before trial initiation, and was uploaded with this submission. We have reattached as supplemental material again.

3. With regards to the main outcome, what is the interpretation of comparisons against the oral HIV test arm? Is this interpreted as a more 'active' control?

Thanks. In this comparison we are assessing the additional benefit of adding the TB screening interventions (DCXR-CAD) to the HIV screening intervention for the trial outcome.

4. L113. Was block randomisation used? One arm (497) has 24 more participants than one of the other study arms (473)

We did not use block randomisation because we anticipated that (1) the large sample size would ensure that approximately equal numbers of participants would be randomised to each arm, (2) any differences that occurred by chance in numbers allocated to each group would have minimal impact on power, and (3) following the guidance summarised by Schultz and Grimes,[1] "no other approach, irrespective of its complexity and sophistication, surpasses simple randomisation for prevention of bias".

1. Schulz KF, Grimes DA. Generation of allocation sequences in randomised trials: chance, not choice. Lancet. 2002 Feb 9;359(9305):515-9. doi: 10.1016/S0140-6736(02)07683-3. PMID: 11853818.

5. L183. What exact type of binomial models were these? i.e. what link function was used in the generalised linear model?

A log link function was used in the generalised linear model, i.e. these were log-binomial models. We have added this information to the statistical methods section (Line 210).

6. L183. Three pairwise-comparisons between the three arms were made - was any adjustment for multiplicity performed?

There are conflicting schools of thought on whether the type-1 error rate should be adjusted in the event of more than two trial arms being compared, as summarised by Schulz and Grimes.[1] Since this is a pragmatic trial rather than a confirmatory or regulatory trial and is not operating in a purely hypothesis-testing paradigm, we felt that any adjustment would be overly stringent. Therefore, as specified in the protocol and analysis plan, we chose not to make any formal adjustment for multiple testing, but instead chose to clearly pre-specify exactly which groups would be compared, i.e. three pairwise comparisons: (1) HIV-screen versus SOC to assess impact of HIV-screening intervention, (2) HIV-TB screening versus SOC to assess the combined effect of an HIV and TB screening intervention, and (3) HIV-TB screening versus HIV-screening to assess the additional impact of TB-screening when applied on top of HIV-screening. Clearly, the last comparison is correlated with the first two comparisons, but we felt it important to show these results as well to aid with interpretation.

1. Schulz KF, Grimes DA. Multiplicity in randomised trials I: endpoints and treatments. Lancet. 2005 Apr 30-May 6;365(9470):1591-5. doi: 10.1016/S0140-6736(05)66461-6. PMID: 15866314.

7. L187. How was this adjustment made? By change score or including baseline value as a covariate?

Thanks. Adjustment was made by including baseline value as a covariate as recommended in Manca et al.[1] We described this in Lines 212-214:

"To evaluate the effect of interventions on health-related quality of life, we used linear regression to compare the mean EQ5D utility scores measured at day 56 between pairs of arms, adjusting for participants' corresponding values measured at baseline."

1. Manca et al (2005). Estimating mean QALYs in trial-based cost-effectiveness analysis: the importance of controlling for baseline utility. Health Econ. 14: 487–496

8. L187. It looks like approximately 256 participants in total were able to be contacted at day 56. How was this missing data treated? Were these censored in the main analysis or were they excluded?

For the main analysis, these 256 participants were censored at the last point of contact. We have included this information in the statistical methods.

9. L187. How were the assumptions in the models checked (e.g. PH assumption for Cox model, distribution of residuals in linear regression?)

We constructed log-log plots and checked that curves were parallel and also conducted tests of Schoenfeld residuals to assess the proportional hazards assumption. We found no evidence that the assumption was violated. For linear regression, we plotted model residuals and constructed QQ plots. We have now included this information in the statistical methods section (214-215).
