# Supplementary material for: Computer-aided X-ray screening for tuberculosis and HIV testing among adults with cough in Malawi (the PROSPECT study): A randomised trial and cost-effectiveness analysis
Source: PLoS Med. 2021 Sep 9;18(9):e1003752. doi: 10.1371/journal.pmed.1003752 (PMC8459969; doi:10.1371/journal.pmed.1003752)
Supplement: Attachment — Submitted filename: 2021-07-23_response-R2_PROSPECT.docx [file pmed.1003752.s009.docx]

**Dr Peter MacPherson MBChB MPH PhD**
Wellcome Trust Fellow & Reader in Population Health
Liverpool School of Tropical Medicine &
Malawi-Liverpool-Wellcome Trust Clinical Research Programme, Blantyre, Malawi

Honorary Professor in Population Health
London School of Hygiene and Tropical Medicine

Honorary Consultant in Communicable Disease Control
Public Health England

Mob: +265 99 717 6230
Email: [peter.macpherson@lstmed.ac.uk](mailto:peter.macpherson@lstmed.ac.uk)

28/07/2021

Dear Callam Davidson (Associate Editor, PLOS Medicine)

**Re: PMEDICINE-D-20-05974R2. Computer-aided X-ray screening for tuberculosis and HIV testing: a randomised trial and cost-effectiveness analysis in Malawi (PROSPECT)**

I write on behalf of my co-authors to thank the reviewers for their careful review and appreciate the opportunity to respond with a further revised submission.

Please see point-by-point responses below.

Yours Sincerely,

Peter MacPherson

(On Behalf of the Authors

**Requests from the Editor:**

**1. Please update your title to ‘Computer-aided X-ray screening for tuberculosis and HIV testing among adults with cough in Malawi (the PROSPECT study): a randomised trial and cost-effectiveness analysis’.**

Thanks. We have made this change.

**2. Please ensure that all numbers presented in the abstract are present and identical to numbers presented in the main manuscript text (e.g. line 52 ‘83%’ is 83.9% on line 321).**

We apologies for this typo and have checked and corrected throughout the manuscript.

**3. Line 38: Please either update to ‘Secondary outcomes included’ if opting to only include the main outcomes, or list and report all of your secondary outcomes.**

We have updated the abstract text to "Secondary outcomes included.." as suggested.

**4. Please specify who was blinded/masked.**

We have added text to Lines 42-44 to specify this:

*“In this open, three-arm randomised trial, adults (≥18 years) with cough attending acute primary services in Malawi were randomised (1:1:1) to standard-of-care (SOC); oral HIV testing (HIV screening) and linkage to care; or HIV testing and linkage to care plus DCXR-CAD with sputum Xpert for high CAD4TBv5 scores (HIV-TB screening). Participants and study staff were not blinded to intervention allocation, but investigator blinding was maintained until final analysis.”*

**5. Please state that analysis was intention to treat.**

We have added a sentence Line 47 to state this:

*“Analysis was done on an intention to treat basis.”*

**6. Please provide the number of participants lost to follow up in each group.**

We have added text to provide these numbers on Lines 50-51.

*“Between 15/11/2018-27/11/2019, 8236 were screened for eligibility, with 473, 492, and 497 randomly allocated to SOC, HIV, and HIV-TB screening arms; 53 (11%), 52 (9%), and 47 (9%) were lost to follow-up, respectively”*

**7. In the last sentence of the Abstract Methods and Findings section, please describe the main limitation(s) of the study’s methodology.**

We have added text to describe the main limitations on Lines 61-67..

*“Main limitations were the lower than anticipated prevalence of tuberculosis and short participant follow-up period; cost and quality of life benefits of this screening approach may accrue over a longer time horizon.”*

**8. At this stage, we ask that you include a short, non-technical Author Summary of your research to make findings accessible to a wide audience that includes both scientists and non-scientists.**

We have now included an Author Summary.

**9. Please provide titles and legends for all figures (including those in Supporting Information files).**

We have included titles and legends for all figures

**10. Please provide the unadjusted comparisons as well as the adjusted comparisons in Table 2**

We have included unadjusted comparisons for EQ5D utility score in Table 2, alongside the adjusted comparisons which controlled for baseline EQ5D score. For all other outcomes, only unadjusted comparisons are shown, as per the statistical analysis plan.

**11. Please update the CONSORT checklist to use section and paragraph numbers, rather than page numbers.**

We have updated the CONSORT Checklist in this requested format.

**12. Please add the following statement, or similar, to the Methods: "This study is reported as per the Consolidated Standards of Reporting Trials (CONSORT) guideline (S1 Checklist)**

We have added this text to Lines 273-274.

**13. Per CONSORT guidelines, please describe how investigator masking was maintained until after statistical analysis.**

We have added text to Lines 275-277 to describe this:

*“To maintain investigator blinding, no inspection of data by trial arm was done until data cleaning was complete and the database locked; the trial statistician then undertook unblinded analysis of the locked trial database.”*

**14. Please also define "lost to follow-up" as used in this study.**

We have added a sentence to define lost to follow-up in Lines 250-251.

*“Lost to follow-up was defined as participants who did not attend their clinic day 56 outcome assessment appointment and could not be traced to home.”*

**15. Other reasons for exclusion should be defined. Please reference these when updating the CONSORT checklist.**

The reasons for exclusion are provided in Lines 185-187:

*“We excluded individuals who had taken TB treatment in the preceding six months, who were currently taking TB preventive therapy, or who planned to move out of Blantyre.”*

We have updated the CONSORT Checklist accordingly.

**16. Please report your cost-effectiveness analysis according to the CHEERS checklist provided at** [**https://www.equator-network.org/wp-content/uploads/2013/04/Revised-CHEERS-Checklist-Oct13.pdf**](https://protect-eu.mimecast.com/s/mqcFCpkpmIznG9XSqzjC7?domain=equator-network.org)**. Please provide a copy of the completed checklist as a supplementary file and reference this in the Methods (and when completing, use section and paragraph numbers rather than page numbers).**

We have now included a completed CHEERS checklist as a supplementary file in the format requested.

**17. Please include the study protocol document, with any amendments, as Supporting Information to be published with the manuscript if accepted**

We have included the trial protocol as supplemental information. There were no amendments to the protocol during the study.

**18. On line 312, please clarify what you mean by "significantly". If statistical significance is intended, please provide the relevant p-value.**

Thank you. Yes, we mean statistically significant at the p<0.05 level, as indicated by the 95% confidence intervals. To provide additional clarity, we have additionally now added p-values throughout.

**19. Citations should be in square brackets, and preceding punctuation.**

We have changed these, as requested.

**20. Please remove the ‘Role of the funding source and data availability’ section from your methods – in the event of publication this information will be published as metadata based on your responses to the submission form.**

We have removed this section.

**Reviewer #1:**

**1. The enhanced arms (HIV screening and HIV-TB screening ) depended on trained study staff whilst the SOC depended on routine ministry of health staff. This limitation was pointed out a previous reviewer. The outcome of the enhanced arms could have been influenced by use of dedicated study staff. Dedicated staff should have been used for SOC arm too**

We thank the Reviewer for this comment, but respectfully take a different view. This was a pragmatic trial, intended to evaluate the effectiveness and cost effectiveness of the trial interventions **against current standard of care in the study clinic.** By using dedicated study staff in the standard of care arm, usual practice would have been changed, meaning that we would have been unable to determine the effectiveness of interventions compared to how TB/HIV diagnosis is usually delivered in the study clinic.

**2. When done well, symptom screening can result in increased TB case detection in PLHIV, as previously pointed by another reviewer, the study should have included this with dedicated staff to provided the opportunity for comparison with CAD TB screening. This is especially important in settings that will in the foreseeable future still have very limited access to digital x-rays. CAD systems also come at a cost and thus we do not expect increased access to CAD systems immediately despite the new recommendations from WHO**

Thank you for this suggestion, and we agree that a further trial comparing systematic symptom screening with CAD-based would be warranted. We respectfully disagree that CAD roll-out will be very limited for the foreseeable future. In addition to the new favourable recommendations from WHO in the most recent TB screening guidelines, for example in Blantyre, Malawi, where this study was done, digital chest X-ray with CAD has now already been widely deployed by the Ministry of Health through the National TB Programme in hospitals, in several primary health clinics, and in community-based active case finding vans.

**3. Ascertainment of mortality and lost to follow up is not well described in the methods, were participants asked to visit the facility at day 56? or did they come through at predefined follow up time points? And those lost to follow up, any effort made to determine reason, could some be mortalities?**

Thank you for this comment. This was reported in Lines 245-250 of the Methods, where we described procedures for follow-up and tracing:

*“Participants were seen at the study clinic at 56 days, when they underwent questionnaire and inspection of health records to determine TB treatment status, HIV testing (if not known to be HIV-positive and taking ART) and sputum collection for Xpert, MGIT culture and smear, with samples analysed at the TB Research Laboratory at the College of Medicine, University of Malawi. Participants who did not attend the clinic for this outcome assessment were traced to home, where possible.”*

We have now also added a further sentence to the methods defining "lost to follow up" as used in this study (Lines 250-251):

*"Lost to follow-up was defined as participants who did not attend their clinic day 56 outcome assessment appointment and could not be traced to home"*

As participants who were lost to follow-up were not seen at the clinic at day 56, and not able to be traced to home, we were unable to determine reasons for loss to follow-up. It is possible that some participants who were lost to follow-up could have died. We have added a sentence to the Limitations paragraph (Lines 566-567) to state this:

*“Some participants who were lost to follow-up may have died.”*

**4. Were any of the mortalities among those who were initiated on TB treatment? If so, though numbers are small, were they any differences that might indicate some differences in the different arms?**

No participants who initiated TB treatment during study follow-up died before their outcome assessment visit. Given the relatively small numbers of TB treatment initiations across the three trial arms, a substantially larger study would be required to answer this interesting question.

**Reviewer #2:**

**1. I have read the detailed responses of Dr MacPherson on behalf of his co-authors to the 5 reviewers of this submission. I am satisfied with the responses which show an appreciation of the issues raised by the reviewers and the limitations of the single-site Bangwe Clinic study. The results and conclusions of this necessarily limited study remain valid and the need for a larger and more robust study design in settings with different epidemiological and health system characteristics has been recognized. I think the updated manuscript has addressed the reviewers comments and can be published without further revision.**

Thank you for the review and supportive comments.

**Reviewer #3**

**1. Thanks for the opportunity to review the revision. Thanks to the authors for making many of the suggested changes. I have no additional comments.**

Thank you.

**Reviewer #4:**

**1. The authors requested guidance about whether to include in the paper the additional data on HIV and ART status by CAB4TB score, as well as the new figure showing the distribution of numeric scores by important strata. These do seem appropriate to incorporate into the manuscript. The figure should use bar graphs or line graphs showing the number or proportion of participants with each score. It is difficult to ascertain the density from the jittered dots in the graphs provided.**

Thank you. We have updated this figure to additionally show summary distribution statistics (box and whisker plot), density (density plot) and raw data (points), and have now included it within the main manuscript.

**2. Please cite the 2012 validation study of the EuroQoL in Chichewa**

We have added references.

**3.  Please qualify with something like "but this trend was not statistically significant."**

We have added the requested text.

**Reviewer #5:** 

**1. Thanks for the revised manuscript. Your manuscript had had a thorough set of reviews and I appreciate the thoughtful replies to my original queries.**

Thank you for the review and supportive comments.

**2. Unrestricted randomisation is fine - I would just add a few words as a qualifier in the info about randomisation (e.g. L123).**

*Thanks. We have added the following text:*

*“Participants were individually randomised without restriction to one of three arms…”*

**3. The thee pairwise comparisons are ok as well - 3 pre-planned comparisons is perfectly reasonable, but I would just add a clarifying phrase to the methods stating that there was no adjustment for multiplicity.**

We have added text to specify this point (Lines 270-271):

*“… at 5% significance level, allowing for 5% loss to follow-up and with no adjustment for multiplicity of testing.”*

**4. Apologies for not picking up the SAP in the first round of reviews, this for the most part matches the analysis described in the manuscript. The main difference I noted was that there was a planned sensitivity analysis using MICE for missing outcomes was planned, but these aren't reported. This would provide reassurance that missing data (at least under the missing at random assumption) does not affect the main results. Going from p-values in the Supp table, it may look as though there are some key differences between the complete-case data and those lost to follow-up. Education/literacy and age are moderately different but the p-values reflect the sample size here more than any strong differences. A HIV-positive status was less likely in those lost to follow-up, but again this was not strong (13% vs 20%). Presenting the planned sensitivity analysis showing similar effects as the main analysis along with this descriptive data would settle the issue about missing outcome data in this study. Strictly speaking, censoring or excluding participants with missing outcome data means the analysis can't be considered 'intention-to-treat'.**

For the primary outcome of time to TB treatment initiation, participants who were lost to follow-up were included in the analysis but with their follow-up time censored at the time they were last seen. Therefore, multiple imputation was not needed for this outcome as there were no missing data. For the secondary outcomes that had any missing data, supplementary table 2 shows the results from multiple imputation analysis alongside those from the complete case analysis, demonstrating that the results from both analyses are very similar. Additional text has been added to the statistical methods and the results sections.

**5. For those who didn't make the Day 56 outcome assessment visit, what was the average (mean, median) length of follow-up? I wasn't clear from the description if there were interim visits between baseline and the Day 56 visit.**

There were no interim visits between baseline and the Day 56 visit, so it is not possible to calculate the mean length of follow-up for those participants who were lost to follow-up. For survival outcomes, these participants were censored at 0.5 days.
